# Upper troposphere aerosol formation enhanced by monsoon lofted pollution

**DOI:** 10.1038/s41467-026-75073-x

**Published:** 2026-08-12

**Authors:** Christina J. Williamson, Stephan Borrmann, Philipp Brauner, Charles A. Brock, Teresa Campos, Theodoros Christoudias, Francesco D’Amato, Antonis Dragoneas, Oliver Eppers, Shawn Honomichl, Matthias Kohl, Franziska Köllner, Agnieszka Kupc, Young Ro Lee, Ming Lyu, Georgia Michailoudi, Laura L. Pan, Anne E. Perring, Andrea Pozzer, Dylan Simone, Warren P. Smith, Holger Tost, Rei Ueyama, Kirk Ullmann, Ville Vakkari, Silvia Viciani, Aki Virkkula, Ralf Weigel, Sarah Woods, Christos Xenofontos

**Affiliations:** 1 Finnish Meteorological Institute, Helsinki, Finland; 2 Institute for Atmospheric and Earth System Research/Physics, University of Helsinki, Helsinki, Finland; 3 Max Planck Institute for Chemistry, Mainz, Germany; 4 Chemical Sciences Laboratory, National Oceanic and Atmospheric Administration, Boulder, CO USA; 5 Atmospheric Chemistry Observations & Modeling Laboratory, NSF National Center for Atmospheric Research, Boulder, CO USA; 6 Climate and Atmosphere Research Center, The Cyprus Institute, Nicosia, Cyprus; 7 National Research Council—National Institute of Optics (CNR-INO), Sesto Fiorentino, Italy; 8 Johannes Gutenberg University, Institute for Atmospheric Physics, Mainz, Germany; 9 Aerosol Physics and Environmental Physics, Faculty of Physics, University of Vienna, Vienna, Austria; 10 School of Earth and Atmospheric Sciences, Georgia Institute of Technology, Atlanta, GA USA; 11 Cooperative Institute for Research in Environmental Science, University of Colorado, Boulder, CO USA; 12 Department of Chemistry, Colgate University, Hamilton, NY USA; 13 Earth Science Division, NASA Ames Research Center, Moffett Field, CA USA; 14 Atmospheric Chemistry Research Group, Chemical Resource Beneficiation, North-West University, Potchefstroom, South Africa

**Keywords:** Atmospheric chemistry, Atmospheric chemistry, Environmental monitoring

## Abstract

The Asian Summer Monsoon (ASM) lofts air from polluted boundary layer regions to the upper-troposphere and lower-stratosphere. High concentrations of aerosols have been observed in ASM outflow, which influence climate by interacting with radiation and clouds and affecting atmospheric composition. Prior observations indicate that small particles form in ASM convective outflow. Laboratory and modelling studies hypothesise the importance of lofted ammonia in this process and growth of the particles to sizes where they can interact with radiation and clouds. Here we combine in-situ observation in ASM convective outflow with trajectory and Earth-system modelling to show the likely role of pollution in enhancing ASM outflow new particle formation. We show that the particles grow to sizes where they can interact with clouds and radiation, and are transported over large areas within the troposphere and stratosphere.

## Abstract (web summary)

New particle formation in Asian Summer Monsoon convective outflow is enhanced by lofted anthropogenic emissions. The particles formed grow to sizes where they can impact clouds and radiation and are transported in the troposphere and stratosphere.

## Introduction

The Asian Summer Monsoon (ASM) is characterised by convective uplift from the Tibetan Plateau, India and South-East Asia, bringing air from polluted boundary layer regions to the upper troposphere and lower stratosphere (UTLS)^1–8^. Lofted primary aerosols and gas-phase precursors enhance UTLS aerosol concentrations, observed in both the annually recurring aerosol layer in the UTLS known as the Asian Tropical Aerosol Layer (ATAL)^9,10^ and aerosols transported across much of the northern hemisphere^11,12^. These aerosols impact climate via radiative effects^9,13^, providing increased surface area for heterogeneous chemistry^14^, affecting the properties of UT cirrus clouds, either through homogeneous ice nucleation, or heterogeneous ice-nucleation of ammonium nitrate aerosol^14–18^, and affecting the properties of warm and mixed-phase clouds as cloud condensation nuclei (CCN) after descent^16^.

New particle formation (NPF) has been observed in the upper troposphere and lower stratosphere (UTLS), where low concentrations of larger particles create favourable conditions for nucleation^19–32^. The lofting of precursor vapours by convection and the unique conditions created by stratospheric air intrusions are both associated with UTLS NPF^21,22,26–28,33^. Upper tropospheric NPF in remote regions has been shown to contribute to boundary layer cloud condensation nuclei concentrations, thereby affecting cloud properties^25,27,30^. New particle formation has previously been observed in recent ASM convective outflow^12,13,34^, though the role of lofted pollution in this process, and the climate impacts of the particles formed, were not fully resolved.

Synergistic NH_3_-HNO_3_-H_2_SO_4_ nucleation, a fast UTLS nucleation and growth mechanism, has been proposed from laboratory experiments and modelling^15,16^. Prior observations of high mixing ratios of ammonia in ASM convective outflow^35,36^ support the idea that this mechanism is of relevance to ASM convective outflow, but this has not yet been verified by observations. Modelling studies predict that during the ASM, days with convection produce UT NPF rates that are one to two orders of magnitude higher than quiescent days, driven by NH₃ uplift^16^, and that globally, anthropogenic NH₃ emissions when combined with convective transport, enhance UTLS NPF rates^37^.

Particles form at only a few nm in diameter^38^ and must grow to diameters above around 60 or 100 nm to significantly influence cloud properties or interact with radiation respectively e.g.,^39–41^. Therefore, the growth rate of these particles is of critical importance in determining their survival probability and fate^42,43^.

We assess the impact of pollution on particle formation in the upper troposphere by analysing in-situ measurements from two aircraft campaigns, the Asian Summer Monsoon Chemical and Climate Impact Project (ACCLIP)^44,45^ and Stratospheric and upper tropospheric processes for better climate predictions (StratoClim)^34,46,47^, combined with integrated trajectory modelling and satellite observations to diagnose the history and fate of the observed airmasses, and targeted Earth-system model sensitivity studies. We show that NPF occurs with high frequency and intensity in ASM convective outflow within the first two days following convection, and that this phenomenon occurs over the entire ASM region. We provide evidence that pollution lofted by the ASM likely increases the number of particles formed in the upper troposphere (UT), and that newly formed particles grow within the ASM region to sizes where they can influence climate in about a week. We demonstrate that this phenomenon is regional in scale and is associated with increased aerosol optical depth (AOD) and CCN concentrations.

## Results and discussion

### Regional Scale New Particle Formation in Asian Summer Monsoon Outflow

**Fig. 1:**
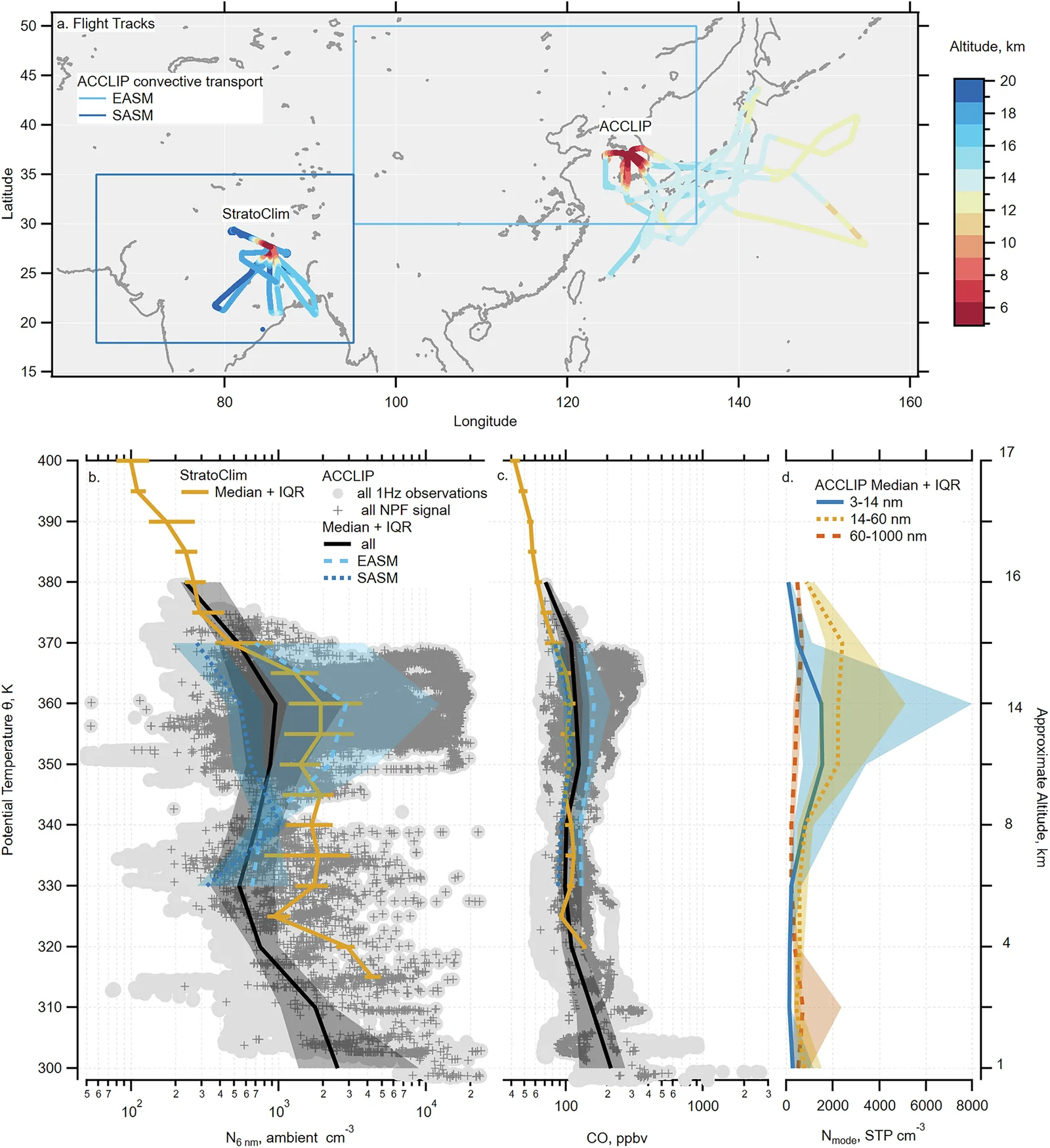
Observation locations and vertical structure of key observations. **a** Flight tracks of research flights used in this analysis from the Asian Summer Monsoon Chemical and Climate Impact Project (ACCLIP), Gulfstream-V (GV) aircraft and Stratospheric and upper tropospheric processes for better climate predictions (StratoClim), Geophysica aircraft coloured by flight altitude. Boxes show regions of airmass origin classified as East Asian Summer Monsoon (EASM, light blue), and South Asian Summer Monsoon (SASM, dark blue). The world map was made with Natural Earth vector map data (Country Borders, Coastlines, Physical Features): http://www.naturalearthdata.com. Total number concentration of particles with dry diameter above 6 nm (N_6_ nm) (**b**) and volume mixing ratio of CO (**c**) as a function of potential temperature (θ) at ambient temperature and pressure. Solid lines show median values of all 1 Hz data for ACCLIP (black) and StratoClim (orange) with interquartile range (IQR) shaded or in error bars. All 1 Hz data for ACCLIP are shown in light grey circles, and those data points indicating new particle formation (NPF, see methods) indicated in dark grey crosses. ACCLIP data are then separated into observed airmasses with 80% or greater association with East Asian Summer Monsoon (EASM) convection (light blue, dashed) or South Asian Summer Monsoon (SASM) convection (dark blue, dotted), with the lines showing the median, and IQR as shaded areas. Number concentrations by mode at standard temperature and pressure (STP) (**d**) separate the nucleation mode (3–14 nm dry diameter), Aitken mode (14–60 nm dry diameter) and accumulation mode (60 – 1000 nm dry diameter) from ACCLIP GV observations. Lines indicate median concentrations per altitude bin, shaded areas indicate the respective IQR. The approximate altitude corresponding to the observed potential temperatures is shown.

**Fig. 2:**
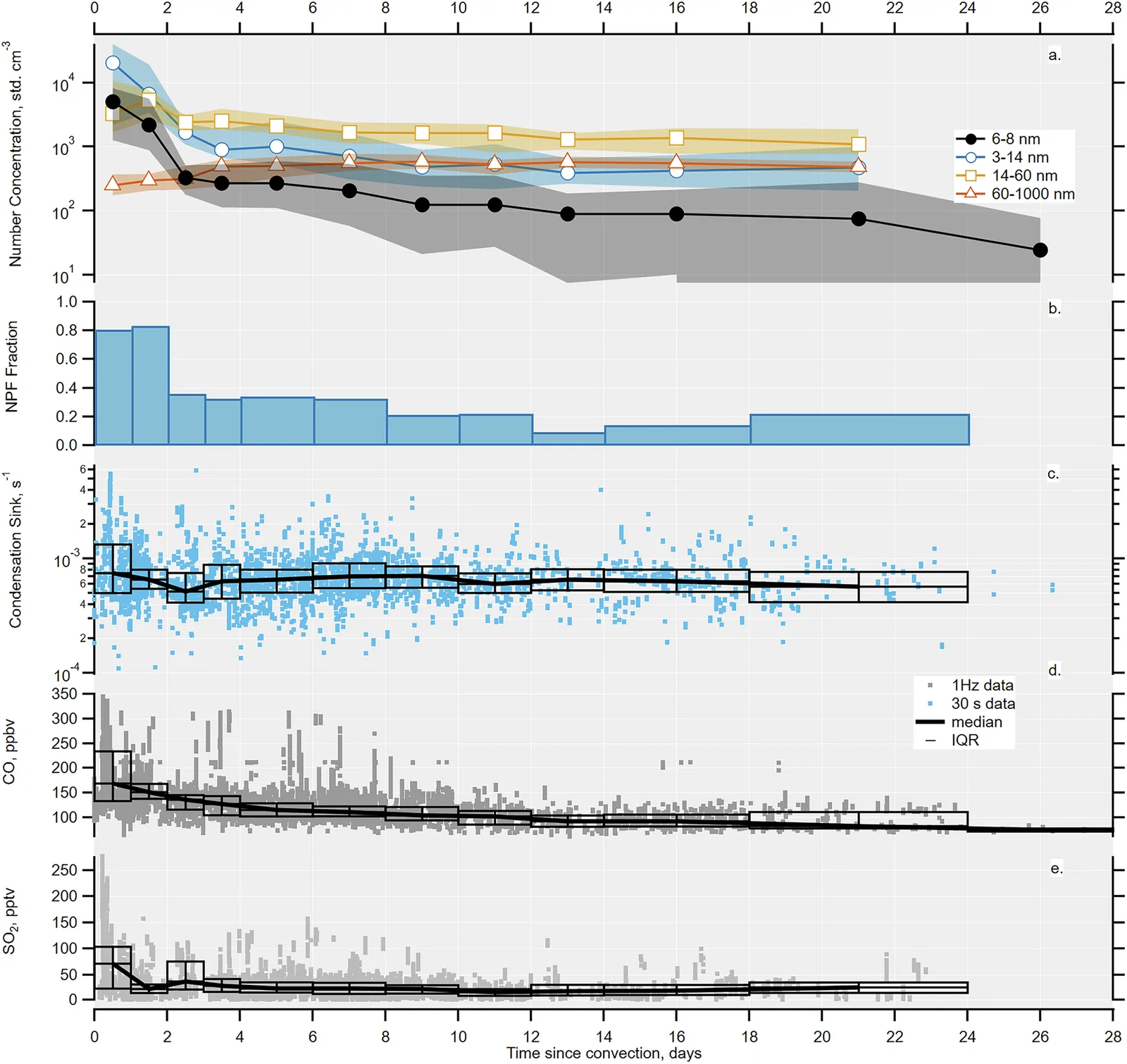
Observed aerosol and gas phase properties as a function of time since convection during the Asian Summer Monsoon Chemical and Climate Impact Project (ACCLIP). **a** Median number concentration in nucleation mode (3–14 nm, blue circles), Aitken mode (14–60 nm, orange squares), and accumulation mode (60–1000 nm, vermillion triangles) with interquartile range IQR (shaded) at standard temperature and pressure (std.) The number concentration in the first discrete NMASS channel (as opposed to the modes calculated from the inverted size distribution) between 6 and 8 nm is shown in black circles. **b** Fraction of data points indicating new particle formations (NPF, see methods) in blue bars. **c** Condensation sink (rate at which sulphuric acid condenses onto the existing aerosol population, indicating how much this population suppresses nucleation) calculated from the full observed size distribution. Blue dots are the 30 s data, black line and boxes show median and IQR, respectively. **d**, **e** Mixing ratio of carbon monoxide (CO) and sulphur dioxide (SO_2_) respectively, grey dots are 1 Hz data, black line and boxes show median and IQR, respectively.

Particle size distributions observed in ASM convective outflow during ACCLIP and StratoClim (Fig. 1a) show enhanced number concentrations peaking around 360 K potential temperature (Fig. 1b). The observations show similar spatial extent and number concentrations in both ACCLIP and StratoClim despite substantial interannual variation in the ASM and different sampling locations within the region (Supplementary Discussion section 1). The peak in number concentration coincides with peak convective outflow demonstrated in a maximum of observed carbon monoxide (CO) mixing ratios (Fig. 1c) in agreement with trajectory modelling^34,44^. This is due to high concentrations of particles in the nucleation and Aitken modes (defined here as 3–14 nm and 14–60 nm dry diameter, respectively) at high altitudes, concurrent with fresh convective outflow (Figs. 2a, 1d). Particles smaller than 14 nm have lifetimes of a few hours (Supplementary Discussion section 2), therefore these observations indicate recent NPF in the upper troposphere (UT).

We define NPF events as statistically significant number concentrations observed between 6 and 8 nm (see Methods). NPF observed during ACCLIP occurred in around 80% of measured airmasses within 2 days since convection, but rapidly becomes less frequent afterwards, with NPF events occurring in only 10–40% of observations sampled more than 2 days since convection (Fig. 2b). Similarly, peak concentrations from NPF observed during StratoClim mostly occurred within 3 days after convection^34^.

**Fig. 3:**
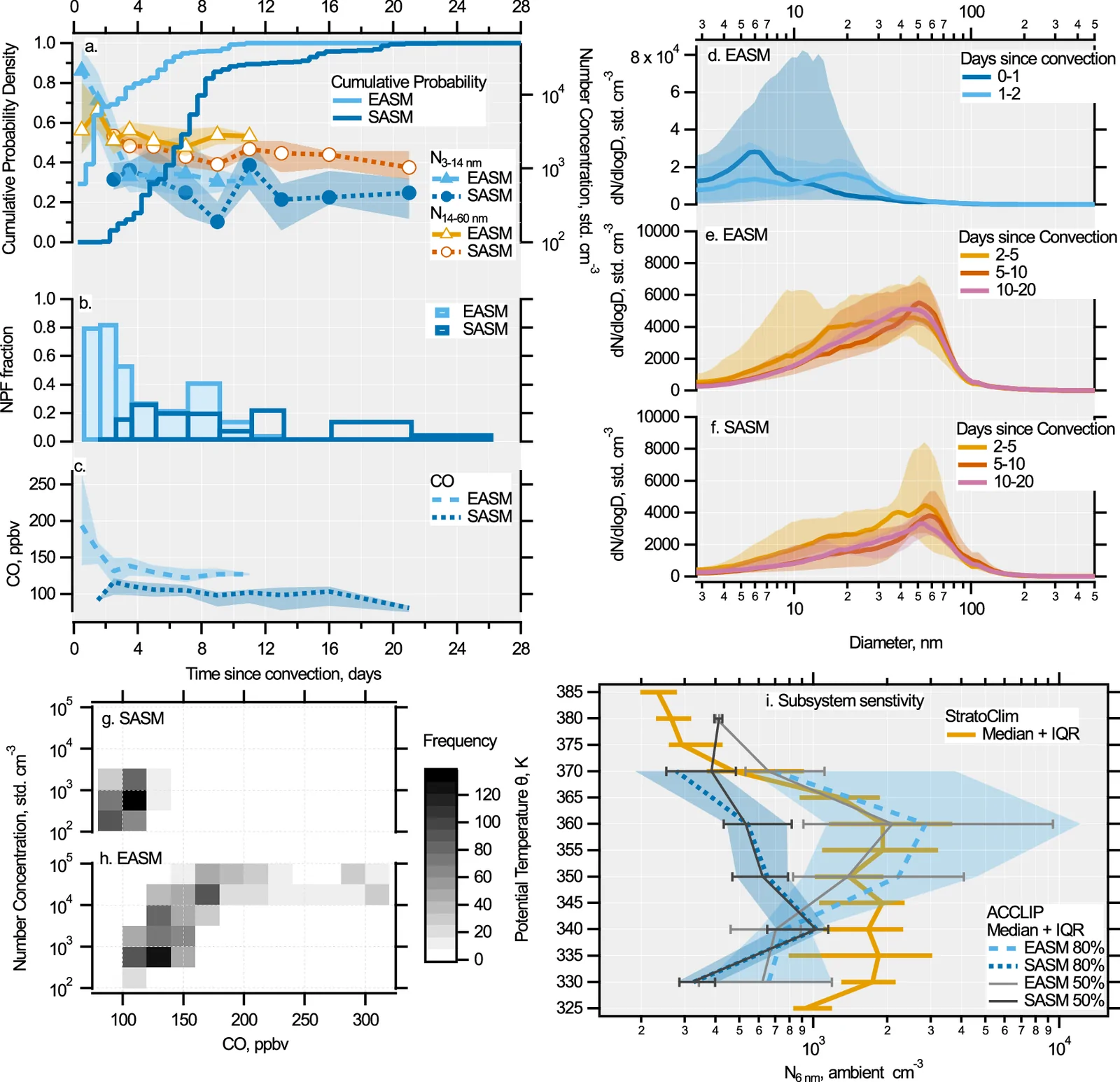
Monsoon subsystems. **a** Cumulative histograms of the median time since convection for each minute of flight associated with East Asian Summer Monsoon (EASM) or South Asian Summer Monsoon (SASM) (solid dark and light blue, respectively). Median of 30 s averaged number concentration of nucleation mode (3–14 nm, N_3–14 nm_, light blue dashed lien with triangles for the EASM, dark blue dotted line with circles for the SASM)and Aitken mode (14–60 nm, N_14-60 nm_, orange line with triangles for the EASM, vermillion dotted line with circles for the SASM) particles at standard temperature and pressure (std.) with interquartile range (IQR) shaded. **b** Fraction of 1 Hz data indicating new particle formation (NPF) in EASM airmasses (light blue) and SASM airmasses (dark blue). **c** Median CO mixing ratios observed in EASM airmasses (light blue, dashed), and SASM airmasses (dark blue, dotted) with IQR (shaded) binned by time since convection. **d-f** Median number size distributions (dN/dlogD) by time since convection for airmasses associated with EASM and SASM (IQR shaded). Joint histograms of the number concentration of nucleation mode aerosol at STP, and carbon monoxide (CO) mixing ratios in observations from the Asian Summer Monsoon Chemical and Climate Impacts Project (ACCLIP) related to SASM (**g**) and EASM (**h**). **i** Comparison of ambient number concentrations of particles greater than 6 nm between observations from the Stratospheric and upper tropospheric processes for better climate predictions (StratoClim) and ACCLIP EASM and SASM subsystems as a function of potential temperature (θ). ACCLIP EASM and SASM are shown for the subsystems assigned by 80% threshold of trajectories originating from each region (thick blue lines) and 50% threshold (thin grey lines).

ACCLIP observations sampled two subsystems of the ASM^8^: the East Asian Summer Monsoon (EASM), which has its centre of activity along the East Asia subtropical front^44^, and the South Asian Summer Monsoon (SASM), which has its centre of activity along the Monsoon Trough originating over Nepal and northern India and corresponds to the subsystem primarily observed during StratoClim^34^ (Fig. 1 and Supplementary Discussion section 1). Over 50% of EASM convective outflow sampled during ACCLIP was within 1 day after convection, while 50% of the SASM outflow was sampled more than 6 days after convection (Fig. 3a). ACCLIP observations show evidence of NPF in both EASM and SASM subsystems (Fig. 3). We see broad agreement between ACCLIP and StratoClim observations of the total particle number concentration in the UT, with the peak median number concentrations occurring around 360 K potential temperature (Fig. 1). When ACCLIP observations are split by contribution from EASM and SASM convection, we see closer agreement in median total number concentration between potential temperature of 350 and 370 K for EASM ACCLIP observations and total StratoClim (SASM) observations, despite the StratoClim observed CO mixing ratios being closer to those observed from SASM outflow on ACCLIP (Fig. 1b). The similarities in particle number concentrations in the first few days after convection indicate that NPF is occurring over the entire ASM convective region.

### Evidence for the role of pollution

**Fig. 4:**
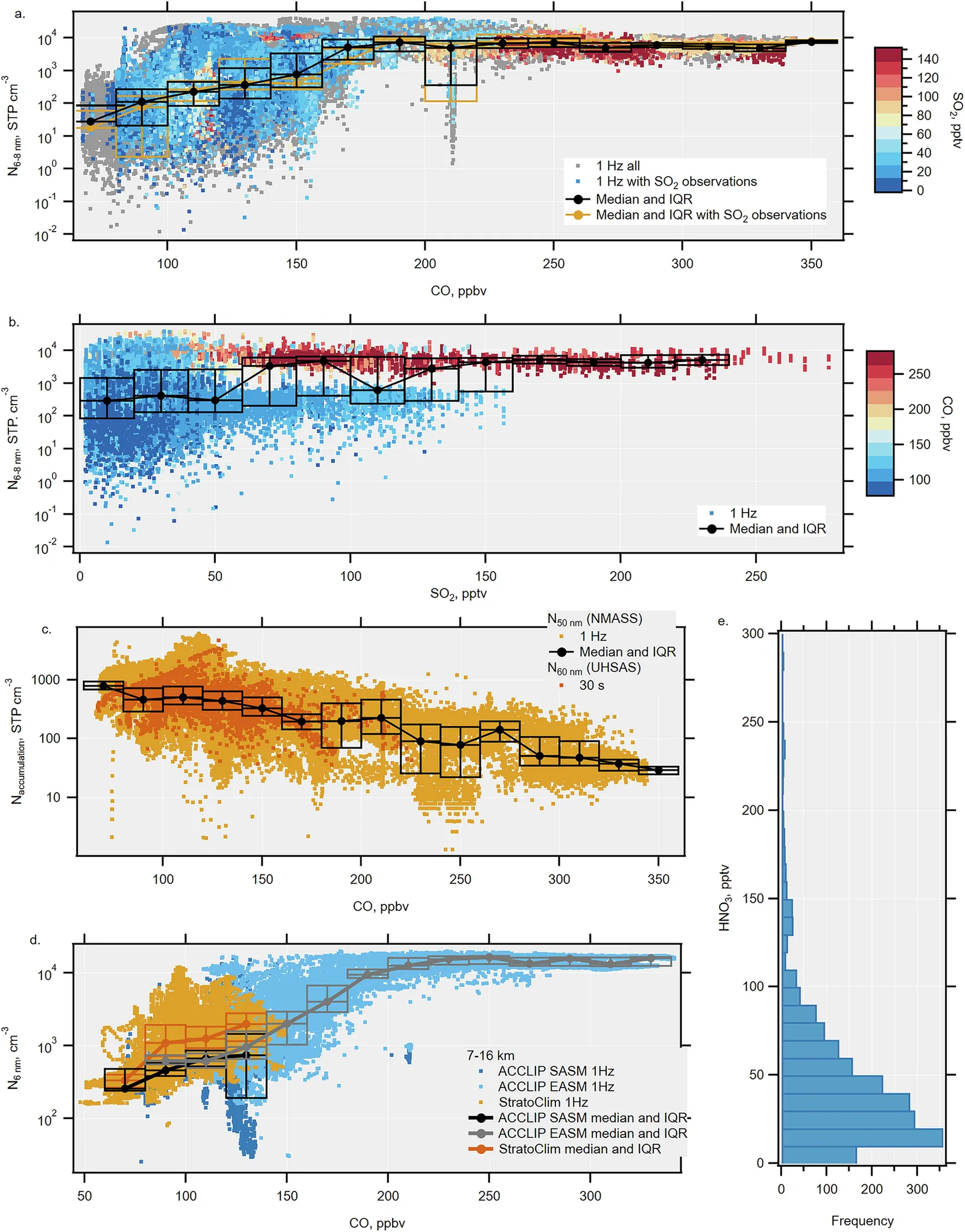
Small particle correlations with lofted pollutants. **a** Observations from the Asian Summer Monsoon Chemical and Climate Impacts Project (ACCLIP) of the correlation of aerosol number concentration between 6 and 8 nm (N_6-8 nm_) at standard temperature and pressure (STP) with carbon monoxide (CO) in grey dots, with those data where sulphur dioxide (SO_2_) was observed (only at the highest altitudes) coloured by SO_2_ mixing ratio, both 1 Hz. Black circles show the medians over each CO range (given by the horizontal error bars), and the inter-quartile range (IQR) is given by vertical error bars. Orange circles show the same as black circles, but only for data where SO_2_ was also observed. **b** SO_2_ ACCLIP observed correlation of N_6-8 nm_ with SO_2_ (coloured points), 1 Hz observations, while black circles show the medians over SO_2_ range as in panel (**a**). **c** Accumulation mode number concentration (N_accumulation_) at STP as a function of CO. Since the Ultra-High Sensitivity Aerosol Spectrometer (UHSAS) was not functional on Research Flight (RF) 08, where the highest CO volume mixing ratios were observed, the inverted number concentration above 60 nm (N_60nm_) at 30 s intervals including the UHSAS data and excluding RF08 is shown in vermillion dots, and the number concentration above 50 nm (N_50nm_) from the 1 Hz Nucleation Mode Aerosol Size Spectrometer (NMASS) 50 nm channel is shown in orange dots. The median and interquartile range (IQR) black boxes are calculated using N_50nm_. **d** 1 Hz observations together with the median and interquartile range (IQR) total number concentration above 6 nm (N_6 nm_) between 7 and 16 km as a function of CO from ACCLIP South Asian Summer Monsoon (SASM) and East Asian Summer Monsoon (EASM) (dark and light blue 1 Hz black and grey statistics respectively) and Stratospheric and upper tropospheric processes for better climate predictions (StratoClim) (orange 1 Hz, vermillion median and IQR). Statistics are only shown for CO bins with more than 5 min of data. **e** Histogram of nitric acid (HNO_3_) mixing ratio measured on ACCLIP. All HNO_3_ observations were made between 11.7 and 15.8 km altitude.

Evidence of pollution lofted from the boundary layer (BL) enhancing NPF in ASM outflow is given using carbon monoxide (CO) as a tracer for pollution (though it is not itself a condensable vapour for NPF^38,48^). ACCLIP and StratoClim observations show a strong correlation between observed particle number concentrations and CO mixing ratio (Fig. 4a). Particle number concentrations are more weakly correlated with SO_2_ (a precursor to H_2_SO_4_) than to CO (Fig. 4b), indicating the influence of condensable vapours beyond H_2_SO_4_.

The most likely nucleation mechanism is synergistic NH_3_-HNO_3_-H_2_SO_4_ nucleation, a fast UTLS nucleation and growth mechanism proposed from laboratory experiments and modelling^15,16^. SO_2_ and HNO_3_ were both observed in ASM outflow on ACCLIP with sufficiently high mixing ratios for this mechanism (Fig. 4), while high mixing ratios of ammonia have been observed previously in ASM convective outflow^35,36^. The parameterisation of the NH_3_-HNO_3_-H_2_SO_4_-nucleation rate is proportional to [H_2_SO_4_]^3^[HNO_3_]^2^[NH_3_]^4^^15^, showing that NH_3_ is the main driver of the nucleation rate. The stronger correlation with CO than with SO_2_ supports a surface origin NPF driver and is consistent with the assumption of the importance of NH_3_. While SO_2_ and HNO_3_ are also present in the stratosphere (e.g.,^49,50^), NH_3_ is derived primarily from surface anthropogenic sources^51,52^, and requires ASM convective lofting.

**Fig. 5:**
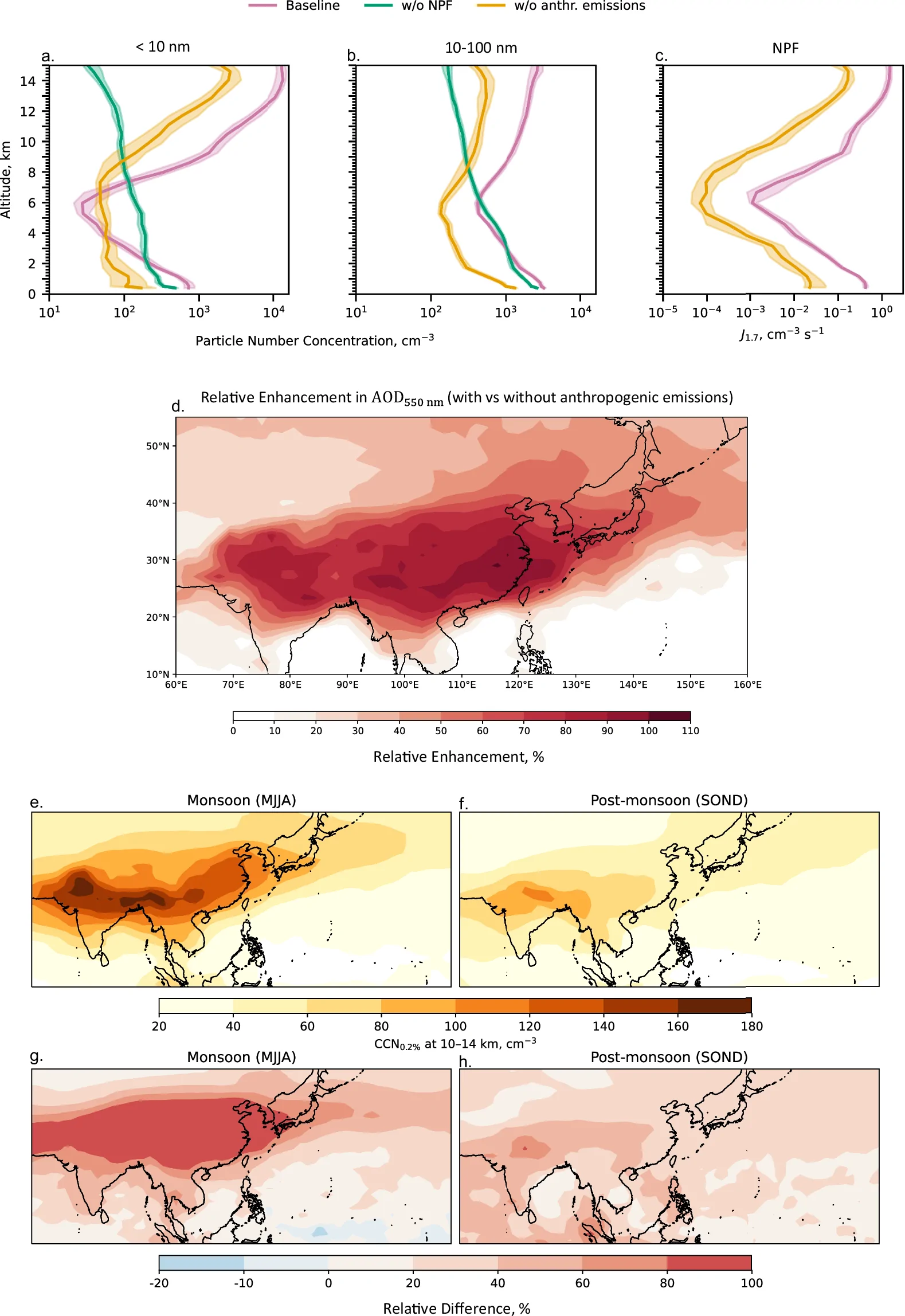
Targeted Earth system model sensitivity studies. ECHAM/MESSy Atmospheric Chemistry (EMAC) model sensitivity studies of the Asian Summer Monsoon Chemical Climate Impact Project (ACCLIP) campaign period (15 July–31 August 2022). **a**–**c** Vertical profiles of modelled particle number concentrations in the Asian monsoon region (10°–50°N, 60°–160°E) for a, particle diameter < 10 nm; (**b**), particle diameter 10–100 nm; and (**c**) new particle formation (NPF) rates at 1.7 nm diameter (*J*₁.₇). Results are shown for the baseline simulation with all emissions and nucleation active (purple), a sensitivity simulation with nucleation turned off (green), and a simulation without anthropogenic NOₓ, SO₂, and NH₃ emissions (orange). Lines indicate the median values and shaded regions the interquartile range (IQR) across the Asian monsoon region (10°–50°N, 60°–160°E). **d** Relative difference in upper-tropospheric aerosol optical depth at 550 nm (AOD_550_) showing the change that occurs when anthropogenic NOₓ, SO₂, and NH₃ emissions are included (relative to the simulation without anthropogenic emissions), averaged over 10–15 km. The simulated (EMAC) number concentration of cloud condensation nuclei (CCN) between 10 and 14 km altitude over the Asian Summer Monsoon (ASM) region is shown during the ASM (May, June, July, August – MJJA, **e**) and after the ASM (September, October, November, December – SOND, **f**), showing enhanced CCN at cloud outflow altitudes during the monsoon. The relative difference in CCN number concentrations over the same region and altitude range between the default model and the model runs without anthropogenic SO_2_, NO_x_, and NH_3_ emissions and shown for the monsoon period (**g**) and post-monsoon period (**h**), showing the CCN enhancement due to lofted gas phase pollutants. The world map was made with Natural Earth vector map data (Country Borders, Coastlines, Physical Features): http://www.naturalearthdata.com.

**Fig. 6:**
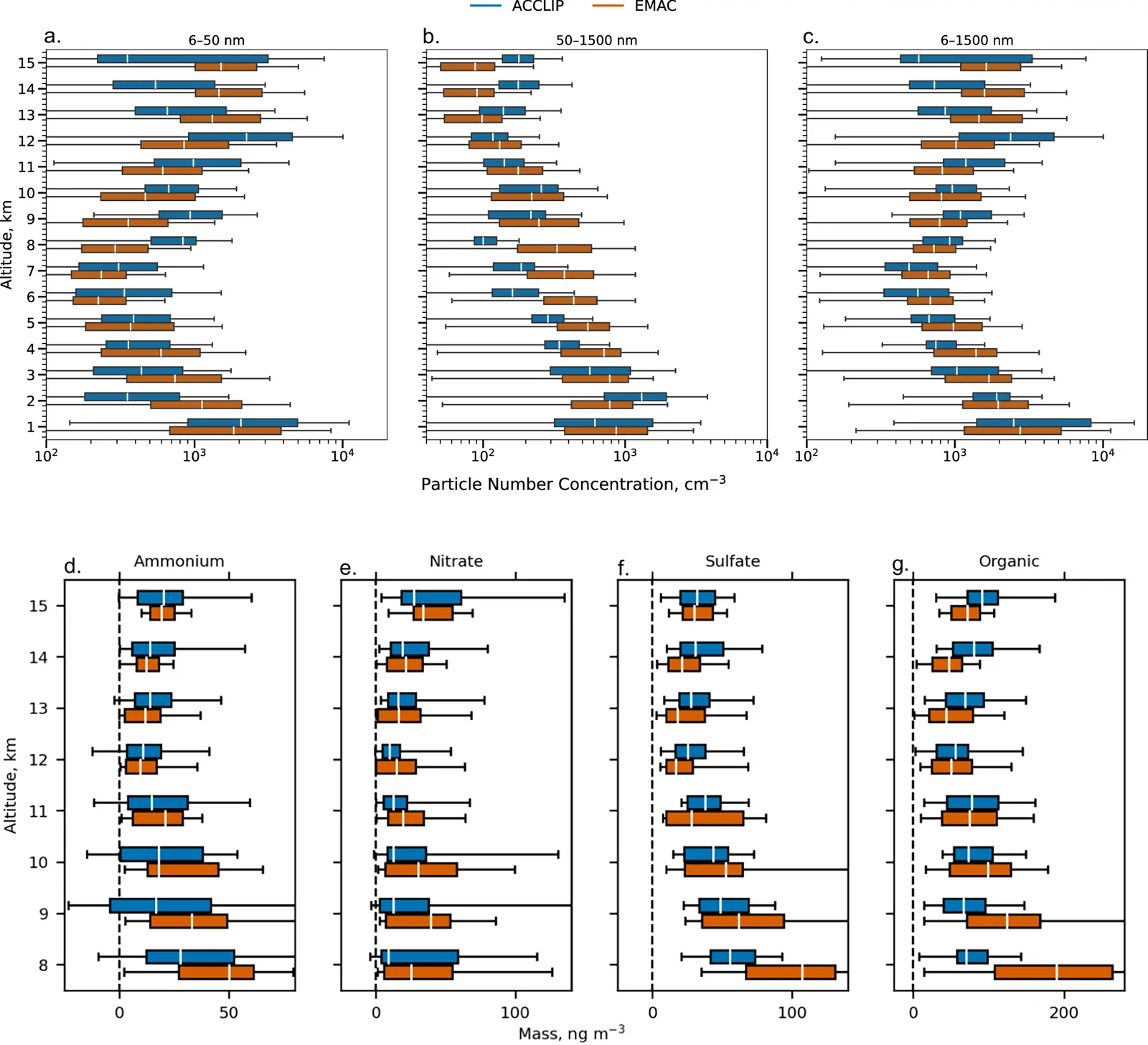
Comparison of targeted earth-system modelling and in situ observations. Comparison of ECHAM/MESSy Atmospheric Chemistry (EMAC) modelled particle number concentrations along the flight track (EMAC; vermillion) with aircraft observations from the Asian Summer Monsoon Chemical Climate Impact Project (ACCLIP) campaign (blue) across 1–15 km altitude range for 6-50 nm (**a**), 50-1500 nm (**b**) and 6–1500 nm particles (**c**). Boxplots compare the observed (ACCLIP) and simulated (EMAC) particle number concentrations in 1 km vertical bins. The vertical line inside each box marks the median, the box spans the 25th–75th percentiles, and the whiskers indicate the full data range. The mass concentration of ammonium (**d**), nitrate (**e**), sulphate (**f**) and organics (**g**) is similarly compared for particles between 120 and 3500 nm between EMAC simulations and ERICA observations.

Targeted simulations with the EMAC model (see Methods) predict substantial NPF activity in the upper troposphere over the ASM region consistent with the fast nucleation demonstrated above and vertical profiles of particle number concentrations consistent with those observed during ACCLIP (Figs. 5, 6). Sensitivity simulations further show that anthropogenic emissions of SO_2_ and NH_3_ strongly enhance NPF in the ASM UT (Fig. 5a–c).

**Fig. 7:**
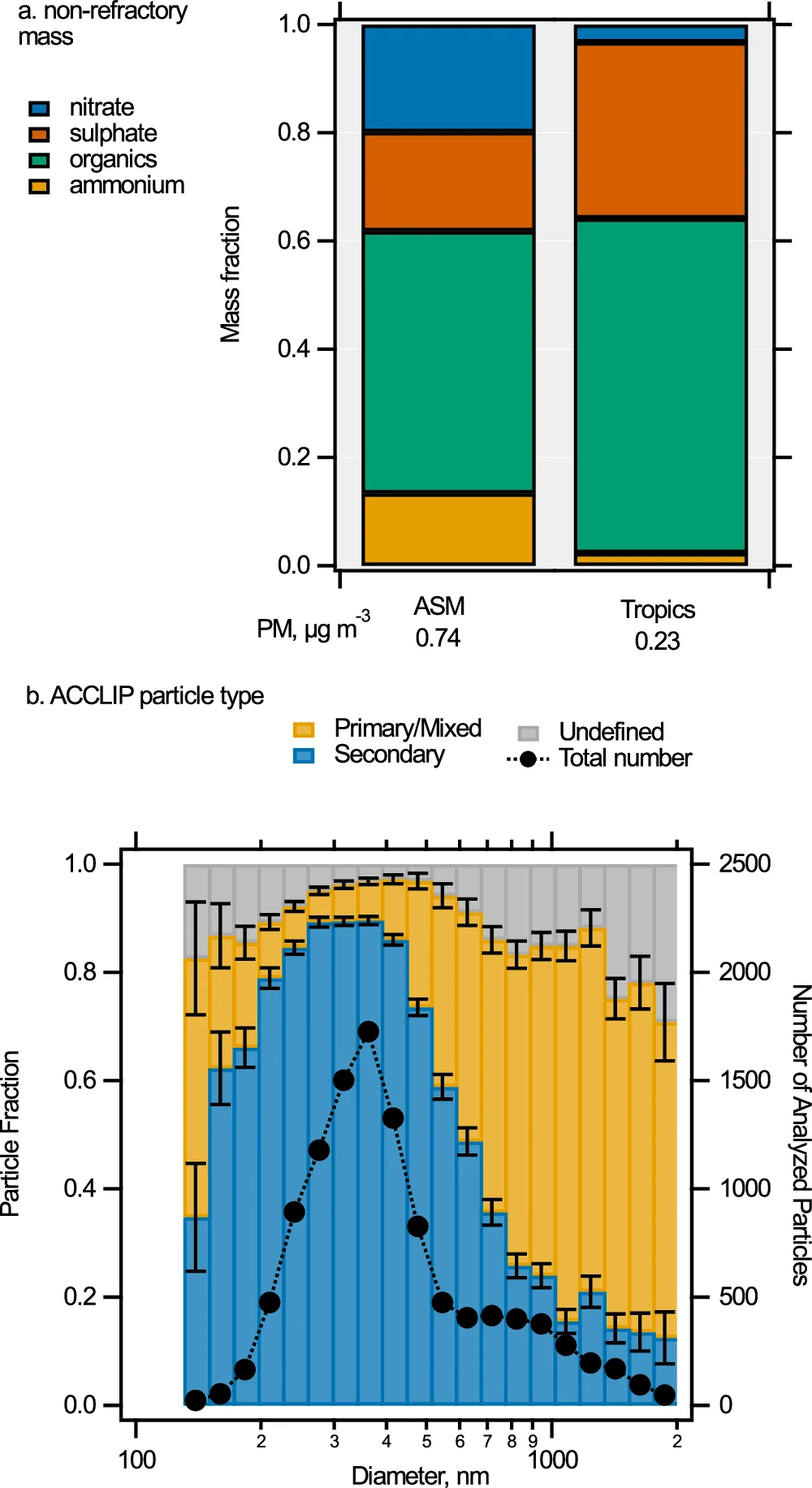
Composition and source of particles in Asian Summer Monsoon Convective Outflow. **a** Fraction of non-refractory aerosol measured by the Aerosol Mass Spectrometer (AMS) as nitrate (blue), sulphate (vermillion), organics (green) and ammonium (orange) in convective outflow in the Asan Summer Monsoon (ASM) from Asian Summer Monsoon Chemical Climate Impact Project (ACCLIP) GV and the remote tropical upper troposphere (Tropics) from Atmospheric Tomography Mission (ATom, flight tracks shown in Supplementary Fig. 4, tropical upper troposphere defined in “Methods”). **b** ERC Instrument for the Chemical composition of Aerosols laser ablation mass spectrometer ERICA-LAMS particle fraction (left) with number of particles sampled in each size bin (right, black circles and dotted line). The given uncertainties (black error bars) result from binomial statistics. For particle sizes between 200 and 500 nm, the observed size distribution is dominated by purely secondary particles (blue). For particles larger than 500 nm, we observed an increasing fraction of particles including primary compounds (orange, mainly potassium). The domination of secondary particles up to 500 nm highlights the growth of newly formed particles in the ASM outflow. Unclassified particles are shown in grey.

NH_3_-HNO_3_-H_2_SO_4_ nucleation results in ammonium-nitrate particles^14–16,47,53,54^. This is consistent with the high concentrations of ammonium and nitrate observed in accumulation mode particles on ACCLIP (Fig. 7, Fig. 6d, e), and previously on StratoClim^46,47^. EMAC simulations with NH_3_-HNO_3_-H_2_SO_4_ nucleation and growth produce particles in the ASM outflow that match the observed chemical composition well (Fig. 6d–g). This agreement indicates adequate representation of the precursor gases in EMAC and therefore supports the modelled nucleation mechanism. Organics are also abundant in the observed larger particles in ASM outflow (Figs. 7, 6g) and may be involved in nucleation and/or growth, similarly to in remote tropical convection^28^. However, so far, no mechanisms have been proposed for this. Future observations of the chemical composition of clusters and the smallest particles would be necessary to verify all nucleation mechanisms involved.

**Fig. 8:**
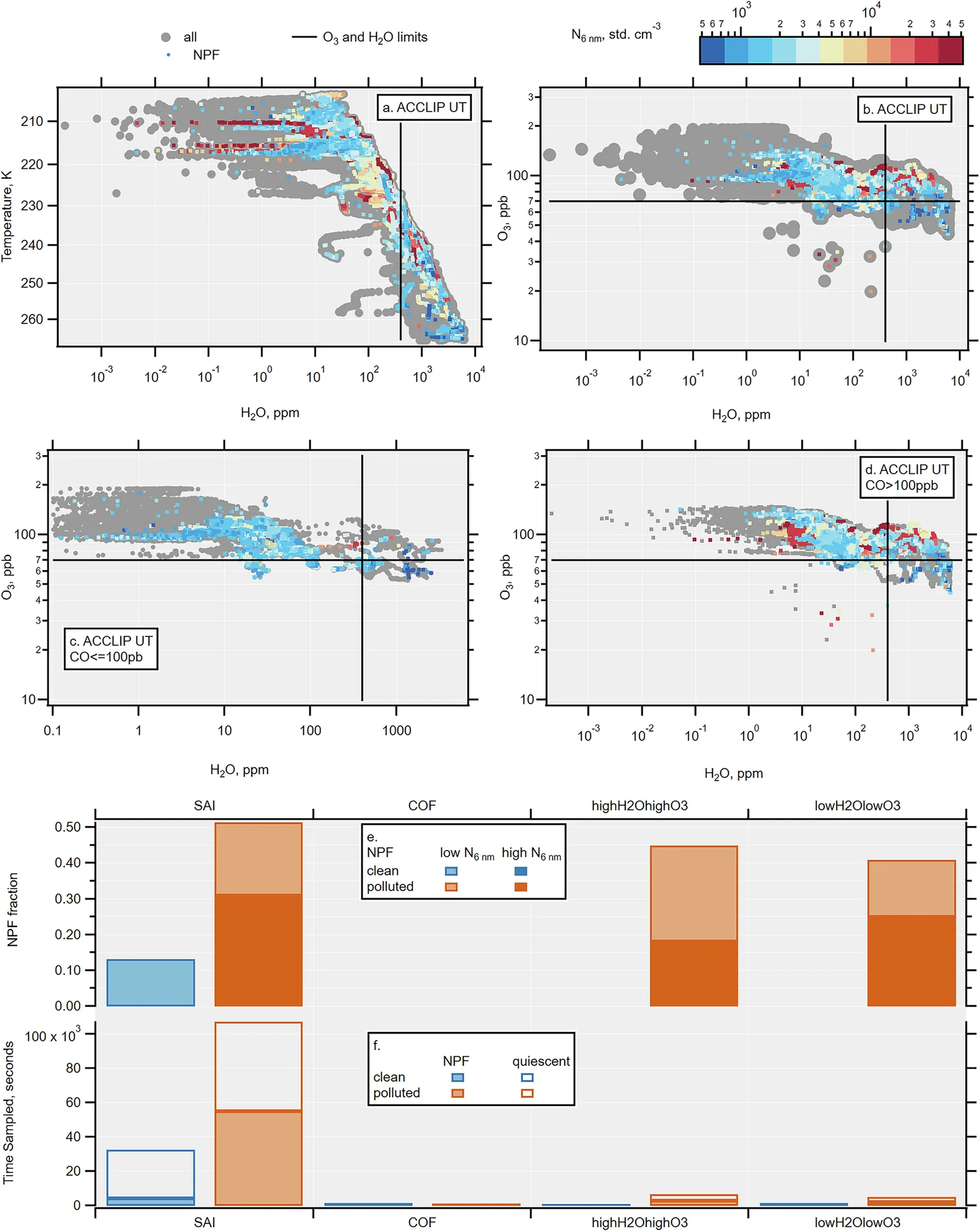
Influence of stratospheric air intrusions and temperature on new particle formation. Examining the influence of stratospheric air intrusions (SAI) and temperature on new particle formation (NPF) in Asian Summer Monsoon (ASM) with Asian Summer Monsoon Chemical Climate Impact Project (ACCLIP) observations. 4 upper troposphere (UT, above 7 km) regions defined by threshold mixing ratios of water vapour (H_2_O) and ozone (O_3_) and quantified as polluted or clean by threshold carbon monoxide (CO) mixing ratios. Threshold values are 100 ppbv CO, 400 ppmv H2O, and 70 ppbv O3. SAI is classified as high O3, low H2O, and COF as low O3, high H_2_O, following Zhang et al.^33^ (see “Methods”). 1 Hz data indicating NPF (coloured dots, with colours indicating the number concentration of particles greater than 6 nm at standard temperature and pressure), and all 1 Hz data (grey dots) from ACCLIP are examined. The black lines on panels (**a**–**d**) indicate threshold O_3_ and H_2_O mixing ratios. **a** temperature and H_2_O distribution. **b** O_3_ and H_2_O distributed for all data, (**c**) low CO data, and (**d**) high CO data. **e** The fraction of clean (blue, low CO) and polluted (vermillion, high CO) airmasses within each sampling region that showed statistically significant makers of NPF. Darker shading indicates NPF events with high number concentration (N_6_ > = 5000 cm-3 STP) and lighter shading indicates NPF events with low number concentrations (N_6_ < 5000 cm-3 STP). **f** the total number of 1 Hz observations made in clean (blue, low CO) and polluted (vermillion, high CO) airmasses in each sampling region, with shaded bars indicating observations with statistically significant NPF and unshaded bars indicating no statistically significant NPF.

Most of the airmasses sampled in ASM outflow on ACCLIP were somewhat influenced by stratospheric air intrusions (SAI, see “Methods”, Fig. 8), and the mixing between descending stratospheric air and lofted pollution may contribute to the high particle concentration observed in ASM outflow, as has been observed in less polluted convective regions^33^. Within SAI influenced airmasses, more frequent NPF and stronger NPF (high number concentrations) are associated with higher CO concentrations, supporting the conclusion that lofted pollution is the main NPF driver in ASM outflow Fig. 8e, f).

Alongside requiring sufficient supersaturation of condensable vapours, NPF is impeded by sinks for vapours and small particles as they condense onto or coagulate with other particles present (represented by the condensation sink). Both the ASM outflow condensation sink and SO_2_ mixing ratio show an initial maximum immediately after convection, coincident with the highest number concentrations of newly formed particles (Fig. 2). Box modelling indicates high supersaturation of condensable vapours driving efficient growth of newly formed particles, and high self-coagulation of the smallest particles (Supplementary Fig. 1).

### Growth to climate relevant sizes

**Fig. 9:**
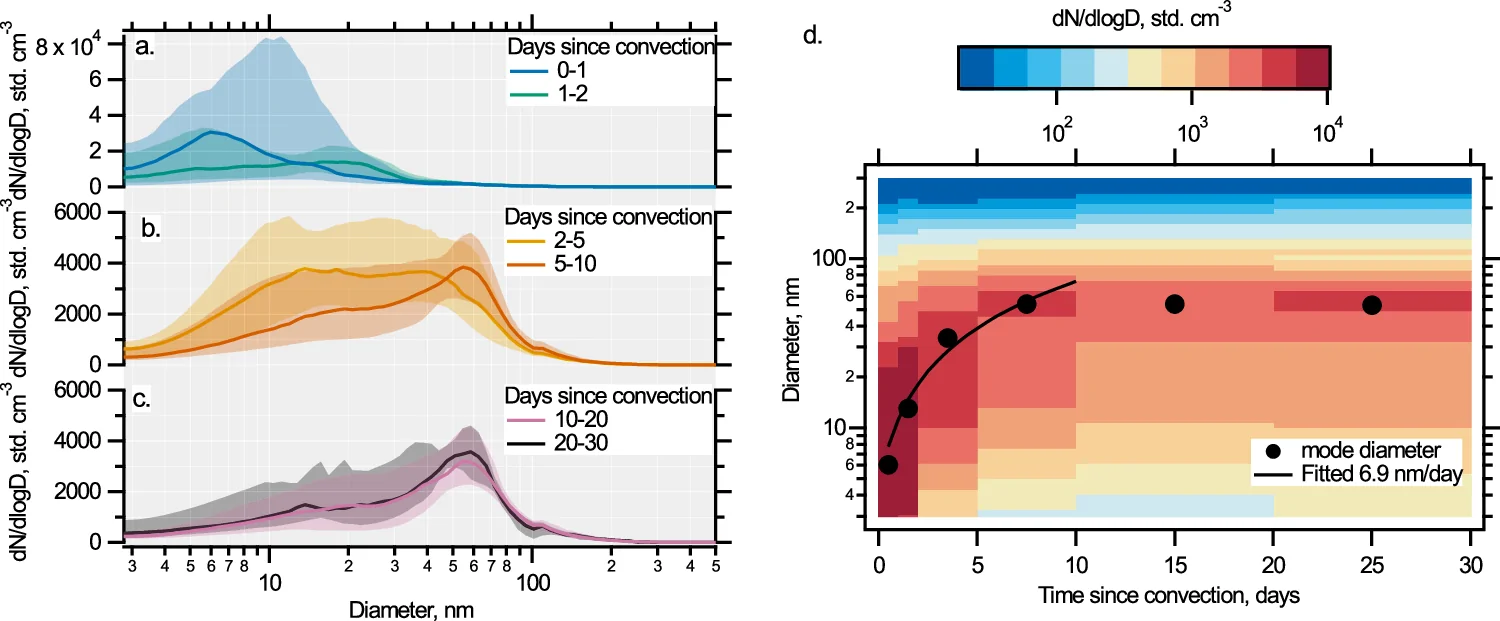
Evolution of the aerosol size distribution observed in Asian Summer Monsoon (ASM) convective outflow during the Asian Summer Monsoon Chemical Climate Impact Project (ACCLIP). **a**–**c** Number size distributions (dN/dlogD) from all research flights with median (lines) and interquartile range (IQR, shaded) at standard temperature and pressure (std.) are separated by median time since convection for the trajectory cluster associated with each 30 s flight track segment. Times since convection are 0-1 days (blue, a), 1-2 days (green, **a**), 2–5 days (orange, **b**), 5–10 days (vermillion, **b**), 10–20 days (purple, **c**) and 20–30 days (black, **c**). Note the y-scale for panels (**b**, **c**) is more than a factor 10 smaller than the scale for (**a**). **d** The median particle number size distribution (dN/dlogD) as a function of time since convection, with the mean diameter of the size distribution at each time interval, and a growth rate within the ASM region derived from the linear fit to the mode diameter over the first 10 days since convection.

ACCLIP observations provide evidence that particles formed in ASM convective outflow grow to diameters larger than 60 nm (Fig. 9), making interactions with clouds and radiation possible, and providing significant surface area to impact heterogeneous chemistry (Supplementary Fig. 2c, e, f). ACCLIP observations show a clear nucleation mode within 1 day of convection, the concentration of which then decreases rapidly with time since convection (Figs. 9, 2a). As time since convection increases, a pronounced Aitken mode evolves (1-2 days since convection), followed by an accumulation mode ( > 5 days since convection) (Figs. 4, 2a). The median and 25^th^ percentile of the Aitken mode are larger for 1-2 days since convection than 0-1 days since convection, indicating some growth from nucleation to Aitken mode, although the 75^th^ percentile decreases over this time. The trend indicated is that of particles forming very shortly after exiting the convective cloud and then growing over about a week to sizes where they have potential to act as CCN. While mixing of convective outflow and background airmasses could explain some size distribution changes with time since convection, it cannot account for the observed increase and then decrease of the Aitken mode number concentration. This picture does not change when dilution is taken in account (Supplementary Fig. 2a, b). Growth rates of around 7 nm/day are observed while airmasses remain in the ASM region (Fig. 9d). Once airmasses are transported away from this region, we would expect slower growth due to lower supersaturation of condensable vapours.

Very efficient wet removal of accumulation mode aerosols has been observed in monsoon convective transport^55^. The magnitude of the accumulation mode number concentration increases with time since convection (Fig. 9b) and is inversely correlated with CO (Fig. 4c), suggesting that many of these accumulation mode aerosols grow from the newly formed particles. This is supported by single particle mass spectrometer analysis, which shows that over half of the particles observed between 150 and 600 nm are purely secondary (Fig. 7), and by previous observations of increases in particle size with increasing potential temperature (corresponding with uplift by radiative heating within the anticyclone)^13^.

**Fig. 10:**
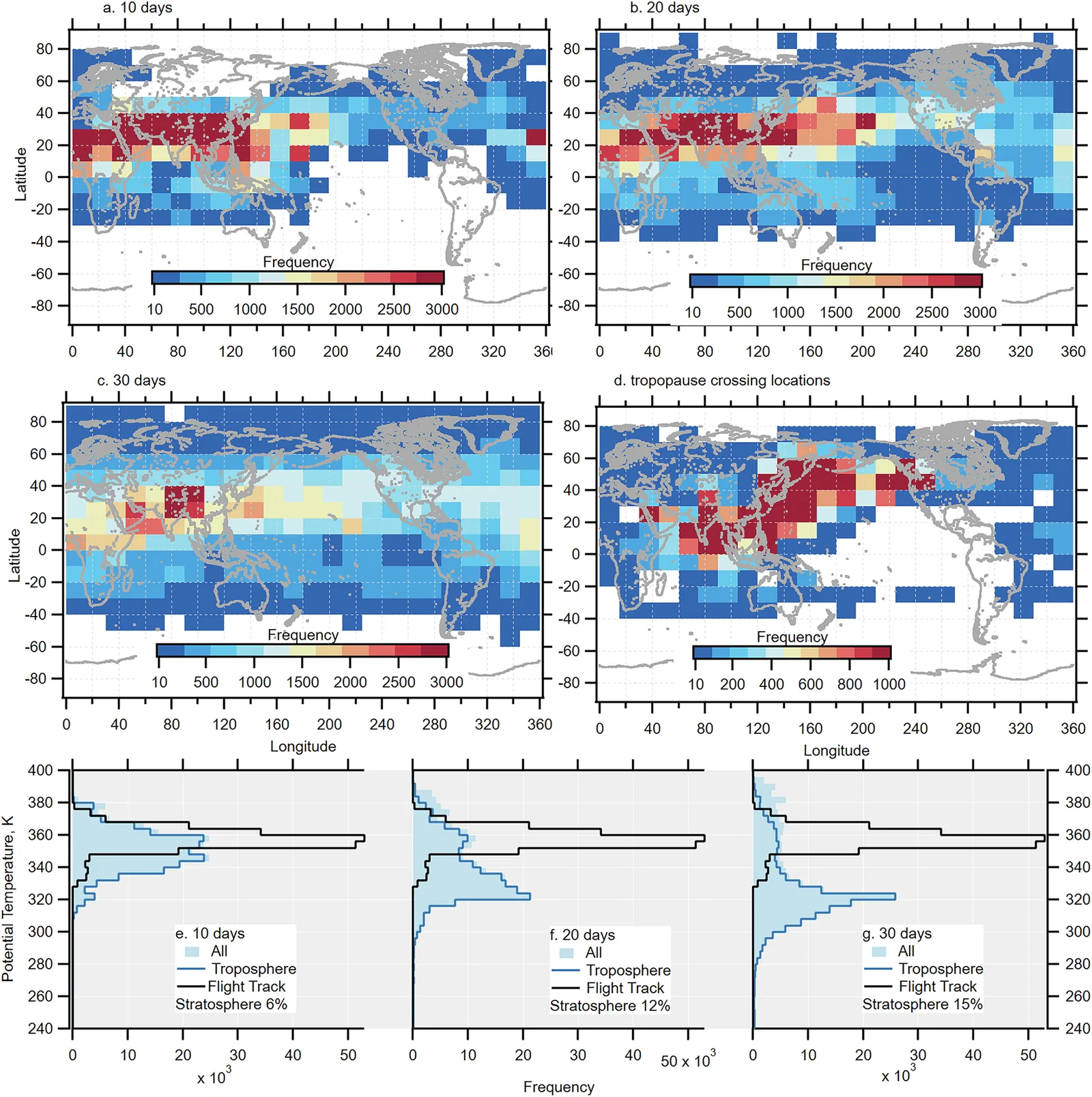
Forward Trajectory Modelling below the tropopause. Panels (**a**–**c**) show where the trajectories have reached 10, 20 and 30 days after observation, respectively. Panel (**d**) shows the location at which trajectories first cross the tropopause. Panels (**e**–**g**) show histograms of the potential temperature at 10, 20 and 30 days after observation, respectively, for parcels below the local tropopause (blue lines) and all parcels (blue shaded), along with the potential temperature distribution of flight track locations from where forward trajectories were launched (grey line). The world map was made with Natural Earth vector map data (Country Borders, Coastlines, Physical Features):http://www.naturalearthdata.com.

Forward trajectory modelling (see “Methods”) shows that within 30 days following observation, 15% of the airmasses sampled by the Gulfstream-V aircraft (GV) on ACCLIP above 330 K cross the tropopause into the stratosphere either locally within the anticyclone or further north in the region of the subtropical jet (Fig. 10) This local cross-tropopause transport is a significant source of ammonium nitrate and organic aerosol abundance in the stratosphere^47,56,57^. The majority of analysed air parcels descend through the troposphere and disperse on a hemispheric scale (Fig. 10 and Supplementary Discussion section 5). EMAC model sensitivity analysis (baseline vs. no anthropogenic NOx+SO₂ + NH₃ emissions) shows a systematic UT AOD and CCN enhancements over the ASM region (Fig. 5d–h), from anthropogenic-precursor influence, in agreement with Vernier et al.^9^. Over the ACCLIP domain, baseline vs. ‘no-anthropogenic’ yields + 60–100% UT (10–15 km) AOD (Fig. 5d), demonstrating the potential magnitude of UT aerosol enhancement from ASM lofted pollution. Therefore, while previous literature has focused heavily on the ASM as a source of particulate matter maintaining the ATAL, the effects of these particles on clouds and radiation in the troposphere must also be considered when evaluating the overall climate impacts of the ASM.

## Methods

### Research flights

We measured in-situ aerosol dry size distributions on the US National Science Foundation (NSF) Gulfstream-V (GV) aircraft in July and August 2022 during the Asian Summer Monsoon Chemical and Climate Impact Project (ACCLIP)^44,45^. The flights were planned to sample outflow from the ASM, and were based out of Osan Air Base, South Korea (Fig. 1a). All observations on the ACCLIP GV occurred in daylight hours, often close to solar noon (Supplementary Discussion section 4). Observations are used from research flights on 12^th^, 15^th^, 16^th^, 23^rd^, 25^th^, 26^th^, and 29^th^ August 2022, as those were the flights were all the relevant aerosol and cloud instrumentation were operational. The UHSAS was not operation on the 19^th^ August 2022 flight, but this flight sampled some of the largest enhancements of pollution measured during ACCLIP. Therefore, NMASS and gas phase data from 19^th^ August are included where possible, but full size distributions and accumulation mode concentrations do not include this flight.

We compare ACCLIP observations with those made in the ASM outflow in 2017 during StratoClim from the M-55 Geophysica stratospheric aircraft^13,34,46,47^, and with observations of UT convective outflow above the remote tropical Pacific and Atlantic Oceans from the NASA DC-8 aircraft from 2016–2018 on the Atmospheric Tomography Mission (ATom)^27,28,58^. We use data from ATom exclusively in the tropics (defined here as 30°S-30°N) from all four deployments (Supplementary Fig. 4).

### Size distribution observations

Size distribution measurements were made on ACCLIP using a Nucleation Mode Aerosol Size Spectrometer (NMASS)^59^ and an Ultra-High Sensitivity Aerosol Size Spectrometer (UHSAS, Droplet Measurement Technologies)^60^ operated inside the fuselage of the aircraft. The NMASS uses a battery of 5 condensation particle counters (CPCs) with different cut-off diameters between 3 and 60 nm to get cumulative particle number concentrations between each cut-off diameter and a maximum of 1500 nm (inlet transmission for particles larger than this is negligibly low). The NMASS was operated on ACCLIP with 5 bins over the 6–50 nm range (Supplementary Methods Section 5, Supplementary Fig. 5). The UHSAS is an optical particle counter which uses Mie scattering to measure the concentration of particles between 60 and 1500 nm. The UHSAS configuration is described in Kupc, et al.^60^.

For ACCLIP, the particle number size distribution, $N({D}_{p})$, is calculated from combined NMASS and UHSAS observations averaged to 30s-time intervals to reduce noise. First the UHSAS size distribution is averaged into channels of the same log diameter (${D}_{p}$) spacing as the NMASS, and the concentration, ${X}_{i}$ measured in each bin, i, is then given by Eq. (1),

1 $${X}_{i}=\int ^{\infty}_{0}N({D}_{p}){K}_{i}({D}_{p})d{D}_{p}$$

where ${K}_{i}({D}_{p})$ is the response function of channel i to a particle of diameter ${D}_{p}$. The inverse problem is solved using a version of the smoothed Twomey algorithm^61^, choosing one smooth, nonnegative solution that minimises the discrepancy between the predicted and actual instrument response. The inverted size distribution above 70 nm is then substituted with the full-resolution size distribution measured by the UHSAS. This is the same inversion procedure used previously for the NMASS-UHSAS combination^59,62^. Aerosol concentrations and size distributions are often presented at standard temperature and pressure (273.16 K and 1013 hPa, respectively).

During StratoClim, the 4-channel condensation nucleus counter COPAS (Condensation Particle counting System) was used^34,63^. Three COPAS channels operate with cut-off diameters of 6 nm, 10 nm, and 15 nm, a fourth channel (cut-off diameter: 10 nm) detects particles after heat exposure (270 °C) to quantify particulate residue or refractory particles (e.g., soot, mineral dust, metallic material, etc.). COPAS was installed in a non-pressurised part inside the aircraft fuselage with a length of ambient aerosol sample line of no more than ~ 1 m in total. Design and performance of the COPAS aerosol inlet system used on the M-55 Geophysica are described in Weigel, et al.^63^ and is assumed to aspire and transport particles of diameters < 1 µm at almost 100% efficiency. Aerosol size distributions in the diameter range of 65 nm <*d*_p_ < 1000 nm were measured during StratoClim with a wing-mounted UHSAS-A modified for operations at pressures as low as 45 hPa^13^. The modifications included a) the integration of a static pressure sensor in the detection cell of the UHSAS-A and b) a new pump system (double-head diaphragm pump) for maintaining constant system flows even at stratospheric air pressures. Mahnke et al.^13^ extended the StratoClim measurements of the UHSAS-A at 20 km altitude by an additional size bin (6 – 65 nm, based on COPAS data) and compared the resulting size distributions with those from balloon-borne observations in India and mid-latitudes in the USA^64,65^.

Size distribution data from ACCLIP have been filtered to only use clear-sky observations to avoid potential inlet artifacts in-cloud. ACCLIP cloud filtering was conducted using a combination of the in-situ liquid and ice water contents observed by the wing-mounted Cloud Droplet Probe (CDP, Droplet Measurement Technologies^66,67^) and Fast 2Dimensional-Stereo probe (F2D-S, Stratton Park Engineering Company Inc.^68^) to detect the 1 hz presence of cloud at aircraft altitude, adding a 3 s buffer to the aircraft entry and exit of cloud. StratoClim data has not been cloud filtered since aerosol size distribution observations on this mission were unaffected by artefacts in cloud and have been used to study nucleation-mode particles in ice-clouds^69^.

Indicators of new particle formation are calculated at 1 Hz time resolution using observations from the first two channels of the NMASS. These channels give concentrations of particles larger than a cut-off diameter of 6 and 8 nm on ACCLIP. NPF is indicated when the concentration in the first channel is larger than the concentration in the 2nd channel by 3 times more than can be accounted for by uncertainty due to flow variation and counting statistics, following the method from Williamson et al. 2019^27^. The fraction of data indicating NPF in a given location or during a given time period is calculated as the number of 1 Hz data points indicating NPF by this method, divided by the total number of 1 Hz data points in the selected period.

The condensation sink is a measure of how fast gas phase sulphuric acid is taken up by the existing aerosol population, indicating how much the existing aerosol population supresses nucleation. The condensation kernel is calculated from the inverted size distributions using a coagulation rate calculated with the Fuchs expression, following the method from Seinfeld and Pandis^70^ and assuming one of the particles is a sulphuric acid molecule (note that the accommodation coefficient, *α*, in equation (12) of ref. ^70^ should read 1/*α,* and our calculations reflect this. The sulphuric acid molecule mass is taken to be 98.079 g mol^−1^ ^71^, and the diameter is calculated from bulk properties^72^. Temperature effects on the probability distribution of monomer, dimers and trimers are neglected. All particles are assumed to have the density of water. Multiplying the condensation kernel by the number of particles in each size bin and summing over all sizes then gives the total condensation sink. The coagulation sink, which indicates how much any nucleated particles are prevented from growing by coagulating with the existing aerosol population, is closely related to the condensation sink and calculated in the same way, using two particles instead of one particle and one sulphuric acid molecule.

While observations on ACCLIP were not Lagrangian, we can learn about the evolution of particles within the ASM outflow by examining average characteristics of the observed size distributions as a function of time since convection. We estimate aerosol particle growth rate (GR) from the average size spectra in Fig. 9d following the methodology of Dal Maso et al.^73^. First, three-modal log-normal fits were calculated to the size distribution at each time step. GR was then calculated from a linear fit to the fitted log-normal modes. On the first day, we select the smallest modal diameter for the GR calculation. On subsequent days, we choose the second-smallest modal diameter to track the growth of the particles from the first day nucleation. This results in a GR of 6.9 nm / day.

### Aerosol mass spectrometry

The chemical composition of accumulation mode particles during ACCLIP was measured using the hybrid aerosol mass spectrometer ERICA (ERC Instrument for the Chemical composition of Aerosols)^74–76^. The first section of the instrument, the ERICA-LAMS (laser ablation mass spectrometer), applies the laser desorption ionisation technique to analyse the composition of individual particles in the size range between ~ 180 nm and 3.2 µm. The resulting bipolar mass spectra of individual particles were analysed for the presence of ion signals from primary and secondary compounds. If the mass spectrum contains ion signals from primary substances, such as potassium, metals, silicates, and elemental carbon, the particle is labelled as ‘Primary’. If only ion fragments from secondary compounds, such as nitrate, ammonium, sulphate, and secondary organics, are detected, the particle is classified as ‘Secondary’. Based on the vacuum-aerodynamic diameter derived from the particle velocity measured by the detection lasers, the particle fraction for both particle classes was determined for different size bins.

The second section of the instrument, the ERICA-AMS, operates on the flash vaporisation and electron impact ionisation technique. The quantitative measurements of particle ensembles provide mass concentrations of non-refractory material such as ammonium, nitrate, organics, and sulphate in the size range between ~ 120 nm and 3.5 µm.

The ions from electron impact ionisation were extracted by an extraction pulse at a frequency of 80 kHz. Consequently, the mass range extended to a maximum m/z of 280 during ACCLIP. In total, 32 000 extractions are averaged to one raw spectrum using the data acquisition card. One raw spectrum of ERICA-AMS represents an average of 0.4 s measurement time. One measurement cycle of ERICA-AMS consists of 10 s, resulting in 25 raw spectra. 11 raw spectra (4.4 s) are taken for an open and closed shutter position, respectively. The mass spectra recorded by ERICA-AMS were evaluated using the software Tofware 2.5.7 (TOFWERK AG, Switzerland) with adjusted settings to match the configurations for the ERICA-AMS data. The ACSM settings in Tofware 2.5.7 were adjusted to 2 − 12 (Total) and 14 − 24 (Background) to implement the correct shutter position in the evaluation software.

Based on ionisation efficiency (IE) calibration measurements with ammonium nitrate and sulfate during and after the campaign, an average IE value of 4415 ions pg^−1^ was determined for the entire campaign. For the relative ionisation efficiency (RIE), the resulting averages for ACCLIP were RIE(NH_4_) = 4.34 for ammonium and RIE(SO_4_) = 1.05 for sulfate.

The detection limit of observed mass concentrations was determined as 3 $\cdot \,\sigma$ ^47,77–79^, with σ representing the standard deviation of the original noise function. For ACCLIP, the average detection limits were 0.10 μg m^−3^ for nitrate, 0.14 μg m^−3^ for sulfate, 0.39 μg m^−3^ for ammonium and 0.40 μg m^−3^ for organics for data points of 10 s resolution. Further details on the data evaluation of ERICA can be found in Appel, et al.^47^ and Eppers, et al.^76^. Comparison is made to observations in the tropical upper troposphere from the Atmospheric Tomography Mission, ATom, where bulk non-refractory composition of particles between 20 and 700 nm diameter were measured by a high-resolution time-of-flight aerosol mass spectrometer (HR-ToF-AMS Aerodyne Inc., Billerica, USA)^80^. ATom tropical UT is identified as 30°N-30°S, above 7 km altitude.

### Refractory black carbon observations

The Single Particle Soot Photometer (SP2, Droplet Measurement Technologies, Longmont, CO^55^) uses laser-induced incandescence to detect individual rBC aerosol particles^81,82^ covering a size range of approximately 90–550 nm volume-equivalent diameter under ACCLIP operating conditions, assuming a void-free particle density of 1.8 g/cm³. To account for rBC particles in the accumulation mode that fall outside the SP2 detection range, a mass correction factor was applied by fitting a lognormal distribution to the measured size distribution. Details on data processing and uncertainty estimates (~ 25%) are provided in ref. ^55^. Observed rBC mass mixing ratios in the UTLS remained below 3 ng std. m^-^³, falling within the typical range of previously reported values for this atmospheric region^83^, and suggesting a radiative impact consistent with typical rBC loadings in the UTLS. rBC aerosol was found to be efficiently removed by wet scavenging during monsoonal convection^55^.

### CO Observations

The GV NSF NCAR carbon monoxide (CO) and nitrous oxide (N_2_O) instrument is a quantum cascade laser (QCL) direct absorbance spectrometer manufactured by Aerodyne Research, Inc.^84^. To improve accuracy, the free space optics are continuously purged with catalytically scrubbed dry nitrogen to reduce CO to less than 1ppbv. A multipoint laboratory calibration against several NOAA Global Monitoring Laboratory standards yields a transfer curve and links the airborne data to the World Meteorological Organisation Mole Fraction scale. The ACCLIP observations have a time resolution of 1 s, a precision of 0.3 ppbv, and an uncertainty of ± 1 ppbv.

The CO data for the StratoClim campaign are provided by the instrument COLD2 (Carbon Oxide Laser Detector 2^85^), a quantum cascade laser spectrometer, measuring in direct absorption CO and N_2_O. Time resolution is 1 s, accuracy 3 %, sensitivity 1-2 ppb.

### Sulphur dioxide and nitric acid

Sulphur dioxide (SO_2_) and nitric acid (HNO_3_) were measured on the GV during ACCLIP by the Georgia Tech Chemical Ionisation Mass Spectrometer (GT-CIMS) at high altitudes. SO_2_ was calibrated frequently in flight by the addition of an isotopic SO_2_ standard. The sensitivity of HNO_3_ was examined relative to SO_2_ in post-mission calibrations conducted under dew point conditions representative of those observed during ACCLIP. Background measurements were performed periodically by drawing ambient air through a scrubber containing glass wool impregnated with sodium carbonate. These measurements were made at 5 s intervals with an estimated accuracy of 10% and 50% for SO_2_ and HNO_3_, respectively^86,87^. For this analysis, they are expanded to have the same data value at 1 Hz within each 5 s measurement period, and no value outside those periods. GT-CIMS was not operational during ACCLIP 12^th^ and 15^th^ August.

### Meteorological observations

ACCLIP GV global positioning, meteorological and thermodynamic variables are measured and calculated by NCAR Research Aviation Facility instrumentation^88^ and recorded in the field data archive. StratoClim meteorological data were collected by sensors in the nose boom of the M55 Geophysica aircraft, they were transmitted with position, time and flight attitude datas are recorded by individual instruments^13,89^.

### Convective transport diagnostics

We apply a convective transport diagnostic developed for ACCLIP to estimate the locations and times of convective transport which contributed to the measurements^8,90^. At each 60 s interval along the ACCLIP flight tracks, a 75-member “cluster” of backward trajectories is initiated using the TRAJ3D model^91–93^ and is run for up to 30 days using winds from ERA5 reanalysis^94^. Trajectories are traced through a field of convective cloud top altitudes derived from satellite observations^95^ until their first (i.e., most recent) encounter with a co-located convective cloud which is taller than the trajectory height. This location and time are then “assigned” to the airborne observations taken at the time of the trajectory initiation. All observations within 60 s of a given trajectory launch are assigned to the results from the convective transport diagnostic.

The convective transport diagnostic has revealed that the contribution of convection to ACCLIP sampling was primarily from two ASM sub-systems: the East Asian Summer Monsoon (EASM) and the South Asian Summer Monsoon (SASM). We classify an airborne sample as associated with these sub-systems if at least 80% of the associated trajectory cluster (i.e., at least 60 trajectories) are assigned to convective transport in these regions, using the same location criteria provided by Smith, et al.^8^. A sensitivity study shows that choosing 50 or 80 % of the trajectory cluster to determine the ASM subsystem does not alter the conclusions (Fig. 3i).

### Forward trajectory modelling

To understand the climate impacts of the particles that form and grow in ASM outflow, we use forward trajectory modelling to simulate the location of air parcels for 30 days following observations. These forward trajectories are launched every 60 s along the GV flight tracks for samples above 330 K potential temperature. Trajectories are calculated using horizontal winds and diabatic heating rates from ERA5 reanalysis^94^ in the TRAJ3D model^91–93^. To account for trajectory uncertainties, at each 60-second initiation along the flight track, we calculate a cluster of 75 trajectories centred on the flight track (5 × 5 x 3 trajectories in a regular 0.1° longitude x 0.1° latitude x 0.04 K grid) and calculate various statistics based on all trajectories. We further calculate each forward trajectory’s first tropopause “crossing” using a lapse rate tropopause product based on ERA5 reanalysis^96^.

### Stratospheric air intrusions and temperature effects

The potential role of stratospheric air intrusions (SAI) to increase NPF is explored following Zhang et al.^33^, which defines SAI in less polluted conditions as low water vapour (10–400 ppm), low CO ( < 100 ppb) and high ozone (70–100 ppb), and convective outflow (COF) as high water vapour (300–4000 ppm) and low ozone (30–70 ppb), or high CO ( > 100 ppb). Since ASM observations are strongly influenced by anthropogenic emissions, we neglect any prior CO limits and examine ASM outflow (altitudes above 7 km) as a function of H_2_O and O_3_, with limits of 400 ppm and 70 ppb respectively (Fig. 8). SAI influenced airmasses (high O_3_ and low H_2_O) dominate the ACCLIP observations, while pure convective outflow (COF) was rarely observed at altitudes above 7 km (Fig. 8f). Within SAI influenced airmasses, NPF occurred in over 50% of polluted airmasses (CO > 100 ppb) and 14% pf clean airmasses (CO < 100 ppb) (Fig. 8f). When we examine strong NPF events (N6 > 500 cm^−3^) (Fig. 8e) this is even more pronounced. In non-SAI airmasses, non-polluted air was rarely observed, but NPF and strong-NPF frequently occurred in these polluted airmasses (Fig. 8e, f). These observations show that SAI may influence ASM outflow NPF but cannot alone explain the observed frequency and strength of NPF. Pollution is the main driver. Temperature profiles of the ACCLIP UT observations (Fig. 8a) show that NPF, and especially that resulting in the highest number concentrations, is not occurring at the lowest temperatures.

### EMAC Model configuration

We use numerical simulations from the ECHAM/MESSy Atmospheric Chemistry (EMAC) model, which couples the ECHAM5 general circulation model^97^ with the MESSy2^98^ framework to simulate global meteorology, trace gases, and aerosols.

The model setup used in this study follows Xenofontos et al.^16,37^, using an identical EMAC configuration and CERN CLOUD-derived NPF parameterisations. Simulations are performed with EMAC (ECHAM5 v5.3.02, MESSy v2.55.2) at T63L90 resolution ( ~ 1.9° × 1.9°, 90 hybrid levels to ~ 80 km) for January–December 2022 using a 10 min time step and hourly output. Large-scale dynamics are nudged toward ERA5 reanalysis to ensure realistic meteorology and transport. EMAC submodels employed include GMXe (aerosol microphysics, using only 4 size modes in contrast to the box modelling), NAN (new particle formation), IONS (ion-pair production from galactic rays and radon decay), MECCA (gas-phase chemistry), JVAL (photolysis), DRYDEP (dry deposition), and AEROPT (optical properties). Convection is parameterised using the Tiedtke scheme with Nordeng closure, and lightning NOₓ production follows the scheme of Grewe. Anthropogenic emissions are derived from the CEDS inventory^99^. SO₂ oxidation forms sulfate aerosol, while lightning-produced NO drives UT NOₓ chemistry. Detailed description of the model can be found in Xenofontos et al.^16^.

For this study, we conduct three simulations with identical model configurations: (1) a baseline simulation; (2) a simulation without NPF; and (3) a simulation with anthropogenic NOₓ, SO₂, and NH₃ emissions turned off globally. Model data are sampled at the model grid boxes along the actual flight tracks, using the specific flight dates and times, to ensure the closest possible correspondence with the observed measurements. Model data are compared to all flights during ACCLIP.

## Supplementary information

Supplementaty Information

Transparent Peer Review file

## Data Availability

Observational data and trajectory modelling related to ACCLIP used in this study are available from the campaign data archive (NSF NCAR EOL,https://data.eol.ucar.edu/project/ACCLIP). Observations from StratoClim are available from the HALO database https://halo-db.pa.op.dlr.de/mission/101 (last access: June 2025). The full observational dataset relating to ATom is given in Wofsy, et al.^100^. This may also be accessed at https://espoarchive.nasa.gov/archive/browse/atom (last access: April 2025). Outputs from GMXe modelling are available at https://doi.org/10.5281/zenodo.20326825.

## Code availability

The Modular Earth Submodel System (MESSy, https://zenodo.org/communities/messy) is continuously further developed and applied by a consortium of institutions. The usage of MESSy and access to the source code is licensed to all affiliates of institutions which are members of the MESSy Consortium. Institutions can become members of the MESSy Consortium by signing the MESSy Memorandum of Understanding. More information can be found on the MESSy Consortium Website (http://www.messy-interface.org). Code for the GMXe model is available at https://doi.org/10.5281/zenodo.20326825.
