# Supplementary material for: Upper troposphere aerosol formation enhanced by monsoon lofted pollution
Source: Nat Commun. 2026 Aug 12;17:9672. doi: 10.1038/s41467-026-75073-x (PMC13558689; doi:10.1038/s41467-026-75073-x)
Supplement: Supplementary file 1 [file 41467_2026_75073_MOESM1_ESM.pdf]

Supplementary of

Upper Troposphere Aerosol Formation Enhanced by Monsoon Lofted Pollution

Christina J Williamson[1,2], Stephan Borrmann[3], Philipp Brauner[3], Charles A Brock[4], Teresa Campos[5], Theodoros Christoudias[6], Francesco D'Amato[7], Antonis Dragoneas[3], Oliver Eppers[3], Shawn Honomichl[5], Matthias Kohl[3], Franziska Köllner[8,3], Agnieszka Kupc[9], Young Ro Lee[10], Ming Lyu[11,4], Georgia Michailoudi[11,4], Laura L Pan[5], Anne E Perring[11,12], Andrea Pozzer[3,6], Dylan Simone[11], Warren P Smith[5], Holger Tost[8], Rei Ueyama[13], Kirk Ullmann[5], Ville Vakkari[1,14], Silvia Viciani[7], Aki Virkkula[1], Ralf Weigel[8], Sarah Woods[5], Christos Xenofontos[6]

[1]Finnish Meteorological Institute, Helsinki, 00101, Finland
[2]Institute for Atmospheric and Earth System Research/Physics, University of Helsinki, Helsinki, 00014, Finland
[3]Max Planck Institute for Chemistry, Mainz, 55128, Germany
[4]Chemical Sciences Laboratory, National Oceanic and Atmospheric Administration, Boulder, CO 80305, USA
[5]Atmospheric Chemistry Observations & Modeling Laboratory, NSF National Center for Atmospheric Research, Boulder, CO 80301, USA
[6]Climate and Atmosphere Research Center, The Cyprus Institute, Nicosia 1645, Cyprus
[7]National Research Council—National Institute of Optics (CNR-INO), Sesto Fiorentino, Italy
[8]Johannes Gutenberg University, Institute for Atmospheric Physics, Mainz, 55128, Germany
[9]Aerosol Physics and Environmental Physics, Faculty of Physics, University of Vienna, Vienna, 1090, Austria
[10]School of Earth and Atmospheric Sciences, Georgia Institute of Technology, Atlanta, GA, 30332, USA
[11]Cooperative Institute for Research in Environmental Science, University of Colorado, Boulder, CO 80309, USA
[12]Department of Chemistry, Colgate University, Hamilton, NY 13346, USA
[13]Earth Science Division, NASA Ames Research Center, Moffett Field, CA 94035, USA
[14]Atmospheric Chemistry Research Group, Chemical Resource Beneficiation, North-West University, Potchefstroom, South Africa

Supplementary Discussion

SD1. ASM subsystems

In past studies, the convective transport to the ASM anticyclone was found to be primarily characterized by convective uplift from the southern flank of the Tibetan Plateau (e.g., [1-3]). This subsystem of the ASM is also known as the South Asian Summer Monsoon (SASM). This subsystem was found to be the dominant vertical transport pathway during the StratoClim mission 2017[4]. In 2022, however, the Asian Summer Monsoon (ASM) region experienced anomalous large-scale dynamical conditions during late summer, leading to extreme weather events in East Asia, such as the record-breaking rainfall in early August[5,6]. These conditions were associated with enhanced convective activity over parts of northern China and the Yellow Sea, which were linked to a northward migration of the East Asian Subtropical Front[7]. This migration was induced by the triple-dip La Niña event of 2022[7,8]. During the ACCLIP campaign, the sampling location over the western Pacific region coincided with deep convection over East Asia in August 2022, facilitating the sampling of East Asian Summer Monsoon (EASM) transport in addition to SASM air. The chemical characteristics of the ASM anticyclone in the UTLS is expected to differ between both sub-systems due to different environmental forcings and emissions. Indeed, Smith, et al. [9] found a higher abundance of pollutants in the UTLS from EASM convection compared to convection from the SASM.

A recent study by Eppers, et al. [10] compared the chemical characteristics of aerosol particles from SASM and EASM during ACCLIP using the hybrid aerosol mass spectrometer ERICA. Their findings showed that the mass fractions of sulfate and organic compounds were higher in EASM air relative to SASM air, while ammonium nitrate was lower. The authors hypothesized that elevated SO_2 and volatile organic compound (VOC) emissions in eastern China could explain these compositional differences. This hypothesis is supported by SO_2 observations from ACCLIP, which indicated higher SO_2 levels in air masses influenced by EASM convection compared to those influenced by SASM[9].

However, in absolute terms, the mass concentrations of aerosol particles measured by ERICA were lower in the UT for EASM compared to SASM[10]. This discrepancy was attributed to the relatively fresh nature of the EASM convective outflow sampled during ACCLIP. Specifically, the newly formed particles in the UT may not have had sufficient time to grow into the detectable size range of ERICA in the accumulation mode.

Airmasses sampled on ACCLIP from both EASM and SASM convective outflow show elevated concentrations of nucleation (3-14 nm) and Aitken (14-60 nm) mode particles, with the highest concentrations generally appearing in more recently lofted air (Fig. 3a). While these concentrations were fairly similar for similar times since convection, they were generally higher in EASM airmasses (Fig. 3a). The EASM outflow observed on ACCLIP was more polluted than SASM outflow, as shown by the higher CO mixing ratios, even at comparable times since convection (Fig. 3c)[9]. EASM outflow shows a positive correlation between CO mixing ratio and nucleation mode particle concentration and a clear particle growth trend (Fig.3). Neither of these are clear in the ACCLIP SASM observations, though a lack of observations in the first few days since convection is at least partly responsible for this. This suggests that, while NPF occurs in both subsystems, it is strongest in the EASM subsystem due to higher levels of pollution.

A very high frequency of NPF events is seen only in EASM outflow since ACCLIP did not observe SASM outflow within the first few days since convection[9], but both subsystems show similar NPF frequency after three days (3b). EASM particle number size distributions show the same time

evolution as all data taken together (Fig. 3d, e). SASM particle number size distributions show growth from the Aitken to accumulation mode from 2-10 days since convection, but not enough observations were made to determine the trend within two days of convection (Fig. 3a f).

SD2. Lifetime and growth of newly formed particles

The lifetime of newly formed particles in ASM outflow is estimated using the box-model (GMXe, see Supplementary Methods). Modelled lifetimes of nucleation mode aerosols (defined here as diameters 3-14 nm) are 18 - 22 h 1 day after convection, 27-47 h 2 days after convection, and peak at 30-60 h nearly 3 days after convection (Supplementary Fig. 1c). The modelled lifetime is substantially shorter directly after the NPF event, caused by the efficient decrease in particle number due to self-coagulation. Larger particles show only little changes, as can be seen from an almost un-influenced size distribution for particles larger than 50 nm (Supplementary Fig. 1a, b).

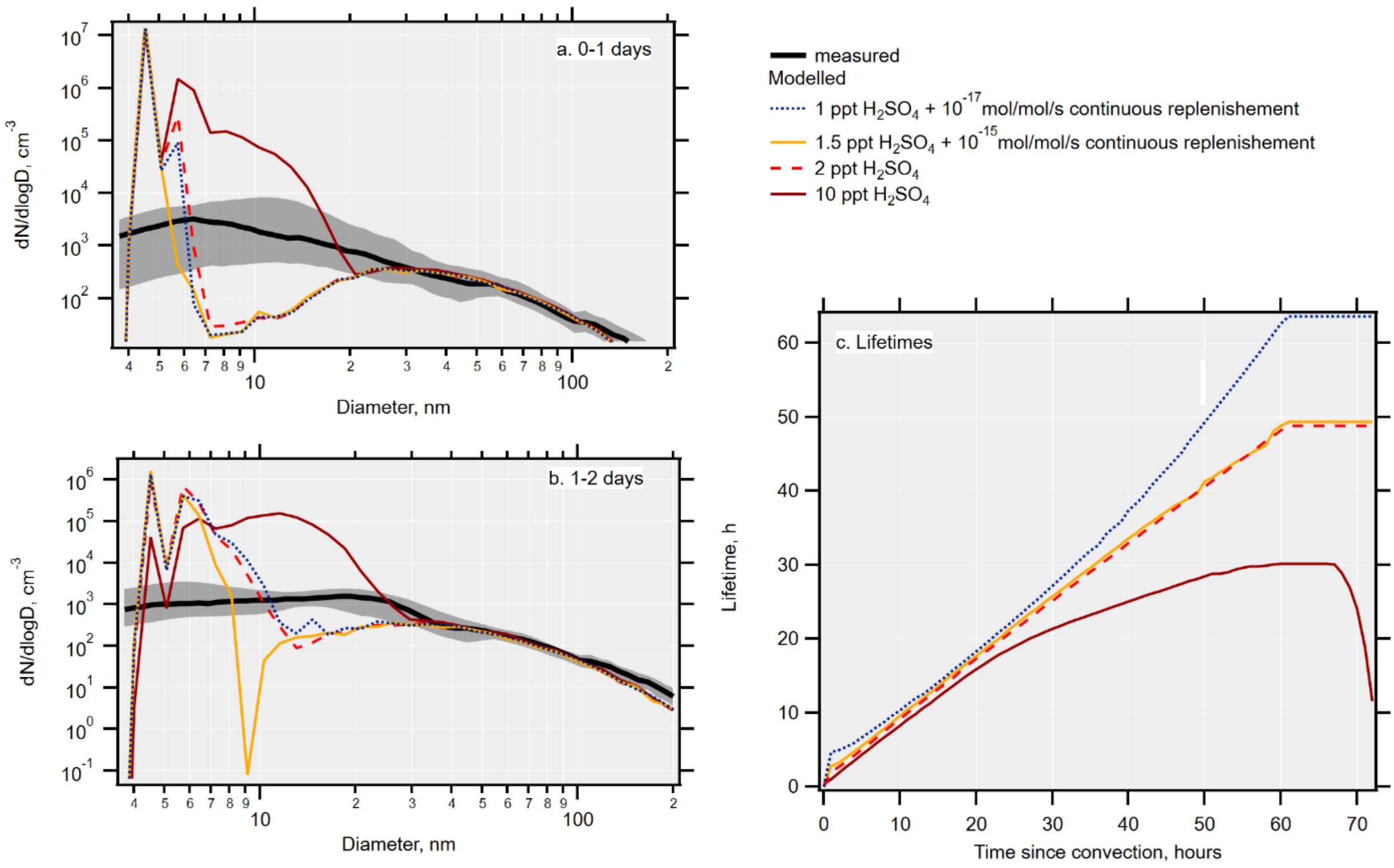

Supplementary Figure 1. Mean modelled size distributions (dN/dlogD) from the GMXe model compared with observed median and interquartile range (IQR) size distributions (black solid line and shading respectively) for (a) 0-1 days, and (b) 1-2 days after convection. (c) Lifetimes of 3-14 nm particles for modelled size distributions determined by the relative change of the burden over the loss rate of those particles**.** Blue dotted lines indicate the model run with 1 ppt of sulphuric acid (H_2SO_4) with a continuous replenishment of 10^{-17} mol/mol/s. Orange solid lines indicate 1.5 ppt H_2SO_4 with a continuous replenishment of 10^{-15} mol/mol/s. Red dashed lines and dark red solid lines indicate 2 and 5 ppy H_2SO_4 with no continuous replenishment.

SD3. Additional Aerosol Properties

Aerosol concentrations observed on ACCLIP in ASM outflow normalized by CO to account for dilution (Supplementary Fig. 2 a, b, SM 2) show the same evolution with time since convection as the total concentrations presented in Fig. 2. Aerosol surface area in the accumulation mode (60-1000 nm) increases with time since convection, as do accumulation mode and total aerosol volume (Supplementary Fig. 2 c, d).

Calculation of optical properties is described in SM section 2. The calculated scattering coefficient increases and the radiative forcing efficiency (RFE) decreases (increases in magnitude) with time since convection (Supplementary Fig. 2 e, f respectively), reflecting the growth of particles formed via NPF in the monsoon outflow.

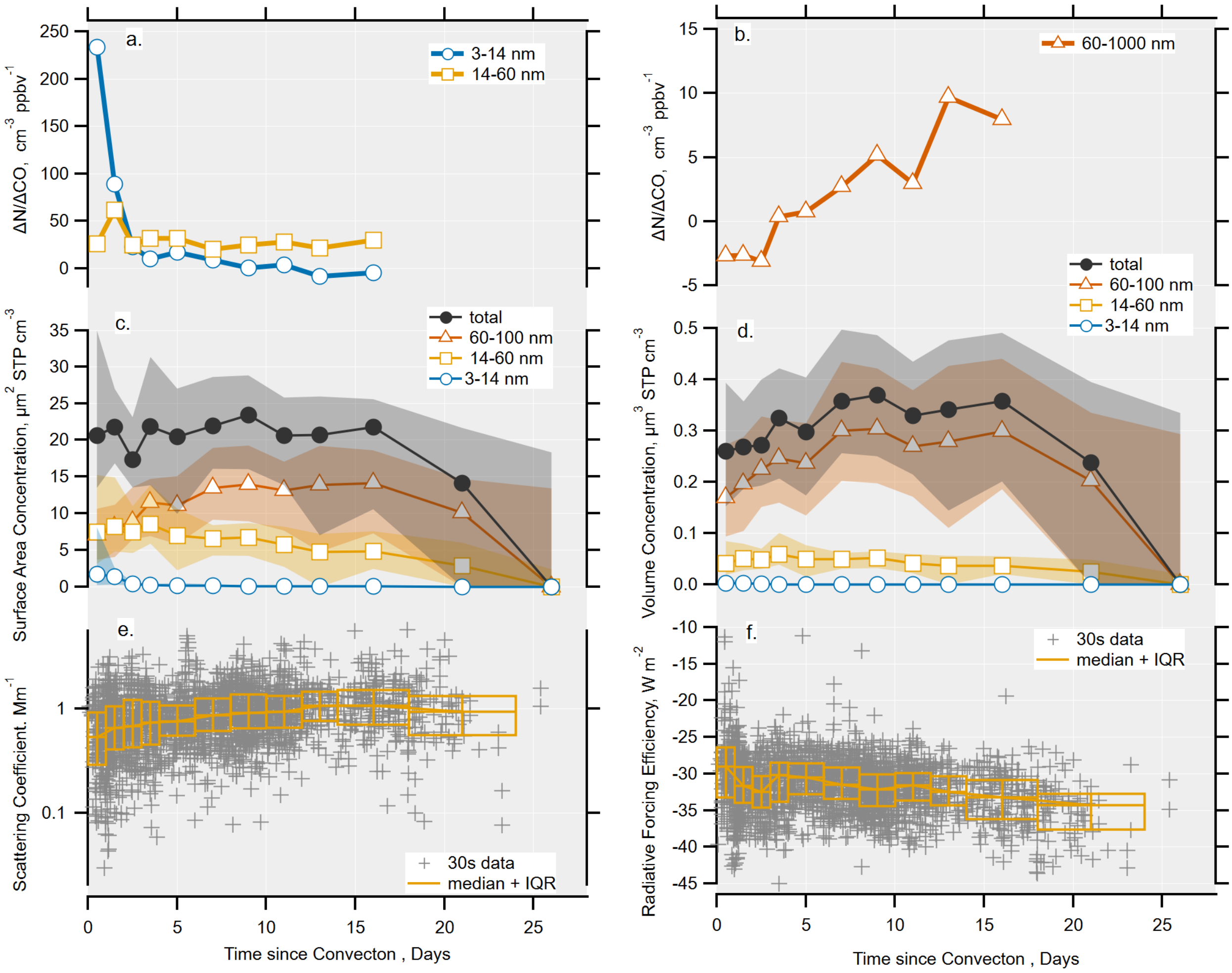

Supplementary Figure 2. Additional Asian Summer Monsoon Chemical and Climate Impact Project (ACCLIP) observed aerosol properties. Normalised excess mixing ratio (ΔN/ΔCO) of number concentrations of particles with diameters (a) 3-14 nm (blue, circles) and 14-60 nm (orange, squares), and (b) 60-1000 nm (vermillion, triangles). The axis for 60-1000 nm diameter particles is on a larger scale to make visible the increase in particle number concentration in this size range. Surface area (c) and volume (d) concentrations observed in Asian Summer Monsoon outflow on ACCLIP, total, and by aerosol mode as a function of time since convection. e) scattering coefficient and (f) radiative forcing efficiency as a function of time since convection, 30s data in grey, median and interquartile ranges in blue.

SD4. Diurnal Cycle

Sampling from the GV aircraft on ACCLIP mainly took place between the hours of 1000 and 1600 solar time (calculated as 8h52m after UTC for the entire campaign) (Supplementary Fig. 3). Within this period, observations with significant NPF (calculated by the relative differencing method detailed in the methods section) peaked at around 1100 UTC in terms of total number of events and fraction of observations, but the fraction of observations was similar within the hours of 1000 and 1700 local solar time (Supplementary Fig. 3). This does not provide strong evidence either for or against a diurnal cycle in NPF events, since observations were mostly made around solar noon.

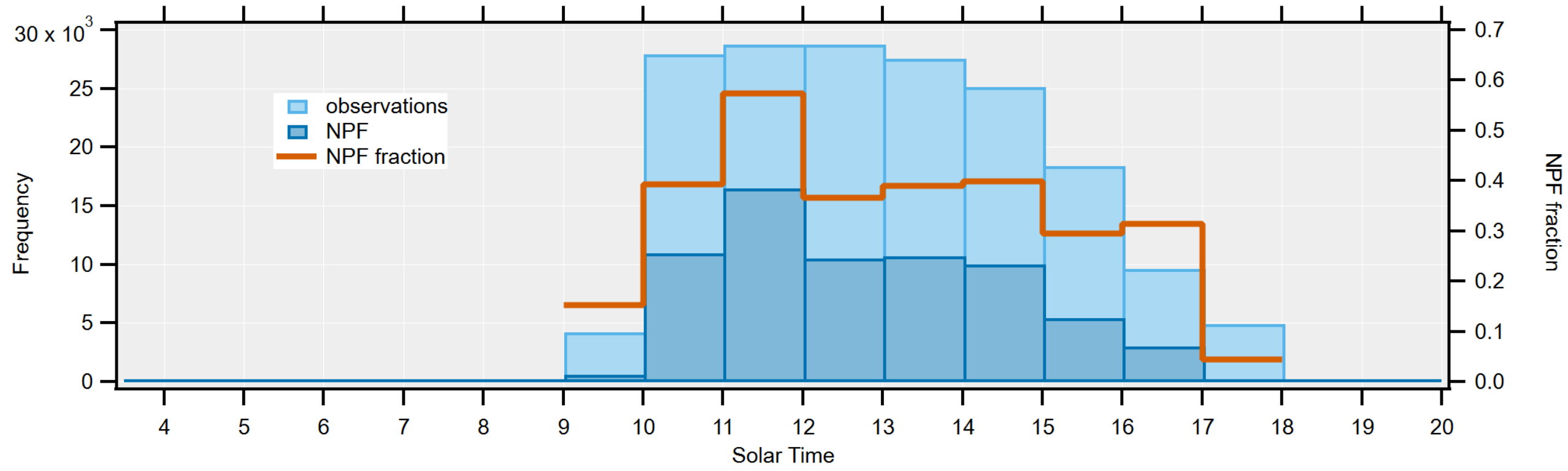

Supplementary Figure 3. Histogram of solar hour of day when Asian Summer Monsoon Chemical and Climate Impact Project (ACCLIP) observations were taken (light blue bars) and when new particle formation (NPF) was indicated (dark blue bars). The fraction of observations in each hour that showed NPF is shown in the vermilion line.

SD5. Fate of ASM Convective Outflow Airmasses

Within 30 days of the observations 15 % of forward trajectories launched from ACCLIP GV flight tracks above 330 K (see Methods) cross the tropopause into the stratosphere (Fig. 10 g). 85 % of the analysed air parcels remain in the troposphere, where they exit the ASM region and subsequently descend over the next 30 days, covering most of the northern hemisphere within 20 days and most of the globe except for southern high latitudes within 30 days (Fig. 10).

A detailed forward trajectory analysis from ACCLIP observations from the WB57 aircraft over 60 days showed 20% of air parcels indicating NPF ascend into the stratosphere[11]. The higher flight altitudes of the WB57 compared to the GV, longer forward trajectories, selection of NPF parcels compared with all flight track point above 300 K, and different vertical resolution in Ueyama et al. [11] likely account for the difference in fraction reaching the stratosphere. The fraction of trajectories entering the stratosphere in both these studies is smaller than that found by Garny and Randel [12] (31-48 %) looking at 60-day trajectories starting at 360K potential temperature.

Similarly to other studies of ASM stratosphere transport pathways[12-17], we observe two primary modes of transport into the stratosphere: 1. Crossing the local tropopause within a few days of observation, likely within the ASM anticyclonic motions, and 2. Crossing the tropopause further north in the region of the subtropical jet (Fig. 10).

Supplementary Methods

SM1. Microphysical aerosol model

Microphysical aerosol model calculations were conducted with an extended box model version of the GMXe aerosol scheme[18]. GMXe is a two-moment modal aerosol scheme, prognostically determining mass and numbers, and diagnosing particle radius given limits for mode radius boundaries and a fixed width of the individual mode distribution. In contrast to the traditional approach of representing the size distribution with four lognormal modes and a relatively wide mode width (as done for global scale simulations with GMXe e.g.[19]), this box model version uses 50 individual lognormal modes with a very narrow width almost representing a highly resolved sectional aerosol scheme. The treated compounds for the presented calculations are H_2SO_4, HNO_3, NH_3 and H_2O and their corresponding ions. The partitioning of those compounds, i.e., the condensation, between the gas and the particulate phase is determined with the thermodynamic equilibrium model ISORROPIA[20]. To account for kinetic limitations, gas phase diffusion onto the existing modes is calculated explicitly and limits the potential phase changes. Nucleation follows a basic H_2SO_4-H_2O system as described by Vehkamäki, et al.[21] and the model takes into account that condensation is energetically preferred to NPF. For coagulational growth, the coagulation kernel of each mode with each other mode is calculated and sequentially applied, such that the newly formed particles can interact with each other, as well as with all particles in all other modes.

The 50 logarithmically spaced size bins between 3 nm and 2µm have mode width sigma set to 1.03 for the smaller classes up to 1.05 for the larger classes. The model is initialised with fixed gas phase mixing ratios of NH_3 (0.1 ppb_v), HNO_3 (0.01 ppb_v) (as constrained by observations and estimates from the STRATOCLIM campaign[22]), and variable H_2SO_4 mixing ratios. A background distribution comparable to the observed particle distribution is initialised allowing for interactions of the freshly nucleated particles with the background aerosol population. Temperature, relative humidity and pressure values are kept constant during the emissions and represent mean conditions for the UTLS as reported for the ACCLIP campaign. A set of scenarios has been simulated, using different initial H_2SO_4 mixing ratios (1, 1.5, 2, 10 ppt_v). The simulations for 2 and 10 ppt_v H_2SO_4 were performed without any further pseudo-emissions, whereas the 1ppt_v and 1.5 ppt_v simulations included constant continuous emissions of NH_3, HNO_3 and H_2SO_4 at rates of 10^{-17} and 10^{-15} mol/mol/s, respectively, leading to subsequent condensational growth.Because of differences between GMXE modelled and observed size distributions (Supplementary Fig. 1), a range of model initial conditions are used to estimate the lifetime of observed nucleation mode aerosol.

SM2. Dilution calculations

We calculated excess mixing ratios for CO and particle number concentrations (N) by subtracting background concentrations similarly to biomass burning studies[23]. We estimated background concentration as the median of observations with longest time since convection that we observed and calculated normalised excess mixing ratio (NEMR) as

$$\frac{\Delta N}{\Delta CO} = \frac{N - N_{background}}{CO - CO_{background}} \qquad \text{(S0)}$$

Assuming that CO remains inert over the time of interest, the NEMR is independent of dilution. Given that CO lifetime is approximately 2 months, we consider the NEMR to account for dilution reasonably well for the first 10 days. Background concentrations are taken as the median values of

number concentrations in each size range and CO for data taken where the mean time since convection is greater than 18 days, which are CO = 82.8 ppbv, $N_{3\text{-}14\,nm}$ = 469.9 cm^{-3}, $N_{14\text{-}60\,nm}$ = 1097.3 cm^{-3}, $N_{60\text{-}1000\,nm}$ = 478.8 cm^{-3}.

SM3. Scattering Calculations

The particle number size distributions were used for calculating light scattering and absorption coefficients and radiative forcing efficiency of aerosols. The chemical composition was first used for estimating volume fractions of the compounds and the effective refractive indices of the aerosols. These were used in Mie modelling to calculate the aerosol optical properties.

Chemical composition was taken from observations of non-refractory aerosol bulk composition (see Methods), and refractory black carbon (rBC) observations from the Single Particle Soot Photometer (SP2, Droplet Measurement Technologies, Longmont, CO[24], see Methods).

SM3.1 Estimation of effective densities and refractive indices

We use bulk chemical composition of concentrations of the inorganic ions ammonium (NH_4^+), sulfate (SO_4^{2-}), nitrate (NO_3^-), organic compounds (Org) and refractory black carbon (rBC), assuming the constituent volume fractions are there throughout the measured particle number size distributions. There are no refractive indices or densities of the above-mentioned inorganic ions as such but there are published values for several compounds formed of them. Therefore, the concentrations of some major compounds were first estimated.

To estimate the concentration of H_2SO_4, $(NH_4)_2SO_4$, HNO_3 and NH_4NO_3 the following process was used. 1) SO_4^{2-} can be either in sulfuric acid SA (H_2SO_4) or ammonium sulphate AS ($(NH_4)_2SO_4$), 2) NO_3^- can be either in nitric acid NA (HNO_3) or ammonium nitrate AN (NH_4NO_3), 3) ammonia (NH_3) reacts first with the stronger acid SA and forms AS and the possibly remaining NH_3 reacts with NA and forms AN. Excess sulphate excSO_4^{2-} is the sulphate that is not as AS[25,26]. Its mass concentration is

$$m(\mathrm{excSO_4^{2-}}) = \mathrm{m}(\mathrm{SO_4^{2-}}) - \mathrm{m}\left(\mathrm{NH_4^+} \cdot \left(\frac{\mathrm{SO_4^{2-}}}{\mathrm{NH_4^+}}\right)_{\mathrm{AS}}\right) = \mathrm{m}(\mathrm{SO_4^{2-}}) - \mathrm{m}(\mathrm{NH_4^+})\frac{96}{2\cdot 18} = \mathrm{m}(\mathrm{SO_4^{2-}}) - \mathrm{m}(\mathrm{NH_4^+})\frac{96}{36} \quad \text{(S1)}$$

where 18 and 96 are the molar masses of ammonium and sulphate, respectively. If m(excSO_4^{2-}) > 0 it is considered to be SA, the mass concentration of which is calculated from

$$m(\mathrm{SA}) = \mathrm{m}(H_2\mathrm{SO_4}) = \mathrm{m}(\mathrm{excSO_4^{2-}})\left(\frac{H_2\mathrm{SO_4}}{\mathrm{SO_4^{2-}}}\right) = \frac{98}{96}m(\mathrm{excSO_4^{2-}}) \quad \text{(S2)}$$

and all ammonium is in AS:

$$m(\mathrm{AS}) = \mathrm{m}\left(\mathrm{NH_4^+} \cdot \left(\frac{(\mathrm{NH_4})_2\mathrm{SO_4}}{\mathrm{NH_4^+}}\right)_{\mathrm{AS}}\right) = \mathrm{m}\left(\mathrm{NH_4^+} \cdot \frac{2\cdot 18+96}{2\cdot 18}\right) = \frac{132}{36}m(\mathrm{NH_4^+}) \quad \text{(S3)}$$

If m(excSO_4^{2-}) < 0, all sulphate is in AS:

$$m(\mathrm{AS}) = \mathrm{m}\left(\mathrm{SO_4^{2-}} \cdot \left(\frac{(\mathrm{NH_4})_2\mathrm{SO_4}}{\mathrm{SO_4^{2-}}}\right)_{\mathrm{AS}}\right) = \frac{132}{96}m(\mathrm{SO_4^{2-}}) \quad \text{(S4)}$$

and there is non-AS ammonium:

$$m(\text{non-AS-NH}_4^+) = \text{m}(\text{NH}_4^+) \text{ - m}((\text{NH}_4)_2\text{SO}_4) \cdot \left(\frac{\text{NH}_4^+}{(\text{NH}_4)_2\text{SO}_4}\right)_{\text{AS}} = \text{m}(\text{NH}_4^+) \text{ - m}(\text{SO}_4^{2-}) \cdot \frac{36}{96} \quad \text{(S5)}$$

If m(non-AS-NH_4^+) > 0 and there is enough NO_3^- all non-AS-NH_4^+ is AN and there is excess NO_3^-:

$$m(\text{excNO}_3^-) = \text{m}(\text{NO}_3^-) \text{ - m}(\text{non-AS-NH}_4^+) \cdot \left(\frac{\text{NO}_3^-}{\text{NH}_4^+}\right)_{\text{AN}} = \text{m}(\text{NO}_3^-) \text{ - m}(\text{non-AS-NH}_4^+) \cdot \frac{62}{18} \quad \text{(S6)}$$

Excess nitrate is considered to be NA:

$$m(\text{NA}) = \text{m}(\text{HNO}_3) = \text{m}(\text{excNO}_3^-) \left(\frac{\text{HNO}_3}{\text{NO}_3^-}\right) = \frac{63}{62} m(\text{excNO}_3^-) \quad \text{(S7)}$$

and AN concentration equals

$$m(\text{NH}_4\text{NO}_3) = \text{m}(\text{non-AS-NH}_4^+) \left(\frac{\text{NH}_4\text{NO}_3}{\text{NH}_4^+}\right)_{\text{AN}} = \text{m}(\text{non-AS-NH}_4^+) \frac{18+62}{18} = \frac{80}{18} m(\text{non-AS-NH}_4^+) \quad \text{(S8)}$$

To estimate the densities and refractive indices, concentrations of AS, SA, AN, and NA were estimated from the AMS data using the above equations (S1) – (S8). Next the volume concentration $v_i = m_i/\rho_i$ of each constituent i was calculated using the estimated mass concentrations m_i and published densities ρ_i (Supplementary Table 1). The volume concentrations were used for calculating the volume fractions $f_i = v_i / \Sigma v_i$ of each constituent. The Mie modelling of the aerosol optical properties was conducted by assuming that the species measured with the AMS were internally mixed and they were externally mixed with the rBC particles. The effective real and imaginary refractive indices of the particles n_{eff} and k_{eff}, respectively, were calculated using the volume mixing rule $n_{eff} = \sum f_i n_i$ and $k_{eff} = \sum f_i k_i$ where f_i is the volume fraction of the species i and n_i and k_i its real and imaginary refractive indices presented in Supplementary Table 1.

SM3.2 Modelling aerosol optical properties

Aerosol optical properties were modelled for the green wavelength λ = 550 nm. Absorption and scattering coefficients were calculated as

$$\sigma_{ap}(\lambda) = \int Q_a(\lambda, D_p, n_{eff}, k_{eff}) \frac{\pi}{4} D_p^2 n(D_p) dD_p \quad \text{(S9)}$$

$$\sigma_{sp}(\lambda) = \int Q_s(\lambda, D_p, n_{eff}, k_{eff}) \frac{\pi}{4} D_p^2 n(D_p) dD_p \quad \text{(S10)}$$

where Q_a and Q_s are the absorption and scattering efficiencies, D_p the particle diameter, n_{eff} and k_{eff} the real and maginary refractive indices, respectively, and $n(D_p)$ the particle number size distribution. Q_a and Q_s were calculated using the N-Mie Fortran code[27]. The single-scattering albedo (SSA, ω_0) was calculated from $\omega_0 = \sigma_{sp} / (\sigma_{sp} + \sigma_{ap})$. The average upscatter fraction β was calculated from the scattering-weighted average asymmetry parameter g[28] according to Moosmüller and Ogren [29]. The larger β is, the more aerosols scatter light to space and cool the atmosphere or heat it less if aerosol so dark that it heats the atmosphere. Radiative forcing efficiencies (RFE = ΔF/τ) at the top of the atmosphere, i.e., aerosol forcing per unit optical depth (τ), were calculated using the formula

$$\frac{\Delta F}{\tau} = -DS_0 T_{at}^2 (1 - A_c) \omega_0 \beta \left\{ (1 - R_s)^2 - \left(\frac{2R_s}{\beta}\right) \left[\left(\frac{1}{\omega_0}\right) - 1 \right] \right\} \quad \text{(S11)}$$

where D is the fractional day length, S_o is the solar constant, T_{at} is the atmospheric transmission, A_c is the fractional cloud amount, and R_s is the surface reflectance[30]. The non-aerosol-related factors were kept constant: $D = 0.5$, $S_o = 1370$ W m^{-2}, $T_{at} = 0.76$, $A_c = 0.6$, and $R_s = 0.15$ [31].

SM4. Topical Upper Troposphere Observations

In-situ observations from the Atmospheric Tomography Mission (ATom)[32-34] are used to compare bulk aerosol composition in ASM outflow to remote convective outflow (Fig. 7, Methods). The portion of the ATom flight tracks used are shown in Supplementary Fig. 4. ATom global positioning and meteorological data were measured by the meteorological measurement system[35-37].

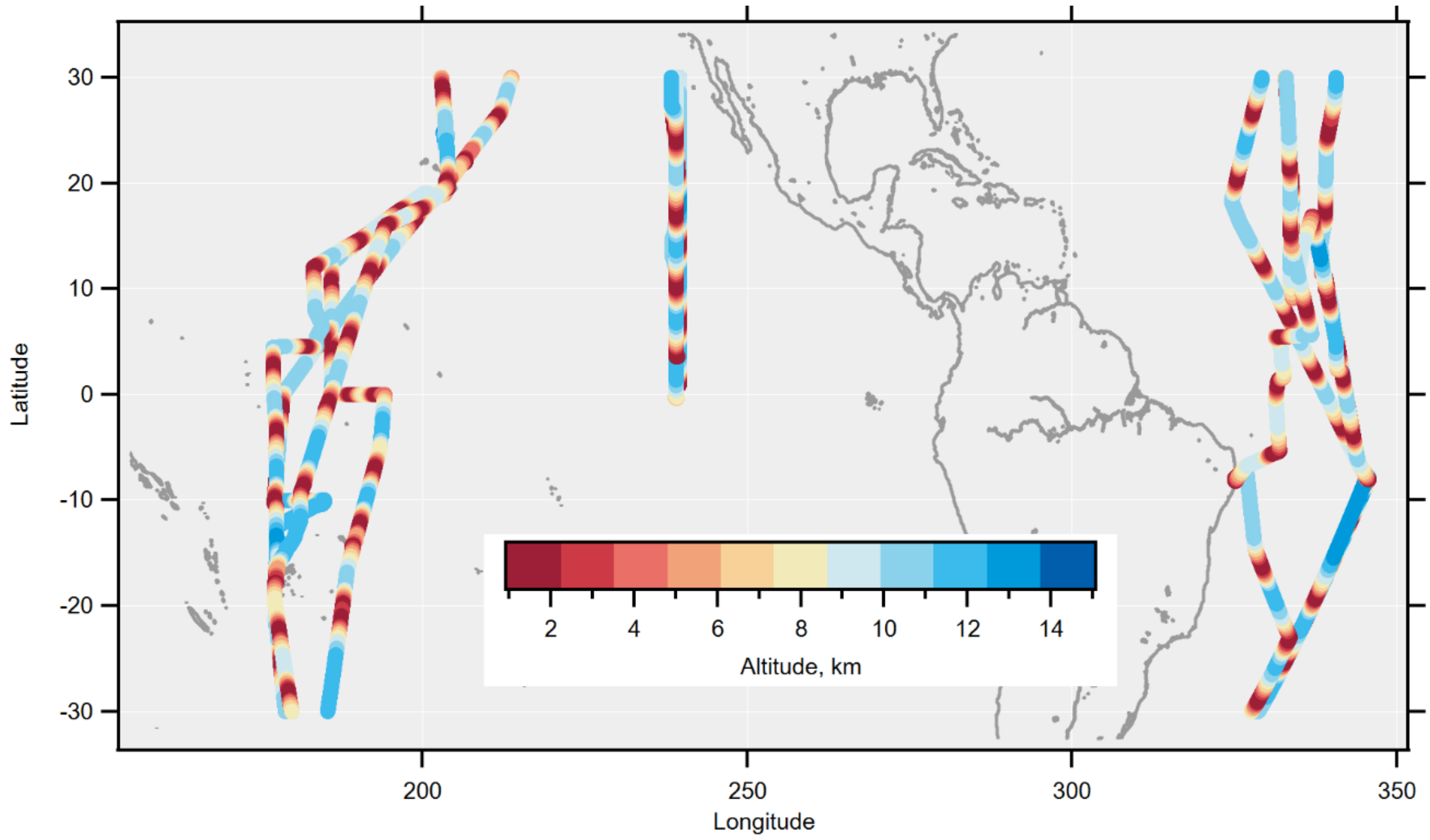

Supplementary Figure 4. Flight tracks from the Atmospheric Tomography Mission between 30°N and 30°S made between 2016 and 2018. Colours show flight altitude. The world map was made with *Natural Earth:* http://www.naturalearthdata.com.

SM5. NMASS modifications

Since the GV aircraft reaches higher altitudes than the DC-8 did during ATom, the internal pressure of the NMASS was changed from 120 hPa and 100 hPa, to ensure it was always below ambient pressure. To keep constant flow rates over this larger ambient pressure range, a system of 3 critical orifices was used to instead of the previous system with only 2 critical orifices. Software was set to automatically change between orifices at 440 hPa and 190 hPa during ascent, and 200 hPa and 450 hPa during descent (the hysteresis preventing oscillating between orifices due to small pressure changes during flight), keeping the total volumetric flow through each NMASS channel between 4.4 and 4.8 cm^3 s^{-1} over the aircraft pressure range 120-880 hPa. Cut-off diameters (particle mobility diameters at which the CPC counts 50% of the particles) for the 5 NMASS CPCs were set to 6.0, 8.3, 14.5, 29.8 and 50.0 nm using calibration techniques described in Williamson, et al.[38]. NMASS counting efficiencies decrease above 200 nm due to impaction in the critical orifice, reaching 50 % detection efficiency at 643 nm (Supplementary Fig. 5).

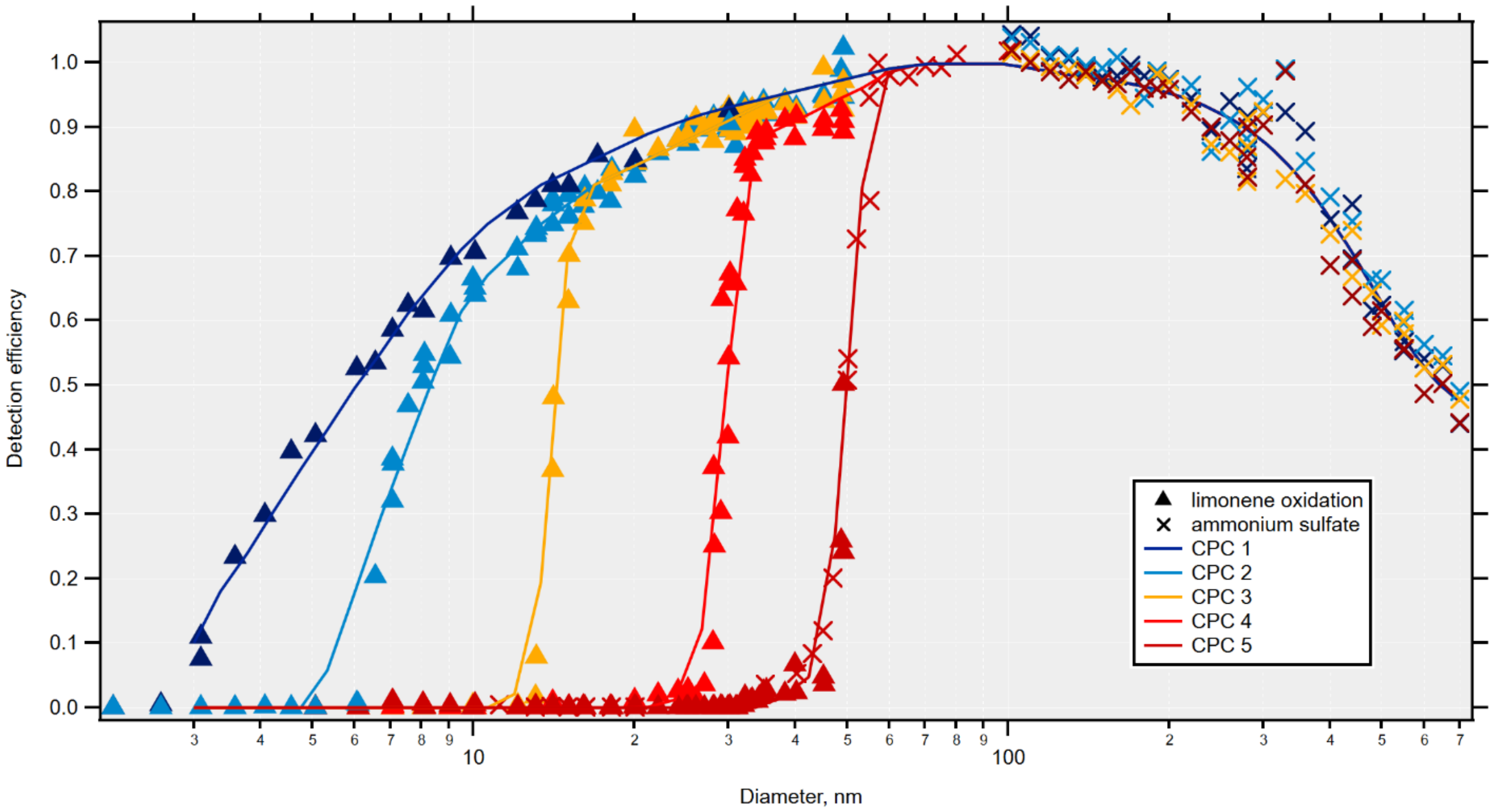

Supplementary Figure 5. NMASS counting efficiency for each of the 5 CPCs calibrated between 2 and 80 nm with limonene oxidation nucleated particles (triangles) and ammonium sulphate nebulizer generated particles (crosses). Solid lines show the inversion kernel calculated from fits to these calibration points. Calibrations above 60 nm were made without CPC 4, which was not functional at the time. Above 60 nm the fit is made for data from CPCs 1, 2, 3 and 5 together, and applied to all 5 channels.

Supplementary Tables

Supplementary Table 1. Densities and real and imaginary refractive indices (n and k, respectively) of aerosol species used in the Mie modelling**.**

Species	density, g cm^{-3}	Reference	n	Reference	k	Reference
SA: H_2SO_4	1.4	[107]	1.426	[39]	0	
AS: $(NH_4)_2SO_4$	1.76	[108]	1.521	[39]	0	
NA: HNO_3	1.4	[107]	1.43	[40]	0	
AN: NH_4NO_3	1.725	[108]	1.554	[41]	0	
Organic Carbon	1.5	[109]	1.55	[42]	-0.005	[43]
rBC	1.8	[109]	1.85	[44]	-0.71	[44]

Supplementary References

1 Bergman, J. W., Fierli, F., Jensen, E. J., Honomichl, S. & Pan, L. L. Boundary layer sources for the Asian anticyclone: Regional contributions to a vertical conduit. *Journal of Geophysical Research: Atmospheres* **118**, 2560-2575, doi:https://doi.org/10.1002/jgrd.50142 (2013).

2 Clemens, J. *et al.* A multi-scenario Lagrangian trajectory analysis to identify source regions of the Asian tropopause aerosol layer on the Indian subcontinent in August 2016. *Atmos. Chem. Phys.* **24**, 763-787, doi:10.5194/acp-24-763-2024 (2024).

3 Vogel, B., Günther, G., Müller, R., Grooß, J. U. & Riese, M. Impact of different Asian source regions on the composition of the Asian monsoon anticyclone and of the extratropical lowermost stratosphere. *Atmos. Chem. Phys.* **15**, 13699-13716, doi:10.5194/acp-15-13699-2015 (2015).

4 Bucci, S. *et al.* Deep-convective influence on the upper troposphere–lower stratosphere composition in the Asian monsoon anticyclone region: 2017 StratoClim campaign results. *Atmos. Chem. Phys.* **20**, 12193-12210, doi:10.5194/acp-20-12193-2020 (2020).

5 Oh, H., Ha, K.-J. & Jeong, J.-Y. Identifying Dynamic and Thermodynamic Contributions to the Record-Breaking 2022 Summer Extreme Rainfall Events in Korea. *Asia-Pacific Journal of Atmospheric Sciences* **60**, 387-399, doi:10.1007/s13143-023-00334-4 (2024).

6 Pan, L. L. *et al.* The Asian Summer Monsoon Chemical and Climate Impact Project (ACCLIP): An Overview. *Journal of Geophysical Research: Atmospheres* **130**, e2025JD044417, doi:https://doi.org/10.1029/2025JD044417 (2025).

7 Pan, L. L. *et al.* East Asian summer monsoon delivers large abundances of very short-lived organic chlorine substances to the lower stratosphere. *Proceedings of the National Academy of Sciences* **121**, e2318716121, doi:doi:10.1073/pnas.2318716121 (2024).

8 Zhang, P., Wang, B., Wu, Z., Jin, R. & Cao, C. Intensified gradient La Niña and extra-tropical thermal patterns drive the 2022 East and South Asian "Seesaw" extremes. *npj Climate and Atmospheric Science* **7**, 47, doi:10.1038/s41612-024-00597-2 (2024).

9 Smith, W. P. *et al.* Transport by Asian Summer Monsoon Convection to the Upper Troposphere and Lower Stratosphere During ACCLIP (2022). *Journal of Geophysical Research: Atmospheres* **130**, e2024JD042732, doi:https://doi.org/10.1029/2024JD042732 (2025).

10 Eppers, O. *et al.* Regional contributions to the chemical composition of the Asian tropopause aerosol layer (ATAL). doi:10.22541/essoar.175130383.35260852/v1 (2025).

11 Ueyama, R. *et al.* On the Fate of Aerosols Produced by New Particle Formation in the Upper Troposphere and Lower Stratosphere. *Geophysical Research Letters* **52**, e2025GL116860, doi:https://doi.org/10.1029/2025GL116860 (2025).

12 Garny, H. & Randel, W. J. Transport pathways from the Asian monsoon anticyclone to the stratosphere. *Atmos. Chem. Phys.* **16**, 2703-2718, doi:10.5194/acp-16-2703-2016 (2016).

13 Honomichl, S. B. & Pan, L. L. Transport From the Asian Summer Monsoon Anticyclone Over the Western Pacific. *Journal of Geophysical Research: Atmospheres* **125**, e2019JD032094, doi:https://doi.org/10.1029/2019JD032094 (2020).

14 Jesswein, M. *et al.* Tracing elevated abundance of CH2Cl2 in the subarctic upper troposphere to the Asian Summer Monsoon. *EGUsphere* **2025**, 1-31, doi:10.5194/egusphere-2024-3946 (2025).

15 Pan, L. L. *et al.* Transport of chemical tracers from the boundary layer to stratosphere associated with the dynamics of the Asian summer monsoon. *Journal of Geophysical Research: Atmospheres* **121**, 14,159-114,174, doi:https://doi.org/10.1002/2016JD025616 (2016).

16 Ploeger, F., Konopka, P., Walker, K. & Riese, M. Quantifying pollution transport from the Asian monsoon anticyclone into the lower stratosphere. *Atmos. Chem. Phys.* **17**, 7055-7066, doi:10.5194/acp-17-7055-2017 (2017).

17 Vogel, B. *et al.* Lagrangian simulations of the transport of young air masses to the top of the Asian monsoon anticyclone and into the tropical pipe. *Atmos. Chem. Phys.* **19**, 6007-6034, doi:10.5194/acp-19-6007-2019 (2019).

18 Pringle, K. J. *et al.* Description and evaluation of GMXe: a new aerosol submodel for global simulations (v1). *Geosci. Model Dev.* **3**, 391-412, doi:10.5194/gmd-3-391-2010 (2010).

19 Tost, H. Chemistry–climate interactions of aerosol nitrate from lightning. *Atmos. Chem. Phys.* **17**, 1125-1142, doi:10.5194/acp-17-1125-2017 (2017).

20 Fountoukis, C. & Nenes, A. ISORROPIA II: a computationally efficient thermodynamic equilibrium model for K+–Ca2+–Mg2+–NH4+–Na+–SO42−–NO3−–Cl−–H2O aerosols. *Atmos. Chem. Phys.* **7**, 4639-4659, doi:10.5194/acp-7-4639-2007 (2007).

21 Vehkamäki, H. *et al.* An improved parameterization for sulfuric acid–water nucleation rates for tropospheric and stratospheric conditions. *Journal of Geophysical Research: Atmospheres* **107**, AAC 3-1-AAC 3-10, doi:https://doi.org/10.1029/2002JD002184 (2002).

22 Höpfner, M. *et al.* Ammonium nitrate particles formed in upper troposphere from ground ammonia sources during Asian monsoons. *Nature Geoscience* **12**, 608-612, doi:10.1038/s41561-019-0385-8 (2019).

23 Akagi, S. K. *et al.* Emission factors for open and domestic biomass burning for use in atmospheric models. *Atmos. Chem. Phys.* **11**, 4039-4072, doi:10.5194/acp-11-4039-2011 (2011).

24 Berberich, J. *et al.* Black Carbon Reflects Extremely Efficient Aerosol Wet Removal in Monsoonal Convective Transport. *Journal of Geophysical Research: Atmospheres* **130**, e2024JD042692, doi:https://doi.org/10.1029/2024JD042692 (2025).

25 Zhuang, H., Chan, C. K., Fang, M. & Wexler, A. S. Formation of nitrate and non-sea-salt sulfate on coarse particles. *Atmospheric Environment* **33**, 4223-4233, doi:https://doi.org/10.1016/S1352-2310(99)00186-7 (1999).

26 Virkkula, A. *et al.* Chemical size distributions of boundary layer aerosol over the Atlantic Ocean and at an Antarctic site. *Journal of Geophysical Research: Atmospheres* **111**, doi:https://doi.org/10.1029/2004JD004958 (2006).

27 Voshchinnikov, N. V. & Mathis, J. S. Calculating cross sections of composite interstellar grains. *Astrophys J* **526**, 257-264, doi:Doi 10.1086/307997 (1999).

28 Andrews, E. *et al.* Comparison of methods for deriving aerosol asymmetry parameter. *Journal of Geophysical Research: Atmospheres* **111**, doi:https://doi.org/10.1029/2004JD005734 (2006).

29 Moosmüller, H. & Ogren, J. A. Parameterization of the Aerosol Upscatter Fraction as Function of the Backscatter Fraction and Their Relationships to the Asymmetry Parameter for Radiative Transfer Calculations. *Atmosphere* **8**, 133 (2017).

30 Sheridan, P. J. & Ogren, J. A. Observations of the vertical and regional variability of aerosol optical properties over central and eastern North America. *Journal of Geophysical Research: Atmospheres* **104**, 16793-16805, doi:https://doi.org/10.1029/1999JD900241 (1999).

31 Haywood, J. M. & Shine, K. P. The effect of anthropogenic sulfate and soot aerosol on the clear sky planetary radiation budget. *Geophysical Research Letters* **22**, 603-606, doi:https://doi.org/10.1029/95GL00075 (1995).

32 Kupc, A. *et al.* The potential role of organics in new particle formation and initial growth in the remote tropical upper troposphere. *Atmos. Chem. Phys.* **20**, 15037-15060, doi:10.5194/acp-20-15037-2020 (2020).

33 Williamson, C. J. *et al.* A large source of cloud condensation nuclei from new particle formation in the tropics. *Nature* **574**, 399-403, doi:10.1038/s41586-019-1638-9 (2019).

34 Thompson, C. R. *et al.* The NASA Atmospheric Tomography (ATom) Mission: Imaging the Chemistry of the Global Atmosphere. *Bulletin of the American Meteorological Society* **103**, E761-E790, doi:10.1175/bams-d-20-0315.1 (2022).

35 Scott, S. G., Bui, T. P., Chan, K. R. & Bowen, S. W. The Meteorological Measurement System on the NASA ER-2 Aircraft. *Journal of Atmospheric and Oceanic Technology* **7**, 525-540, doi:https://doi.org/10.1175/1520-0426(1990)007<0525:TMMSOT>2.0.CO;2 (1990).

36 Gaines, S. E., Bowen, S. W., Hipskind, R. S., Bui, T. P. & Chan, K. R. Comparisons of the NASA ER-2 Meteorological Measurement System with Radar Tracking and Radiosonde Data. *Journal of Atmospheric and Oceanic Technology* **9**, 210-225, doi:https://doi.org/10.1175/1520-0426(1992)009<0210:COTNEM>2.0.CO;2 (1992).

37 Chan, K. R., Scott, S. G., Bui, T. P., Bowen, S. W. & Day, J. Temperature and horizontal wind measurements on the ER-2 aircraft during the 1987 Airborne Antarctic Ozone Experiment. *Journal of Geophysical Research: Atmospheres* **94**, 11573-11587, doi:https://doi.org/10.1029/JD094iD09p11573 (1989).

38 Williamson, C. *et al.* Fast time response measurements of particle size distributions in the 3–60 nm size range with the nucleation mode aerosol size spectrometer. *Atmos. Meas. Tech.* **11**, 3491-3509, doi:10.5194/amt-11-3491-2018 (2018).

39 Kim, S. *et al.* PubChem Substance and Compound databases. *Nucleic Acids Res* **44**, D1202-1213, doi:10.1093/nar/gkv951 (2016).

40 Luo, B., Krieger, U. K. & Peter, T. Densities and refractive indices of H2SO4/HNO3/H2O solutions to stratospheric temperatures. *Geophysical Research Letters* **23**, 3707-3710, doi:https://doi.org/10.1029/96GL03581 (1996).

41 Tang, I. N. Chemical and size effects of hygroscopic aerosols on light scattering coefficients. *Journal of Geophysical Research: Atmospheres* **101**, 19245-19250, doi:https://doi.org/10.1029/96JD03003 (1996).

42 Hand, J. L. & and Kreidenweis, S. M. A New Method for Retrieving Particle Refractive Index and Effective Density from Aerosol Size Distribution Data. *Aerosol Science and Technology* **36**, 1012-1026, doi:10.1080/02786820290092276 (2002).

43 Shamjad, P. M., Satish, R. V., Thamban, N. M., Rastogi, N. & Tripathi, S. N. Absorbing Refractive Index and Direct Radiative Forcing of Atmospheric Brown Carbon over Gangetic Plain. *ACS Earth and Space Chemistry* **2**, 31-37, doi:10.1021/acsearthspacechem.7b00074 (2018).

44 Lack, D. A. & Cappa, C. D. Impact of brown and clear carbon on light absorption enhancement, single scatter albedo and absorption wavelength dependence of black carbon. *Atmos. Chem. Phys.* **10**, 4207-4220, doi:10.5194/acp-10-4207-2010 (2010).
