# Supplementary material for: Upper troposphere aerosol formation enhanced by monsoon lofted pollution
Source: Nat Commun. 2026 Aug 12;17:9672. doi: 10.1038/s41467-026-75073-x (PMC13558689; doi:10.1038/s41467-026-75073-x)
Supplement: Supplementary file 2 [file 41467_2026_75073_MOESM2_ESM.pdf]

nature portfolio

Peer Review File

Upper Troposphere Aerosol Formation Enhanced by Monsoon Lofted Pollution

Corresponding Author: Dr Christina Williamson

This file contains all reviewer reports in order by version, followed by all author rebuttals in order by version.

Attachments originally included by the reviewers as part of their assessment can be found at the end of this file.

Version 0:

Reviewer comments:

Reviewer #1

(Remarks to the Author)

Reviewer #2

(Remarks to the Author)
This paper investigates how lofted pollution influence the new particle formation (NPF) and subsequent growth in the upper troposphere and lower stratosphere. The authors examined the evolution of aerosol microphysics following the convection and found that pollution lofted by the Asian Summer Monsoon (ASM) enhances NPF in convective outflow. The results suggest that newly formed particles can grow to sizes sufficient to increase the scattering of solar radiation and to serve as cloud condensation nuclei. This is a very comprehensive study that integrates recent airborne observations, trajectory analysis, satellite observations, and model simulations of aerosol microphysics. The results advance our understanding of NPF in convective outflows and its climatic impact. Overall, the manuscript is well written. I recommend publication after the authors address the following comments.

Major comments:

The authors evaluate the impact of lofted pollution by comparing particle concentrations in ASM outflow to those in remote tropical outflow. However, these outflows differ in several aspects beyond pollutant loadings, and such difference could also influence NPF. For example, the mixing ratio of OH, an oxidant for H2SO4 production, can vary between the tropics and midlatitudes. Stratospheric air intrusion is more frequent in the midlatitudes. The mixing of descending stratospheric air with outflow could elevate OH mixing ratio, accelerate H2SO4 production, and promote NPF. Anticyclones formed by the Asian summer monsoon can drive sudden increases in deep stratospheric intrusion (e.g., Liu et al., 2024), and new particles have been observed in mixtures of cloud outflow and descending stratospheric air over midlatitude oceans (Zhang et al., 2024). Temperature differences between tropical and midlatitude outflows may also influence nucleation and growth. If feasible, comparing the aerosol concentrations in ASM outflows to those in remote midlatitude outflows may better isolate the effects of lofted pollution.

References:
Liu, X., Fu, Y., Zhang, L., Li, H., Burr, G. S., Bi, Y., and Zhao, G.: Deep stratospheric intrusion events in China revealed on the ground by cosmogenic 10Be/7Be, Communications Earth & Environment, 5, 553, 10.1038/s43247-024-01727-7, 2024.

Zhang, J., Gong, X., Crosbie, E., Diskin, G., Froyd, K., Hall, S., Kupc, A., Moore, R., Peischl, J., Rollins, A., Schwarz, J., Shook, M., Thompson, C., Ullmann, K., Williamson, C., Wisthaler, A., Xu, L., Ziemba, L., Brock, C. A., and Wang, J.: Stratospheric air intrusions promote global-scale new particle formation, Science, 385, 210-216, doi:10.1126/science.adn2961, 2024.

One major conclusion of the paper is that newly formed particles grow and increase the concentration of accumulation mode particles. One key piece of evidence is that accumulation mode concentration increases with the time since convection.

However, because background air typically contains substantially more accumulation-mode particles than fresh convective outflow, this trend could largely reflect entrainment of background air rather than in-situ growth. Furthermore, if growth of the new particles contributes substantially to the accumulation mode concentration, one would expect higher accumulation mode concentrations in outflows with CO > 150 ppbv than in those with CO < 150 ppbv. However, Fig. 3c appears to show the opposite.

Minor comments:

Figure 2 shows that the NPF fraction within two days of convection is ~80%, substantially higher than those after two days (e.g., mostly 20-30%). What proportion of all measurements fall within the 0-2 day window? That could provide insights into the relative importance of NPF initiated in convective outflow versus NPF occurring >2 days later, potentially via different mechanisms. For example, if 20% of samples are within 0–2 days and 80% are >2 days, for a first-order estimate, the contributions to overall NPF incidence would be comparable—0.20×0.80 ≈ 0.16 for outflow-related NPF versus 0.80×0.25 ≈ 0.20 for NPF possibly via different mechanisms.

The authors assess the impact of lofted pollution on particle formation and growth by comparing particle concentrations in ASM outflows to those in remote tropical convective outflows. While a higher particle growth rate in ASM outflows is very plausible, elevated particle concentrations alone do not demonstrate faster growth, especially for the growth to Aitken and accumulation mode size ranges. Increased Aitken and accumulation concentrations could be due to higher initial new particle concentration. Given the higher particle concentration and thus condensation sink, the concentration of condensation species and thus the growth rate are not necessarily higher in lofted pollution with elevated precursor levels.

It appears that Extended Data Fig. 1 is not referenced in the text.

Fig. 3. I would suggest including SO_2 mixing ratios observed in different outflows if possible. It would help illustrate the impact of the key precursor.

Line 119 New particle formation and growth can substantially increase the condensation sink. The condensation sink shown in Fig. 2c is mostly after the NPF, particularly for samples within two days of convection. Therefore, the variation shown in Fig. 2c does not necessarily prove condensation sink (prior to the onset of NPF) is not a controlling factor.

Line 151: redundant words.

Line 53 of Supplementary Information: “SM section 2” instead of “SM section 4”.

Reviewer #3

(Remarks to the Author)
Williamson et al. (2025) present an analysis of particle number concentrations measured during the ACCLIP field campaign. By comparing ACCLIP data with previous campaigns (ATom and StratoClim), the study posits that pollution lofted by the Asian Summer Monsoon (ASM) enhances nucleation in the convective outflow, with particles growing to CCN-relevant sizes within approximately one week.
While the topic is relevant, the manuscript in its current form does not appear to constitute a substantial scientific advance. The enhancement of aerosols and nucleation in the upper troposphere, particularly within the tropics and the ASM, has been extensively documented in prior field campaigns, modeling studies, and laboratory work—many of which are cited by the authors. The potential transport of these pollutant aerosols to lower altitudes and their subsequent contribution to cloud formation is an interesting concept. However, the authors support this claim with speculation rather than quantitative analysis. Consequently, I cannot recommend the manuscript for publication in Nature Communications in its present state.
Line 62: Please define what constitutes a "statistically significant" difference in number concentrations.
Lines 86-89: This analysis is confusing. Please clarify how the conclusion regarding "Time since convection" was reached. Furthermore, what is the major difference between the EASM and SASM that underpins the comparison between ACCLIP and StratoClim? Is it merely that the SASM region is closer to the StratoClim measurement location? Finally, the phrase "this phenomenon" is vague; what is explicitly being suggested for the West African monsoon?
Analysis regarding Fig.3:
The comparison between the tropical UT and the ASM seems problematic. The tropical UT data uses a "before 10-day since convection" criterion, while the timeframe for the ASM data is unclear (3 days?). As demonstrated in Fig. 2, number concentrations are highly sensitive to this timing. Additionally, the dominant aerosol composition in the marine-influenced tropical UT likely differs from that in the pollutant-rich ASM. The observed differences in number concentration could therefore be attributed to various factors, including nucleation pathways, convection strength, and pre-existing particle concentrations and composition.
In the comparison between polluted and less polluted ASM outflow, nucleation mode particle number (N) is higher in more polluted air, yet accumulation mode N is lower near the cloud-top. What is the implication of this inverse relationship?
Lines 116-121: The conclusion that the condensation sink (CS) is not a controlling factor for new particle formation (NPF) requires further elaboration. Figure 2 shows that both nucleation mode particle number (Nd) and CS peak simultaneously within the first day, which seems to suggest a potential relationship rather than its absence.
Given that the cited field campaigns measured aerosol composition and likely precursor gases, it is unclear why the authors did not leverage this data to evaluate specific nucleation pathways (e.g., involving organics or other compounds). Instead, the discussion of mechanisms (Lines 122-134) remains largely speculative. A quantitative assessment of the role of boundary layer pollutants—which is the defining feature of the ASM—is crucial yet missing from this analysis.

The abstract makes several claims regarding the regional climatic and air quality impacts of aerosols in the ASM upper troposphere. These assertions appear speculative, as they are not supported by quantitative analysis or modeling within the manuscript. To strengthen the abstract and ensure it accurately reflects the paper's content, I recommend either removing these speculative impact statements or substantially bolstering them with data-driven evidence in the main text.

Reviewer #4

(Remarks to the Author)
I co-reviewed this manuscript with one of the reviewers who provided the listed reports. This is part of the Nature Communications initiative to facilitate training in peer review and to provide appropriate recognition for Early Career Researchers who co-review manuscripts.

Version 1:

Reviewer comments:

Reviewer #1

(Remarks to the Author)
The authors have made substantial revisions and supplements in response to the initial reviewer comments, including adding targeted model simulations, improving data presentation, and supplementing methodological descriptions, which have significantly enhanced the scientific rigor and persuasiveness of the study. The integration of multi-campaign observations, trajectory modeling, and EMAC model sensitivity simulations provides a comprehensive framework to test the core hypothesis. The findings, namely that ASM-lofted pollutants enhance UT NPF and that the resulting particles grow to climate-relevant sizes, are timely and contribute to filling the gap in understanding anthropogenic impacts on UTLS aerosol processes. However, several key issues related to mechanism clarity, data limitations, and quantitative support for conclusions need to be addressed to strengthen the manuscript's rigor and impact.

1. The study uses remote convection over the tropical Pacific/Atlantic (from the ATom mission) as a "low-pollution control." However, this region fundamentally differs from the ASM in meteorological background and natural source emissions. These factors themselves can affect NPF efficiency, not solely pollution levels. Without controlling for non-pollution variables via statistical methods (e.g., meteorological factor matching), the study cannot definitively attribute differences in particle concentrations to pollution lofting.
2. Observations from ACCLIP (2022), StratoClim (2017), and ATom (2016–2018) span multiple years. Interannual variations in ASM intensity and pollutant emissions may have biased results, with no assessment of temporal variability effects.
3. The study defines NPF based on statistical significance of particle concentrations between 6–8 nm but fails to exclude interference from non-NPF processes (e.g., existing particle fragmentation, aerosol coagulation redistribution). While stratospheric air intrusions (SAI) are not the primary driver, this is only inferred indirectly via CO concentrations. Quantitative correlation analysis between SAI signature factors and NPF events is lacking, leaving residual SAI contributions unaddressed.
4. The EMAC model's simulation results are only compared with observations for small particle sizes (<10 nm), with no validation for climate-relevant critical sizes (accumulation mode >60 nm). Additionally, the model does not separate the contributions of anthropogenic vs. natural pollutants, failing to support the core conclusion that "anthropogenic pollution enhances NPF."
5. The study proposes an NH_3-HNO_3-H_2SO_4 nucleation and growth mechanism but faces two critical inconsistencies: first, the correlation between SO_2 and particle number concentrations is significantly weaker than that with CO, failing to confirm sulfuric acid as a key NPF precursor; second, high-resolution observations of NH_3 in the upper troposphere over the ASM are lacking, with the mechanism relying solely on model inferences rather than direct observational support.
6. The conclusion claims particles influence climate via radiation scattering and cloud property modification, but only provides relative changes in AOD (Fig. 3d) without quantitative radiative forcing calculations. Critical cloud microphysical parameters (e.g., CCN activity, cloud droplet number concentration) are neither observed nor simulated. Climate effects are merely qualitative inferences, lacking quantitative support.
7. The study integrates observational data from ACCLIP (GV aircraft), StratoClim (M-55 aircraft), and ATom (DC-8 aircraft). Although it mentions "the same data processing methods," it does not explain the comparison results of calibration biases between instruments from different platforms. It is recommended to supplement consistency validation of cross-platform instruments to avoid instrument systematic errors affecting regional comparison conclusions.
8. ERICA observations show that accumulation mode particles are rich in ammonium and nitrate, but no comparison is made of particle chemical composition differences between the EASM and SASM regions. Given that the EASM is more polluted, supplementing this difference analysis can further support the "pollution-driven NPF" mechanism and explain NPF efficiency differences between different sub-monsoon systems.
9. The authors have supplemented the temporal evolution of SO_2 in Fig. 2 but have not provided dynamic change data for organic components. As potential precursors for nucleation and growth, the post-convection concentration changes of organic components are crucial for understanding NPF mechanisms. Currently, only the statement that "organic components may contribute" is provided without supporting observational data.
10. The authors have provided data on changes in scattering coefficients and radiative forcing efficiency in the supplementary materials but have not clarified whether these changes reach a "significantly impactful" level for climate. As the reviewer noted in the early version of the paper that shows the radiative-related impacts in Extended Data Fig. 8 are not

significant, yet the authors have not provided quantitative validation (e.g., comparison with contributions from other climate factors) to confirm the actual climatic significance of these impacts.
11. The authors have revised the section title and added CCN-related references but have not elaborated on key information such as the activity of NPF particles as CCN (e.g., critical supersaturation) and their potential impacts on cloud droplet number concentration. Current discussions remain qualitative ("particles can act as CCN") without specific analyses linking to cloud properties.

Reviewer #2

(Remarks to the Author)
I greatly appreciate that the authors have thoroughly addressed my comments. I recommend publication after clarification of their response to my original major comment #1.

Zhang et al. focused on air masses not influenced by pollution or continental emissions to isolate the impact of stratospheric air intrusion (SAI). As a result, the criteria used in Zhang et al. identify "clean" air masses influenced by SAI, but they do not necessarily identify polluted air masses influenced by SAI, which likely have higher CO.

Extended Data Figure 7 shows that, in relatively clean air, the highest N_6nm values were observed in SAI air masses rather than in COF. The results also convincingly show that SAI alone cannot explain the largest particle number concentrations and that lofted pollution is the main driver. That said, the results do not exclude the possibility that mixing between descending stratospheric air and lofted pollution could contribute to high particle concentrations. Similar mixing between stratospheric air and convectively lofted "clean" air was noted in Zhang et al. I recommend that the authors clarify these points in the paper.

The authors have addressed all my other comments thoroughly and satisfactorily.

Reviewer #3

(Remarks to the Author)
My review of the authors' response indicates that while they have addressed several methodological details by clarifying statistical definitions, temporal analysis, and removing problematic comparisons. Regarding the nucleation pathways, the authors employ modeling work to compensate for the lack of observations of key species. However, they have not addressed the fundamental question of scientific novelty and significance. This should be decided by the editor.

Reviewer #4

(Remarks to the Author)
I co-reviewed this manuscript with one of the reviewers who provided the listed reports. This is part of the Nature Communications initiative to facilitate training in peer review and to provide appropriate recognition for Early Career Researchers who co-review manuscripts.

Version 2:

Reviewer comments:

Reviewer #1

(Remarks to the Author)
While the authors have addressed and responded to most of my previous comments and concerns, the proposed nucleation mechanism—NH_3–HNO_3–H_2SO_4 nucleation—is still largely inferred from model simulations, and in my view remains debatable; further observational constraints in future will likely be needed to verify it.
As the authors themselves stated in their reply: "...Organics are also abundant in the observed larger particles in ASM outflow (Extended Data Fig. 8, Extended Data Fig. 5g) and may be involved in nucleation and/or growth, similarly to in remote tropical convection 17...."
Accordingly, it cannot be reasonably concluded that organic nucleation is unnecessary to explain the observed particle composition and number concentrations solely based on modeling results.
In addition, one of my main concerns is that the authors emphasize their study focuses on how anthropogenic pollutants influence NPF events in the upper troposphere and the corresponding climatic effects. I do recognize that they have examined CCN number concentration changes from model simulations. However, the main text of the manuscript has not been updated or revised accordingly; the current version remains largely identical to the original submission, with no apparent improvement in scientific content or presentation. As Reviewer 3 pointed out, the authors have not adequately addressed the fundamental question of scientific novelty and significance.
As mentioned in the authors' response, previous modeling studies have already highlighted the role of monsoon convection in lifting NH_3 into the UTLS. Xenofontos et al. (30) used EMAC simulations over the South Asian monsoon and found that UT NPF rates during convective days were 1–2 orders of magnitude higher than during quiescent days, driven by NH_3 uplift. Xenofontos et al. (31) extended this globally and showed that anthropogenic NH_3 emissions coupled with convective transport enhance UTLS NPF rates.

This study therefore needs to clearly articulate what new insights and findings have been achieved beyond those previous studies.

Open Access This Peer Review File is licensed under a Creative Commons Attribution 4.0 International License, which permits use, sharing, adaptation, distribution and reproduction in any medium or format, as long as you give appropriate credit to the original author(s) and the source, provide a link to the Creative Commons license, and indicate if changes were made.
In cases where reviewers are anonymous, credit should be given to 'Anonymous Referee' and the source.
The images or other third party material in this Peer Review File are included in the article's Creative Commons license, unless indicated otherwise in a credit line to the material. If material is not included in the article's Creative Commons license and your intended use is not permitted by statutory regulation or exceeds the permitted use, you will need to obtain permission directly from the copyright holder.
To view a copy of this license, visit https://creativecommons.org/licenses/by/4.0/

Response to reviewer comments on "Upper Troposphere Aerosol Formation Enhanced by Monsoon Lofted Pollution" by Williamson et al.

We thank the reviewers for their comments, which have prompted us to more deeply investigate the observed differences between tropical and ASM convective NPF, explore the mechanisms behind the ASM NPF and its climatic impacts with modelling, and clarify various aspects of the manuscript. We are happy to resubmit here a manuscript with major revisions that more robustly and comprehensively defends our original conclusions. We include below a point-by-point response to reviewer comments, and submit a manuscript text file with changes tracked.

Reviewer 1:

This study evaluates the impact of pollution on the formation of new particles in upper convection based on the particle size distribution observed in the convective outflow in the "Asian Summer Monsoon Chemistry and Climate Impact Project" (ACCLIP) and "StratoClim for Improving Climate Prediction". The measurement results presented here do reveal some interesting features that may provide new insights of the impact of air pollutants from Asia on the formation of new particles in upper troposphere. While it has been known that NPF can take place at very UTLS, little has been known on the anthropogenic contribution to such events especially over the monsoon region in Asia. If this were demonstrated, it would have significant implications and impact on both atmospheric environment and climate especially over the Asian monsoon region where climate have been intertwined with air pollution, as demonstrated in many studies (but few were referred to in this manuscript). As pointed below, however, I have serious concerns with the major findings of the study which aren't rigorous enough to recommend it for publication in the current form.

Major comments: circumstantial evidence

However, the major findings of the study are (1) the NPF present at UTLS, 2) they are formed from gas pollutants brought up by monsoon circulation, 3) the NPF have significant impact on climate. While all these arguments are sound to some degree, but they are also handwaving with little rigorous analyses and modeling to really prove, especially for 2) and 3). To support 2), CO was used as a tracer to differentiate polluted and cleaner air masses. To substantiate 2), one would need to propose a specific NPF mechanism and apply their observation data to simulate and compare against observations. One cannot readily jump to a conclusion that the NPF was caused by the anthropogenetic precursors over that region. It'd need to demonstrate the pathway of the transport of the gases all the way up to those altitudes where environmental conditions are just adequate to form new particles, or it could NPFs that already exist in the lower atmosphere and brought up to the higher altitudes. Likewise, the manuscript lacks any quantitative analysis of any specific events to support the argument of significant impact of the formation of new particles on climate.

We thank the reviewer for raising this important point. We agree that linking observed upper-tropospheric New Particle Formation (NPF) to anthropogenic precursors and establishing its climate relevance requires rigorous model analysis. The manuscript has been revised to strengthen these arguments while clearly acknowledging the limits of the available data.

To address this, we now include targeted EMAC (ECHAM5/MESSy for Atmospheric Chemistry model) simulations spanning the year 2022, including the ACCLIP campaign period (15th July

until 31st August 2022). Our model simulations incorporate all inorganic NPF parameterizations derived from CERN CLOUD experiments to support the findings of this study. The model setup used in this study is identical to that employed in Xenofontos et al. (2024, 2025) that was extensively evaluated in the region of interest.

To address the reviewer's comments, we analyse three simulations:
(i) a baseline simulation,
(ii) a simulation without NPF, and
(iii) a simulation without anthropogenic NOx, SO_2, and NH_3 emissions globally

These are now described in the Methods (Lines 653-671) and the results are shown in the new Figure 3 and Extended Data Figure 5.

Here we discuss all points raised by the referee:

1) <u>NPF is present in the UT region</u>

The results from the baseline simulation have been compared with the observations of the ACCLIP campaign particle number concentrations PNC (see Extended Data Figure 5). The EMAC and the ACCLIP number concentrations are in good agreement over the full 1–15 km altitude range. We note that the observations show elevated nucleation-and-growth-size particle numbers in the UT during ACCLIP, and that the model reproduces the observed PNC magnitude only when NPF is permitted (Figure 3). This supports NPF operating aloft.

The sensitivity simulations in Figure 3 show how anthropogenic emissions influence NPF within the ASM (Fig. 3):

Removing anthropogenic NO_x, SO_2, and NH_3 leads to a pronounced decrease in both $J_{1.7}$ and particle number concentrations. This confirms that pollution-derived precursors play a major and direct role in sustaining NPF.

Importantly, turning off NPF entirely produces the largest drop in particle numbers, demonstrating that active nucleation is essential for the elevated UT particle abundances observed during ACCLIP, and that anthropogenic vapours act to enhance this nucleation—most strongly in the ASM core and to a lesser, but still significant, extent in the outflow. Thus, anthropogenic emissions significantly enhance NPF in the ASM core, but the effect becomes muted downstream in the outflow region, where dilution and mixing reduce the local contribution of polluted air masses. We have added the following text in Lines 114-117, to explain this result in more detail:

"Observed UT small-particle enhancements are consistent with in-situ NPF, and targeted simulations with the EMAC model predict substantial NPF activity in the upper troposphere over the ASM region (Fig. 3 and Extended Data Fig. 5). Sensitivity simulations further show that anthropogenic emissions strongly enhance NPF in the ASM UT."

(2) <u>Aerosols formed from gas pollutants brought up by monsoon circulation</u>

Figure 3 (panel d) shows that removing anthropogenic NOx+SO_2+NH_3 significantly reduces UT (10–15 km) AOD over the Asian monsoon outflow during ACCLIP. This sensitivity experiment

indicates that the presence of anthropogenic precursors emitted at the surface substantially influences modeled UT aerosol.

We added the following text in lines 228-234:

"ECAM model sensitivity analysis (baseline vs. no anthropogenic NOx+SO_2+NH_3 emissions) shows a systematic UT AOD enhancement over the monsoon outflow (Fig. 3d), from anthropogenic-precursor influence in agreement with Vernier et al.[6]. Over the ACCLIP domain, baseline vs. 'no-anthropogenic' yields +60–100% UT (10–15 km) AOD (Fig. 3d), demonstrating the potential magnitude of UT aerosol enhancement from ASM lofted pollution."

We also note the recent modelling work of Xenofontos et al. (2024, 2025), which demonstrates that deep convection in the Asian monsoon system can efficiently transport NH_3 into the UTLS and enhance NPF by orders of magnitude (Xenofontos et al. 2024, 2025). These studies support the plausibility of the transport–NPF linkage we propose over the Asian monsoon anthropogenic emission hotspots.

We have added the following text (Lines 117-123) in the revised manuscript to explain this in more detail:

"Prior modelling studies highlight the role of monsoon convection in lifting NH_3 into the UTLS. Xenofontos et al[30] used EMAC simulations over the South Asian monsoon to show that days with convection produce UT NPF rates that are one to two orders of magnitude higher than quiescent days, driven by NH_3 uplift. Xenofontos et al[31] extended this globally, finding that anthropogenic NH_3 emissions when combined with convective transport enhance UTLS NPF rates. Together these studies provide mechanistic context for the observation–model findings we present here."

(3) <u>Impact of uplifted pollutants on climate</u>

In figure 3 panel d, the AOD sensitivity to precursor emissions (10–15 km) during the ACCLIP period can be seen: within the dashed ACCLIP box (ASM outflow), values are enhanced by greater than +60%, with local maxima approaching ~100% when anthropogenic emissions are included. Anthropogenic precursors can roughly double UT AOD over the region and period studied — evidence of potential climatic relevance.

We have included this explanation in Lines 228-232 of the revised manuscript:

"ECAM model sensitivity analysis (baseline vs. no anthropogenic NOx+SO_2+NH_3 emissions) shows a systematic UT AOD enhancement over the ASM region (Fig. 3d), from anthropogenic-precursor influence in agreement with Vernier et al.[6]. Over the ACCLIP domain, baseline vs. 'no-anthropogenic' yields +60–100% UT (10–15 km) AOD (Fig. 3d), demonstrating the potential magnitude of UT aerosol enhancement from ASM lofted pollution."

We agree that CO alone cannot definitively identify the source of nucleation precursors, and now present more clearly the relationships of newly formed particles and accumulation mode particles with CO and SO2 in a new Extended Data Figure 6, which is referenced in the main text at lines 126-128, 174-175 and 211-213:

“ACCLIP observations show a strong correlation between observed particle number concentrations and CO mass mixing ratio (Extended Data Fig. 6a).”

“Particle number concentrations observed during ACCLIP are not as highly correlated with SO_2 as they are with CO (Extended Data Fig. 6).”

“The magnitude of the accumulation mode number concentration increases with time since convection (Fig. 4b) and is inversely correlated with CO (Extended Data Fig. 6c)”

I don’t see any convincing evidences to support their argument that NPF (1) affects the characteristics of cumulus clouds, (2) affects heterogeneous chemistry in the troposphere and stratosphere, in any figures, data in their main text nor SI, although they are generally sound in principle. To convince the impact, it would be necessary to conduct a modeling study based on the observations. In addition, regarding to the impact on radiation forcing, the authors also stated that the NPF noticeably enhances the scattering of shortwave radiation. Note that scattering is proportional to the collective sections of all aerosol particles. In general, for such tiny particles especially newly formed ones, their scattering ability is negligible According to the Fig 8 in the extended data, it seems that the impact is not that significant. I also suggest move some of the panels in extended data Figure 8 (like Fig. 8e and f) to the main text.

We agree and have revised speculative statements about impacts on cumulus clouds and heterogeneous chemistry to only discuss the potential impact based on particle size and the literature. Thus, the section now between lines 218 and 232 has been revised to read:

“Forward trajectory modelling (see Methods) shows that within 30 days following observation, 15 % of the airmasses sampled by the GV on ACCLIP above 330 K cross the tropopause into stratosphere either locally within the anticyclone or further north in the region of the subtropical jet, while the majority of analysed air parcels descend through the troposphere and disperse on a hemispheric scale (Extended Data Fig. 11, Supplementary Discussion section 3). Potential impacts of particles that have grown into the accumulation mode include contributing to the ATAL[7,22,24,41] and affecting heterogeneous chemistry[40], scattering radiation to space from the UT (Extended Data Fig. 10, Supplementary Discussion section 4), affect the properties of UT cirrus clouds, either through homogeneous ice nucleation, or heterogenous ice-nucleation of ammonium nitrate aerosol[30,37,38,40,42,43], and affecting the properties of warm and mixed-phase clouds as CCN after descent[16]. ECAM model sensitivity analysis (baseline vs. no anthropogenic NOx+SO_2+NH_3 emissions) shows a systematic UT AOD enhancement over the ASM region (Fig. 3d), from anthropogenic-precursor influence, in agreement with Vernier et al.[6]. Over the ACCLIP domain, baseline vs. ‘no-anthropogenic’ yields +60–100% UT (10–15 km) AOD (Fig. 3d), demonstrating the potential magnitude of UT aerosol enhancement from ASM lofted pollution.”

Other discussion is restricted to results directly supported by the observations and model sensitivity experiments (UT ultrafine particle abundance and AOD response). Assessing cloud microphysics or heterogeneous chemistry effects would require dedicated modelling beyond the scope of this study.

Specific comments:

Line 47: Some explanation needs to be given as to why the number concentration reaches its peak near the potential temperature of 360 K?

This is where convective outflow is at a maximum, as shown directly by CO and supported by trajectory modelling studies. This explanation has been added to the text (line 55-57), along with references to the modelling studies (see fig. 5 of ref 22) and a new panel b for fig 1 showing the CO vertical profile as follows.

"This peak coincides with peak convective outflow demonstrated in a maxima of observed concentrations of CO (Fig. 1b) in agreement with trajectory modelling[22, 27]."

The caption of Fig.1 has been modified to describe the additional CO panel. It now reads (line 64-73):

***"Fig. 1 Vertical profile of observed aerosol concentrations and CO in ASM regions.** Total number concentration of particles with dry diameter above 6 nm (a) and volume mixing ratio of CO (b) as a function of potential temperature. Solid lines show median values of all 1Hz data for ACCLIP (black) and StratoClim (orange) with interquartile range (IQR) shaded or in error bars. All 1Hz data for ACCLIP are shown in light grey circles, and those data points with NPF indicated in dark grey crosses. ACCLIP data are then separated into observed airmasses with 80% or greater association with East Asian Summer Monsoon (EASM) convection (light blue, dashed) or South Asian Summer Monsoon (SASM) convection (dark blue, dotted) with the lines showing the median, and IQR as shaded areas. The approximate altitude corresponding to the observed potential temperatures is shown. Panel a also highlights in red dots and dark red crosses ACCLIP observations identified as influenced by stratospheric air intrusions (SAI, see Methods) and therein those indicating NPF respectively."*

Line 62: As mentioned in the text, the definition of NPF events is a significant increase in the observed number concentration between 6-8 nm. However, figure 2 did not clearly shows the characteristics of this particle size range. Conversely, particles between 3-14 nm exhibit a maximum at the initial moment, which seems misleading.

We had originally included just N3-14 since this is the defined "nucleation mode" used across multiple campaigns for consistency, and it generally shows good a agreement with the number concentration in the lowest diameter size bin (in ACCLIP 6-8 nm) which is used for the identification of NPF events. However, we agree with the reviewer that the concentrations from this size bin are also interesting and therefore include them in addition to the N3-14 in fig 2a. N6-8 follows the same behaviour with time as N3-14, only with an even more steep decrease in the first few days after convection, as expected from panel 2. This serves to highlight how nucleation is occurring mainly in the first 2 days after convection, and then particles are lost from the smallest size bins via growth and coagulation losses. We have modified the caption of Fig.2 to reflect this and to improve clarity. It now reads (lines 82-90) as follows:

***"Fig. 2 Observed aerosol and gas phase properties as a function of time since convection during ACCLIP.** (a) Median number concentration in nucleation mode (3-14 nm, blue circles), Aitken mode (14-60 nm, yellow squares), and accumulation mode (60-1000 nm, red triangles) with IQR (shaded) at standard temperature and pressure (std.) The number concentration in*

the first discrete NMASS channel (as opposed to the modes calculated from the inverted size distribution) is shown in black. (b) Fraction of data points indicating NPF (see methods). (c) Condensation sink (rate at which sulphuric acid condenses onto the existing aerosol population, indicating how much this population supresses nucleation) calculated from the full observed size distribution. Pink dots are the 30s data, red line and boxes show median and IQR, respectively. (d) and (e) Mixing ratio of CO and SO_2 respectively, grey dots are 1Hz data, red line and boxes show median and IQR, respectively."

Line 64: The manuscript mentions that significant NPF events can be observed within 2-3 days since the onset of convection, and not particularly afterwards. The particle characteristic information within this interval (time period) should be supplemented. The current version coordinates show that the characteristics of the 2-3 days among 0-28 days are not obvious.

The discussion of ACCLIP NPF within 2 days since convection refs explicitly to fig 2b, which shows 80% NPF fraction in the 0-1 and 1-2 day bins, and 30% or lower in all the bins for 3 days and after. The 3 days refers to the stratoclim paper as referenced. We have expanded the width of fig 2 to make this easier to see.

Line 71: Figure 2c shows that condensation sinks have valley values 3 days since convection, while maintaining high values within 2 days since convection. Here the study explains in Line 119, "indicating that the condensation sink is not the controlling factor for NPF, and highlighting the importance of the availability of condensable vapours in the first days after convection." It is suggested to supplement the evolution of SO2 and organic components since convection similar to the evolution of particle concentration mentioned in the text. The current result only shows the campaign mean of chemical composition and the mixing ratios with the convective outflow seems insufficient.

We have added the time evolution of SO2 to fig 2, although note that caution must be applied to evaluating SO2 and NPF since much is dependent upon the (unmeasured) OH concentrations, and other species participating in nucleation and growth (see new modelling results discussed earlier). We have revised the text to discuss the time evolution of SO2 and clarify the discussion of condensation sinks in lines 164-167 as follows:

"The ASM outflow condensation sink and SO_2 volume mixing ratio both show an initial maximum immediately after convection, coincident with the highest number concentrations of newly formed particles (Fig. 2). This suggests that newly formed particles themselves limit the rate of new particle formation within this system."

The box model simulations (extended data Fig.4) indicate that a substantial amount of additional condensable material must be added to the new particle size distribution to significantly modify the shape of the size distribution. This also supports the statement, that condensation alone represents only a minor pathway for particle growth if insufficient addition of condensable material by e.g., convective transport is available.

Line 135: Regarding climate related impacts, the manuscript focuses on discussing the evolution of particle number size distribution and number concentration since convection,

without delving into the discussion of CCN and cloud related factors, which requires further elaboration.

We have modified the title of the section on climate relevance to *"Growth to Climate Relevant Sizes"* (line188) to more accurately reflect the new knowledge from this section. We have added references in this section to more accurately demonstrate that, while the impacts of larger particles on radiation, cloud properties and heterogeneous chemistry are known from the literature, it had not previously been demonstrated the newly formed particles in ASM outflow grow to these sizes. By demonstrating the growth, as we do here, we link NPF to climate impacts. We have modified the text in lines 189-192 to more accurately reflect this as follows:

"The particles formed in ASM convective outflow are important for climate because they grow to diameters larger than 60 nm (Fig. 4). At these sizes they are large enough to scatter radiation (Fig. 3g, Extended Data Fig. 10), impact cloud properties[34,39-40,42,45,46], and provide surface area for heterogeneous chemistry[42]."

We have also adjusted the text in lines 218-232 to more clearly differentiate between the new knowledge from our forward trajectory modeling, and prior knowledge about aerosol-cloud and aerosol-radiation interactions. It now reads:

"Forward trajectory modelling (see Methods) shows that within 30 days following observation, 15 % of the airmasses sampled by the GV on ACCLIP above 330 K cross the tropopause into stratosphere either locally within the anticyclone or further north in the region of the subtropical jet, while the majority of analysed air parcels descend through the troposphere and disperse on a hemispheric scale (Extended Data Fig. 11, Supplementary Discussion section 3). Potential impacts of particles that have grown into the accumulation mode include contributing to the ATAL[7,22,24,41] and affecting heterogeneous chemistry[40], scattering radiation to space from the UT (Extended Data Fig. 10, Supplementary Discussion section 4), affect the properties of UT cirrus clouds, either through homogeneous ice nucleation, or heterogenous ice-nucleation of ammonium nitrate aerosol[30,37,38,40,42,43], and affecting the properties of warm and mixed-phase clouds as CCN after descent[16]. ECAM model sensitivity analysis (baseline vs. no anthropogenic NOx+SO_2+NH_3 emissions) shows a systematic UT AOD enhancement over the ASM region (Fig. 3d), from anthropogenic-precursor influence, in agreement with Vernier et al.[6]. Over the ACCLIP domain, baseline vs. 'no-anthropogenic' yields +60–100% UT (10–15 km) AOD (Fig. 3d), demonstrating the potential magnitude of UT aerosol enhancement from ASM lofted pollution."

6 Vernier, J.-P. *et al.* Increase in upper tropospheric and lower stratospheric aerosol levels and its potential connection with Asian pollution. *Journal of Geophysical Research: Atmospheres* **120**, 1608-1619, doi:https://doi.org/10.1002/2014JD022372 (2015).

7 Yu, P. *et al.* Efficient transport of tropospheric aerosol into the stratosphere via the Asian summer monsoon anticyclone. *Proceedings of the National Academy of Sciences* **114**, 6972-6977, doi:doi:10.1073/pnas.1701170114 (2017).

16 Williamson, C. J. *et al.* A large source of cloud condensation nuclei from new particle formation in the tropics. *Nature* **574**, 399-403, doi:10.1038/s41586-019-1638-9 (2019).

22 Ueyama, R. *et al.* On the Fate of Aerosols Produced by New Particle Formation in the Upper Troposphere and Lower Stratosphere. *Geophysical Research Letters* **52**, e2025GL116860, doi:https://doi.org/10.1029/2025GL116860 (2025).

24 Mahnke, C. *et al.* The Asian tropopause aerosol layer within the 2017 monsoon anticyclone: microphysical properties derived from aircraft-borne in situ measurements. *Atmos. Chem. Phys.* **21**, 15259-15282, doi:10.5194/acp-21-15259-2021 (2021).

30 Xenofontos, C. *et al.* The impact of ammonia on particle formation in the Asian Tropopause Aerosol Layer. *NPJ Clim Atmos Sci* **7**, 215, doi:10.1038/s41612-024-00758-3 (2024).

37 Yu, P. *et al.* Abundant Nitrate and Nitric Acid Aerosol in the Upper Troposphere and Lower Stratosphere. *Geophysical Research Letters* **49**, e2022GL100258, doi:https://doi.org/10.1029/2022GL100258 (2022).

38 Wang, M. *et al.* Synergistic HNO3–H2SO4–NH3 upper tropospheric particle formation. *Nature* **605**, 483-489, doi:10.1038/s41586-022-04605-4 (2022).

40 Zhu, Y. *et al.* Evaluating the Importance of Nitrate-Containing Aerosols for the Asian Tropopause Aerosol Layer. *Journal of Geophysical Research: Atmospheres* **129**, e2024JD041283, doi:https://doi.org/10.1029/2024JD041283 (2024).

41 Vernier, J.-P., Thomason, L. W. & Kar, J. CALIPSO detection of an Asian tropopause aerosol layer. *Geophysical Research Letters* **38**, doi:https://doi.org/10.1029/2010GL046614 (2011).

42 Cziczo, D. J. & Abbatt, J. P. D. Ice nucleation in NH4HSO4, NH4NO3, and H2SO4 aqueous particles: Implications for cirrus cloud formation. *Geophysical Research Letters* **28**, 963-966, doi:https://doi.org/10.1029/2000GL012568 (2001).

43 Wagner, R. *et al.* Solid Ammonium Nitrate Aerosols as Efficient Ice Nucleating Particles at Cirrus Temperatures. *Journal of Geophysical Research: Atmospheres* **125**, e2019JD032248, doi:https://doi.org/10.1029/2019JD032248 (2020).

To reflect these changes in the manuscript we have modified the concluding paragraph as follows:

Line 251-253 we replace "Comparison with observatio from remore teopical convection" with "Targeted modeling" as the demonstration of lofted pollution increasing particles in the ASM UT.

Lines 254-256 more explicitly refer to growth as follows:
" Observed growth within the ASM region shos that the newly formed particles grow into the accumulation mode (sizes where they can influence climate) in about a week. "

The statement that only air parcels tracke as directly ascending into the stratosphere has been removed from line 257.

The statement of climate impacts in the final sentence (lines 259-263) had been modified to read:

"Therefore, while previous literature has focused heavily on the ASM as a source of particulate matter maintaining the ATAL, the effects of these particles scattering radiation in the UT, with

their potential toalter the properties of cirrus clouds, and act as CCN lower in the troposphere must also be considered when evaluating the overall climate impacts of the ASM.”

Line 333: Suggestions for the Section of Method, 1) Specific Introduction to the Frequency Distribution of NPF in Observations, 2) adding the description of condensation sink calculation.

We have added a description of the NPF fraction calculation to the description to the calculation of NPF events is given in the methods section as follows at lines 465-467. It reads:

“The fraction of data indicating NPF in a given location or during a given time period is calculated as the number of 1 Hz data points indicating NPF by thus method, divided by the total number of 1 Hz data points in the selected period.”

Full description of condensation sink calculations is now made at line 461-474 to replace the previous reference to the method describe in Williamson et al 2019.

Reviewer #2 (Remarks to the Author):

This paper investigates how lofted pollution influence the new particle formation (NPF) and subsequent growth in the upper troposphere and lower stratosphere. The authors examined the evolution of aerosol microphysics following the convection and found that pollution lofted by the Asian Summer Monsoon (ASM) enhances NPF in convective outflow. The results suggest that newly formed particles can grow to sizes sufficient to increase the scattering of solar radiation and to serve as cloud condensation nuclei. This is a very comprehensive study that integrates recent airborne observations, trajectory analysis, satellite observations, and model simulations of aerosol microphysics. The results advance our understanding of NPF in convective outflows and its climatic impact. Overall, the manuscript is well written. I recommend publication after the authors address the following comments.

Major comments:

The authors evaluate the impact of lofted pollution by comparing particle concentrations in ASM outflow to those in remote tropical outflow. However, these outflows differ in several aspects beyond pollutant loadings, and such difference could also influence NPF. For example, the mixing ratio of OH, an oxidant for H2SO4 production, can vary between the tropics and midlatitudes. Stratospheric air intrusion is more frequent in the midlatitudes. The mixing of descending stratospheric air with outflow could elevate OH mixing ratio, accelerate H2SO4 production, and promote NPF. Anticyclones formed by the Asian summer monsoon can drive sudden increases in deep stratospheric intrusion (e.g., Liu et al., 2024), and new particles have been observed in mixtures of cloud outflow and descending stratospheric air over midlatitude oceans (Zhang et al., 2024). Temperature differences between tropical and midlatitude outflows may also influence nucleation and growth. If feasible, comparing the aerosol concentrations in ASM outflows to those in remote midlatitude outflows may better isolate the effects of lofted pollution.

References:

Liu, X., Fu, Y., Zhang, L., Li, H., Burr, G. S., Bi, Y., and Zhao, G.: Deep stratospheric intrusion events in China revealed on the ground by cosmogenic 10Be/7Be, Communications Earth & Environment, 5, 553, 10.1038/s43247-024-01727-7, 2024.

Zhang, J., Gong, X., Crosbie, E., Diskin, G., Froyd, K., Hall, S., Kupc, A., Moore, R., Peischl, J., Rollins, A., Schwarz, J., Shook, M., Thompson, C., Ullmann, K., Williamson, C., Wisthaler, A., Xu, L., Ziemba, L., Brock, C. A., and Wang, J.: Stratospheric air intrusions promote global-scale new particle formation, Science, 385, 210-216, doi:10.1126/science.adn2961, 2024.

We thank the reviewer for raising the questions of how stratospheric intrusions and temperature might influence the ASM-Tropical UT comparison, as indeed we had neglected to explicitly address these. Using the criteria if H2O, O3 and CO set out in Zhang et al. 2024 we include analysis to show that the majority of NPF, and the NPF resulting in the highest number concentrations in ASM outflow was not influenced by stratospheric intrusions (new ED fig. 7). This supports the major conclusion that it is pollution lofted by the ASM that increases the number concentrations resulting from NPF in ASM outflow above those seen in remote topical convective outflow. We also show (ED fig. 7) that NPF in ASM outflow does not predominantly occur at the lowest observed temperatures, and that the highest resulting number concentration are not observed in the coldest airmasses in ASM outflow. This also supports the conclusion that it is additional and/or different lofted gas phase species available for nucleation and growth in the ASM outflow that increases the particle concentration resulting from NPF and growth above those seen in remote convective outflow. Both of these issues are discussed in the text at lines 128-130 *as follows:*

"Furthermore, the largest particle number concentrations resulting from NPF are not associated with stratospheric air intrusions, nor the coldest temperatures in ASM outflow (Fig. 1, Extended Data Figure 7), highlighting the importance of lofted condensable vapours."

We also indicate in the revised Fig. 1 and the new ED Fig 6b airmasses influenced by SAI and show that these do not contain the enhanced particle number concentrations. We describe the identification of SAI in the Methods lines 646-657.

"The potential role of stratospheric air intrusions (SAI) to increase NPF is explored following Zhang et al[90], which defines SAI as low water vapour (10-400 ppm), low CO (< 100 ppb) and high ozone (70-100 ppb), and convective outflow (COF) as high water vapour (300-4000 ppm) and low ozone (30-70 ppb), or high CO (> 100 ppb). Without accounting for CO, ACCLIP UT shows NPF within both SAI and COF zones as defined by Zhang et al[90], as well as a lot of NPF observed these zones (Extended Data Fig. 7). However, the majority of the observed NPF, and especially that associated with the highest number concentrations, is predominantly seen in high CO airmasses (Extended Data Fig. 7). The high CO indicates COF. Therefore, we conclude that the higher number concentrations resulting from NPF observed in ACCLIP UT cannot be explained by more SAI influence. Temperature profiles of the ACCLIP UT observations (Extended Data Fig. 7a) show that NPF, and especially that resulting in the highest number concentrations, is not occurring at the lowest temperatures."

90 Zhang, J., Gong, X., Crosbie, E., Diskin, G., Froyd, K., Hall, S., Kupc, A., Moore, R., Peischl, J., Rollins, A., Schwarz, J., Shook, M., Thompson, C., Ullmann, K., Williamson, C., Wisthaler, A., Xu, L., Ziemba, L., Brock, C. A., and Wang, J.: Stratospheric air intrusions promote global-scale new particle formation, Science, 385, 210-216, doi:10.1126/science.adn2961, 2024

We are grateful for this comment, and those from the other reviewers pointing out a number of difficulties in interpreting the comparison of tropical UT and ASM outflow. With the addition of targeted modelling of the NPF mechanisms and influence of pollution, this comparison no longer added new conclusions, and so we have removed the original Figure 3 and comparisons with topical UT (except for the bulk chemical composition).

For interest, I include a plot here comparing tropical UT and ASM outflow with respect to SAI and temperature.

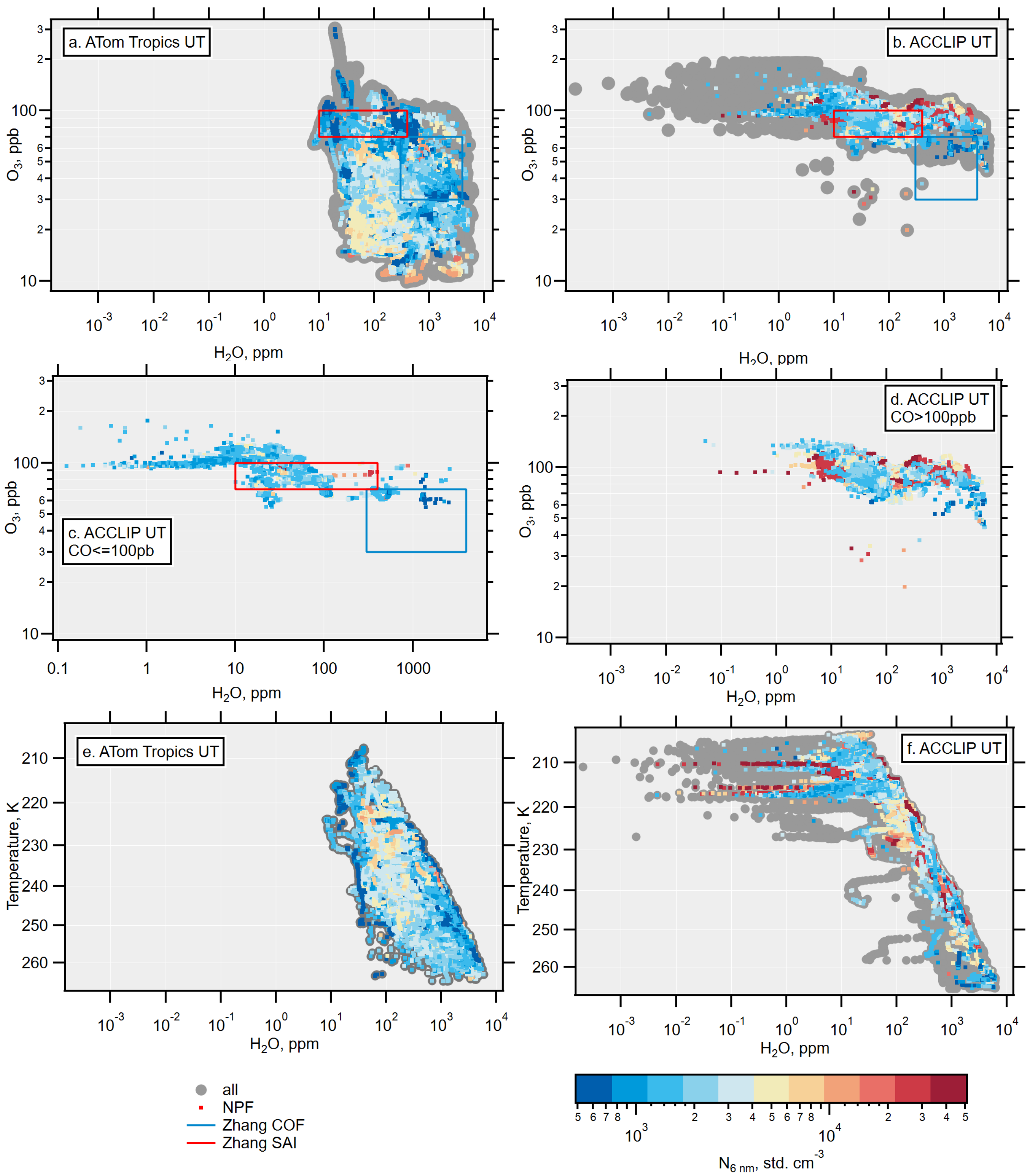

Response to Reviewers Figure 1. Examining the influence of stratospheric air intrusions (SAI) and temperature on NPF in ASM and remote tropical convective outflow. 1 Hz data indicating NPF (coloured dots, with colours indicating the number concentration of particles greater than 6 nm at standard temperature and pressure), and all 1 Hz data (grey dots) from ATom UT and ACCLIP are examined. The red box on panels a, b, c indicates O_3 and H_2O concentrations classifying airmasses as stratospheric air instructions from Zhang et al (see Methods), while the blue box in the same panels is the Zhang et al definition of convective outflow (COF). Panels a and b examine O_3 and H_2O alone, while panels c and d split the

ACCLIP observations by low and high CO, with high CO indicating COF. Panels e and f, show the same observations as a function of temperature and H_2O.

I

One major conclusion of the paper is that newly formed particles grow and increase the concentration of accumulation mode particles. One key piece of evidence is that accumulation mode concentration increases with the time since convection. However, because background air typically contains substantially more accumulation-mode particles than fresh convective outflow, this trend could largely reflect entrainment of background air rather than in-situ growth.

We agree that entrainment of background air can increase the accumulation mode number concentration after convection and for the first three days after convection, N60 remains lower than the background concentration (Fig. 2a). However, N60 peaks at 8-10 days since convection and then decreases towards the background air concentration (Fig. 2a), which cannot be explained by entrainment alone. The dilution effect is explicitly taken into account through the ΔN/ΔCO ratio in Fig. 10a,b. In the absence of other processes than dilution, this ratio would remain constant, as indicated also in Supplementary Methods (SM1). The increase in ΔN60/ΔCO with time since convection in Fig. 10b is direct evidence of N60 production in the convective outflow. Furthermore, we direct the reviewer to the increase and subsequent decrease with time of the Aitken mode number concentration, which could not be explained by entrainment, and therefore is evidence of particles growing through this size range and into the accumulation mode. This is demonstrated in Fig. 4 and discussed in the original text now at lines 192-200.

Furthermore, if growth of the new particles contributes substantially to the accumulation mode concentration, one would expect higher accumulation mode concentrations in outflows with CO > 150 ppbv than in those with CO < 150 ppbv. However, Fig. 3c appears to show the opposite.

We thank the reviewer for pointing out this discrepancy. We investigated the trend in accumulation mode number concentration with respect to CO using 1Hz observations, and including RF08 which saw the highest observed CO volume mixing ratios. The original fig. 3 used the 30s inverted observations, and had to exclude RF08 in the accumulation since the UHSAS was not operational on that flight. This is included in the new ED figure 6c and shows that the accumulation mode number concentration decreases with CO volume mixing ratio. Since CO mass maxing ratio decreases with time since convection (Fig 2d), this fits with the expected growth of newly formed particles over time.

Minor comments:

Figure 2 shows that the NPF fraction within two days of convection is ~80%, substantially higher than those after two days (e.g., mostly 20-30%). What proportion of all measurements fall within the 0-2 day window? That could provide insights into the relative importance of NPF

initiated in convective outflow versus NPF occurring >2 days later, potentially via different mechanisms. For example, if 20% of samples are within 0–2 days and 80% are >2 days, for a first-order estimate, the contributions to overall NPF incidence would be comparable—0.20×0.80 ≈ 0.16 for outflow-related NPF versus 0.80×0.25 ≈ 0.20 for NPF possibly via different mechanisms.

This is a nice way to consider the NPF events that we had not previously included. 13% of the data fall with the 0-2 day window (see below), in agreement with Fig 2d of Smith et al. 2025. Therefore, of the observed NPF 0.13x0.8≈0.1 outflow related NPF and 0.87*0.25≈0.23 possibly different mechanism. So relative importance of NPF events – we see about twice as much nucleation in total after 2 days than before. But the nucleation occurring within 2 days of convection also produces higher number concentrations. We also need to consider that the ACCLIP flights were not directly over the core of the monsoon subsystems, and this will bias our sampling (especially considering the EASM) towards longer times since convection. Therefore, considering the ASM as a regional system, this analysis does not change the major conclusions presented in the manuscript. Therefore, and given the inclusion of new modelling to better examine the influence of NPF, we feel this analysis would not add significant value to the manuscript.

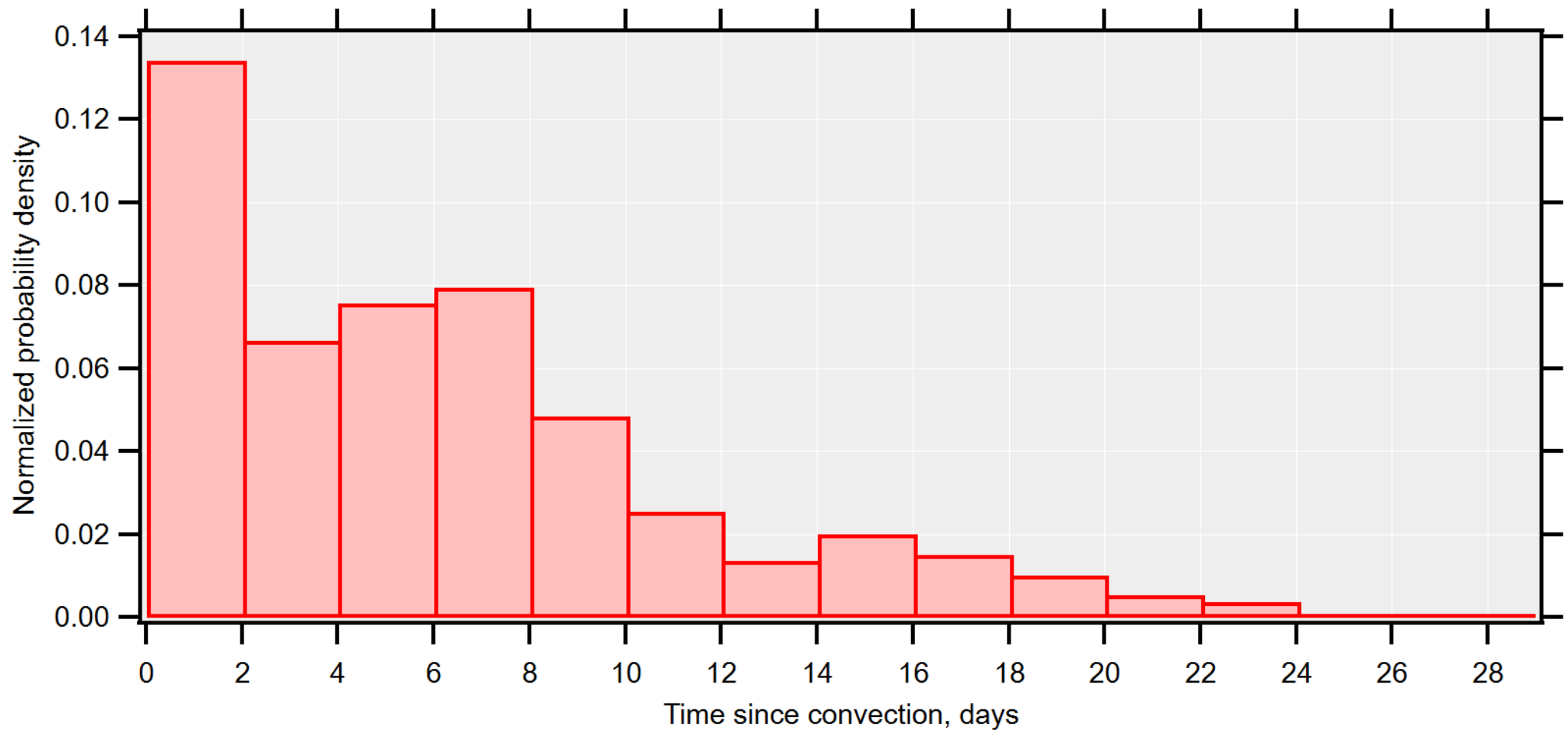

Response to Reviewers Figure 2. Histogram of time since convection of all ACCLIP observations included in this analysis.

The authors assess the impact of lofted pollution on particle formation and growth by comparing particle concentrations in ASM outflows to those in remote tropical convective outflows. While a higher particle growth rate in ASM outflows is very plausible, elevated particle concentrations alone do not demonstrate faster growth, especially for the growth to Aitken and accumulation mode size ranges. Increased Aitken and accumulation concentrations could be due to higher initial new particle concentration. Given the higher particle concentration and thus condensation sink, the concentration of condensation species and thus the growth rate are not necessarily higher in lofted pollution with elevated precursor levels.

We address this through modelling of nucleation as described above in the response to review 1 and below in the response to reviewer 3.

It appears that Extended Data Fig. 1 is not referenced in the text.

We thank the reviewer for catching this and have removed the figure accordingly.

Fig. 3. I would suggest including SO2 mixing ratios observed in different outflows if possible. It would help illustrate the impact of the key precursor.

We have included SO2 as suggested (the figure is now included in ED Fig 6), though note the limitation that SO2 on ACCLIP was only measured at the highest altitudes. We discuss this in the text in lines 174-187 in the context of nucleation mechanisms and the new model results as follows:

"While most UT observations show a paucity of ammonia[35], the lofting of polluted BL air makes ASM convective outflow a unique chemical environment[28,36,37]. Accumulation mode particles observed during ACCLIP are rich in ammonium and nitrate (Extended Data Fig. 9), in agreement with observations from StratoClim[29] and modelling studies[34]. A fast nucleation and growth mechanism involving ammonia, nitric acid and sulphuric acid has been proposed from laboratory experiments and modelling as possible in the upper troposphere and lower stratosphere (UTLS), particularly in ASM outflow, resulting in ammonium-nitrate particles [29,34,39-42]. This is consistent with the fast nucleation and composition demonstrated above and produces vertical profiles of particle number concentrations consistent with those observed during ACCLIP (Extended Data Fig 5). Aerosol composition measurements within the Asian Tropical Aerosol Layer (ATAL)[43] also show an abundance of organics[29] (Extended Data Fig. 9), suggesting these may also contribute to the nucleation or growth, similarly to in remote tropical convection[17]."

Line 119 New particle formation and growth can substantially increase the condensation sink. The condensation sink shown in Fig. 2c is mostly after the NPF, particularly for samples within two days of convection. Therefore, the variation shown in Fig. 2c does not necessarily prove condensation sink (prior to the onset of NPF) is not a controlling factor.

We agree with the reviewer that we cannot assess the condensation sink prior to the onset of NPF since observations at the shortest times from convection already show evidence of NPF. However, the condensation sink during NPF, even if elevated by newly formed particles, could still shut off nucleation, or limit the growth of particles beyond initial clusters. The growth of particles beyond cluster sizes there does still imply that it is not a uniquely low condensation sink that leads to the elevated particle concentrations in ASM outflow.

We have modified the text at lines 164-167 to include discussion of SO2 and clarify discussion of the condensation sink as follows:

"The ASM outflow condensation sink and SO_2 mass mixing ratio both show an initial maximum immediately after convection, coincident with the highest number concentrations of newly

formed particles (Fig. 2). This suggests that newly formed particles themselves limit the rate of new particle formation within this system."

Line 151: redundant words.

We thank the reviewer for catching this and have removed the redundant words accordingly (now line 205).

Line 53 of Supplementary Information: "SM section 2" instead of "SM section 4".

We thank the reviewer for catching this and have corrected it accordingly.

Reviewer #3 (Remarks to the Author):

Williamson et al. (2025) present an analysis of particle number concentrations measured during the ACCLIP field campaign. By comparing ACCLIP data with previous campaigns (ATom and StratoClim), the study posits that pollution lofted by the Asian Summer Monsoon (ASM) enhances nucleation in the convective outflow, with particles growing to CCN-relevant sizes within approximately one week.
While the topic is relevant, the manuscript in its current form does not appear to constitute a substantial scientific advance. The enhancement of aerosols and nucleation in the upper troposphere, particularly within the tropics and the ASM, has been extensively documented in prior field campaigns, modeling studies, and laboratory work—many of which are cited by the authors. The potential transport of these pollutant aerosols to lower altitudes and their subsequent contribution to cloud formation is an interesting concept. However, the authors support this claim with speculation rather than quantitative analysis. Consequently, I cannot recommend the manuscript for publication in Nature Communications in its present state.

Line 62: Please define what constitutes a "statistically significant" difference in number concentrations.

Statistical significance is defined in the methods, and we refer to that on line 75. We have now clarified the text discussing this (lines 75-79) so that it read as follows:

"NPF observed during ACCLIP occurred in around 80% of measured airmasses within 2 days since convection, but rapidly becomes less frequent afterwards, with NPF events occurring in only 20-30% of observations sampled more than 2 days since convection (Fig. 2b). Similarly, peak concentrations from NPF observed during StratoClim mostly occurred within 3 days after convection[23]."

Lines 86-89: This analysis is confusing. Please clarify how the conclusion regarding "Time since convection" was reached. Furthermore, what is the major difference between the EASM and SASM that underpins the comparison between ACCLIP and StratoClim? Is it merely that the SASM region is closer to the StratoClim measurement location? Finally, the phrase "this phenomenon" is vague; what is explicitly being suggested for the West African monsoon?

We agree that this discussion was confusing and relied too heavily on evidence presented only later in the paper. We have therefore modified the discussion of EASM, SASM and stratoclim in number of places, and have removed the reference to West Africa entirely. Conclusions regarding the importance of time since convection are left for the later discussion of Fig. 2. The changes to the text are as follows:

Line 51: deleted reference to the different ASM subsystems – this is fully explained later

Line 96-110, the text that was previously confusing at line 86-90 has been changed to:

"Over 50% of EASM convective outflow sampled during ACCLIP was within 1 day after convection, while 50% of the SASM outflow was sampled more than 6 days after convection (Extended Data Fig. 4a). ACCLIP observations show evidence of NPF in both EASM and SASM subsystems (Extended Data Fig. 4). We see broad agreement between ACCLIP and StratoClim observations of the total particle number concentration in the UT, with the median number concentrations peaking around 360 K potential temperature (Fig. 1). When ACCLIP observations are split by contribution from EASM and SASM convection, we see closer agreement in median total number concentration between 350-370 K potential temperature for EASM ACCLIP observations and total StratoClim (SASM) observations, despite the StratoClim observed CO mixing ratios being closer to those observed from SASM outflow on ACCLIP (Fig. 1b). The similarities in particle number concentrations in the first few days after convection indicate that NPF is occurring over the entire ASM convective region."

We also clarify the text referring to the subregions in lines 91-95 as follows:

"ACCLIP observations sampled two subsystems of the ASM[25]: the East Asian Summer Monsoon (EASM) which has its centre of activity along the East Asia subtropical front[27], and the South Asian Summer Monsoon (SASM) which has its centre of activity along the Monsoon Trough originating over Nepal and northern India and corresponds to the subsystem primarily observed during StratoClim[23]."

Analysis regarding Fig.3:
The comparison between the tropical UT and the ASM seems problematic. The tropical UT data uses a "before 10-day since convection" criterion, while the timeframe for the ASM data is unclear (3 days?). As demonstrated in Fig. 2, number concentrations are highly sensitive to this timing. Additionally, the dominant aerosol composition in the marine-influenced tropical UT likely differs from that in the pollutant-rich ASM. The observed differences in number concentration could therefore be attributed to various factors, including nucleation pathways, convection strength, and pre-existing particle concentrations and composition.
In the comparison between polluted and less polluted ASM outflow, nucleation mode particle number (N) is higher in more polluted air, yet accumulation mode N is lower near the cloud-top. What is the implication of this inverse relationship?

We agree that the comparison of tropical UT and ASM outflow was not sufficient evidence for the role of pollution in ASM NPF and have therefore done targeted modelling sensitivity of ASM NPF to better elucidate the role of NPF and precursors. The old figure 3 is therefore replaced

with these model outputs, and discussions included in the main text and methods as discuss above in the responses to reviewers 1 and 2. We have removed the comparison of tropical UT and ASM outflow from the manuscript, except for the bulk chemical composition presented in ED Fig. 9, which served to highlight the high concentration of nitrate and ammonium in ASM outflow.

We have included a more detailed examination of the relationship between CO and nucleation and accumulation mode number concentrations in the new ED Fig. 6, which is referenced in the following places:

Lines 126-127

"ACCLIP observations show a strong correlation between observed particle number concentrations and CO volume mixing ratio (Extended Data Fig. 6a)."

Lines 174-175

"Particle number concentrations observed on ACCLIP are not as highly correlated with SO_2 as they are with CO (Extended Data Fig. 6)."

Lines 211-214

"The magnitude of the accumulation mode number concentration increases with time since convection (Fig. 4b) and is inversely correlated with CO (Extended Data Fig. 6c), suggesting that many of these accumulation mode aerosols grow from the newly formed particles."

Lines 116-121: The conclusion that the condensation sink (CS) is not a controlling factor for new particle formation (NPF) requires further elaboration. Figure 2 shows that both nucleation mode particle number (Nd) and CS peak simultaneously within the first day, which seems to suggest a potential relationship rather than its absence.

We agree and have elaborated further as discuss above as part of our response to your comment on the importance of time since convection.

Given that the cited field campaigns measured aerosol composition and likely precursor gases, it is unclear why the authors did not leverage this data to evaluate specific nucleation pathways (e.g., involving organics or other compounds). Instead, the discussion of mechanisms (Lines 122-134) remains largely speculative. A quantitative assessment of the role of boundary layer pollutants—which is the defining feature of the ASM—is crucial yet missing from this analysis.

We agree that direct observations of precursor gases would provide excellent quantitative evaluation of nucleation mechanisms. Sadly, the key species such as sulphuric acid and ammonia were not measured on ACCLIP and ATom. SO2 was measured on ACCLIP at the highest altitudes and is included as discussed above. Since SO2 observations alone are therefore insufficient to understand the nucleation mechanisms, we instead address the concerns of speculative discussion of with additional targeted modelling as discussed above.

The abstract makes several claims regarding the regional climatic and air quality impacts of aerosols in the ASM upper troposphere. These assertions appear speculative, as they are not supported by quantitative analysis or modeling within the manuscript. To strengthen the abstract and ensure it accurately reflects the paper's content, I recommend either removing these speculative impact statements or substantially bolstering them with data-driven evidence in the main text.

We thank the reviewer for this helpful comment. We agree that the original abstract contained statements about regional climate and air-quality impacts that were speculative relative to the analyses shown.

As also outlined in our response to Reviewer 1, we added a targeted EMAC sensitivity analysis to the main text that quantifies the influence of precursor emissions on upper-tropospheric particle abundance and AOD during the ACCLIP campaign period (Fig. 3 and Extended Data Fig. 5). In line with the reviewer's comment we explicitly present these results as sensitivity evidence rather than a radiative-forcing or air-quality assessment. The abstract now focuses solely on supported conclusions.

To address this point, we have:

- Removed general claims about climate and air-quality impacts from the abstract
- Revised the abstract to focus only on what is directly supported by observations and the sensitivity simulations added in the revision (UT ultrafine particle enhancement and UT AOD response during ACCLIP)
- Included quantitative numerical model evidence in the main text demonstrating the sensitivity of UT aerosol loading to precursor emissions during the ACCLIP period (Fig. 3)

We have revised the abstract to read:

"The Asian Summer Monsoon (ASM) efficiently lofts air from surface areas with high levels of pollution to the upper troposphere and lower stratosphere (UTLS), with important implications for aerosol chemistry and climate interactions. In-situ observations of aerosol size distributions and trace gases combined with targeted modeling show that new particle formation occurs in outflow from ASM convection across the entire ASM region, and that pollutants transported from the surface increase the concentrations of particles formed aloft. These newly formed particles are grow to sizes at which they have potential to enhance scattering of radiation, influence cirrus cloud properties and act as cloud condensation nuclei after descending to lower levels."

In line with this, we have also revised the non-technical summary to read:

"Deep storms during the Asian Summer Monsoon loft polluted air from the surface to 12-18 km altitudes. Observations from aircraft combined with modeling show that the pollutants increase the number of aerosol particles that form at high altitude, and that these particles grow over a period of weeks to sizes where they have potential to impact climate by scattering sunlight and changing cloud properties. Many of these particles stay below the stratosphere and descend over time. Studies concerning the impacts of Asian summer monsoon on

climate often focus on the stratosphere, but this study shows it is important also to consider aerosol impacts below the stratosphere."

To integrate this revision into the main manuscript, we have revised the introductory paragraph, which now accurately reflects the scope and evidence in the manuscript and avoids speculative impact language. Lines 41-46 now read:

"We show that pollution lofted by the Asian Summer Monsoon enhances new particle formation in convective outflow, and that the resulting particles grow to sizes large enough to influence climate. Targeted Earth-system model sensitivity simulations show that new particle formation and precursor emissions modulate the UT particle abundances and associated UT AOD during the campaign period."

We have also made minor wording changing in the introductory paragraph to make it clearer and less speculative as follows:

Line 26: replaced "highly polluted" with "polluted"

Line 30: clarified "outflow" to "convective outflow"

Line 30: changed "Tropical upper troposphere new particle formation" to "Upper tropospheric new particle formation"

Line 40: changed "climatic influence of these particles" to "influence of these particles on climate"

Reviewer #4 (Remarks to the Author):
I co-reviewed this manuscript with one of the reviewers who provided the listed reports. This is part of the Nature Communications initiative to facilitate training in peer review and to provide appropriate recognition for Early Career Researchers who co-review manuscripts.

To properly integrate the changes mentioned above we have made also the following modifications:

- New coauthors Theodoros Christoudias, Francesco D'Amato, Matthias Kohl, Andrea Pozzer, Silvia Viciani and Christos Xenofontos have been added to the author list along with the new institutes Climate and Atmosphere Research Center, The Cyprus Institute, Nicosia 1645, Cyprus and National Research Council—National Institute of Optics (CNR-INO), Sesto Fiorentino, Italy. The coauthor order and institute number has been modified accordingly.
- In the abstract the relative qualification of the ASM boundary layer as "highly" polluted has been removed (new line 26) and qualification of ASM outflow to ASM <u>convective</u> outflow has been made throughout.
- Reference 22 changed from accepted to fully published version (line 327)
  *Ueyama, R. et al. On the Fate of Aerosols Produced by New Particle Formation in the 327 Upper Troposphere and Lower Stratosphere. Geophysical Research Letters **52**, 328 e2025GL116860, doi:https://doi.org/10.1029/2025GL116860 (2025).*

- Reference 27 changed from an earlier work discussing the ACCLIP campaign modelling dry run to the ACCLIP overview paper that was published after our initial submission. The new reference in line 346 is:
  Pan, L. L., Atlas, E. L., Newman, P. A., Thornberry, T., Jucks, K. W., Toon, O. B., Randel, W. J., Liang, Q., Kinnison, D. E., Ueyama, R., Bresch, J. F., Honomichl, S. B., Smith, W. P., Hornbrook, R. S., Ziemba, L., Fujiwara, M., Apel, E. C., Barucci, M., Bianchini, G., Brown, M., Bui, T. P., Campos, T., Chin, M., D'Amato, F., Dean-Day, J., Diskin, G., Franchin, A., Gurganus, C., Iraci, L. T., Kim, J., Koo, J.-H., Lait, L. R., Lesko, K., Podolske, J. R., Rollins, A., Sakai, T., Shiraishi, K., Treadaway, V., Viciani, S., Waxman, E. (2025). The Asian summer monsoon chemical and climate impact project (ACCLIP): An overview. Journal of Geophysical Research: Atmospheres, 130, e2025JD044417. https://doi.org/10.1029/2025JD044417
- Line 601 "based on ERA5 reanalysis data[84]" corrected to "based on ERA5 reanalysis[84]"
- Minor grammar/wording improvements throughout, all marked in the tracked version of the manuscript
- Ref 34 has been corrected from referencing the dataset "Nault, B. A. *et al.* (ORNL Distributed Active Archive Center, 2022)." To referencing the paper Nault, B. A. *et al.* Chemical transport models often underestimate inorganic aerosol acidity in remote regions of the atmosphere. *Communications Earth & Environment* **2**, 93, doi:10.1038/s43247-021-00164-0 (2021).
- New refs 56 and 57 added to describe condensations sink calculation lines 716-719
- New acknowledgements added at line 840 as follows:
  "The COLD2 deployment was funded by the UE 7th FP StratoClim Project, grant agreement no. 603557. TC, AP, and CX acknowledge funding from the European Union (EU)'s Horizon 2020 research and innovation programme under grant agreement no. 856612 (EMME-CARE); EU Marie Skłodowska-Curie Actions Doctoral Network CLOUD-DOC 101073026; Horizon Europe project FOCI (no. 101056783). WPS was supported under grant NSF AGS-1853929 and NASA Grant 80NSSC24K0706."

23.03.2026

Response to reviewer comments on "Upper Troposphere Aerosol Formation Enhanced by Monsoon Lofted Pollution" by Williamson et al.

We thank the reviewers for their comments, which we address point-by-point here below and in the revised manuscript with changes tracked. We have revised our definitions of stratospheric air intrusions to better suit the polluted convective region examined here and included a quantitative evaluation of the relative impacts for SAI and pollution on NPF in ASM outflow. We have extended our modelling studies to better evaluate the impact of NPF of CCN and adjusted our language on speculative mechanisms and impacts. Furthermore, we include additional observational evidence and model studies supporting both the role of pollution in ASM outflow NPF and the proposed nucleation mechanism.

We format the following text with the reviewer remarks in black, and responses from Williamson et al. in blue. Quoted text from the manuscript is given in italics. References are numbered separately for this document and the manuscript.

Reviewer #1 (Remarks to the Author):

The authors have made substantial revisions and supplements in response to the initial reviewer comments, including adding targeted model simulations, improving data presentation, and supplementing methodological descriptions, which have significantly enhanced the scientific rigor and persuasiveness of the study. The integration of multi-campaign observations, trajectory modeling, and EMAC model sensitivity simulations provides a comprehensive framework to test the core hypothesis. The findings, namely that ASM-lofted pollutants enhance UT NPF and that the resulting particles grow to climate-relevant sizes, are timely and contribute to filling the gap in understanding anthropogenic impacts on UTLS aerosol processes. However, several key issues related to mechanism clarity, data limitations, and quantitative support for conclusions need to be addressed to strengthen the manuscript's rigor and impact.

1. The study uses remote convection over the tropical Pacific/Atlantic (from the ATom mission) as a "low-pollution control." However, this region fundamentally differs from the ASM in meteorological background and natural source emissions. These factors themselves can affect NPF efficiency, not solely pollution levels. Without controlling for non-pollution variables via statistical methods (e.g., meteorological factor matching), the study cannot definitively attribute differences in particle concentrations to pollution lofting.

We agree that we are unable to control for the fundamental differences between ASM and tropical convection. In the first round of revisions, we removed the ATom comparison that had had been used to demonstrate the influence of pollution. What remains from ATom is the chemical composition comparison in ED fig 8a, which is only from larger particles. The demonstrated difference in ammonium and nitrate concentrations that make it into accumulation mode particles illustrates well different chemical environments found in the UT.

2. Observations from ACCLIP (2022), StratoClim (2017), and ATom (2016–2018) span multiple years. Interannual variations in ASM intensity and pollutant emissions may have biased results, with no assessment of temporal variability effects.

We thank the reviewer for bringing up the important issue of interannual ASM variability. We have added the following text in the supplementary discussion:

In past studies, the convective transport to the ASM anticyclone was found to be primarily characterized by convective uplift from the southern flank of the Tibetan Plateau (e.g., [1-3]).This subsystem of the ASM is also known as the South Asian Summer Monsoon (SASM). This subsystem was found to be the dominant vertical transport pathway during the StratoClim mission 2017[4]. In 2022, however, the Asian Summer Monsoon (ASM) region experienced anomalous large-scale dynamical conditions during late summer, leading to extreme weather events in East Asia, such as the record-breaking rainfall in early August[5,6]. These conditions were associated with enhanced convective activity over parts of northern China and the Yellow Sea, which were linked to a northward migration of the East Asian Subtropical Front[7]. This migration was induced by the triple-dip La Niña event of 2022[7,8]. During the ACCLIP campaign, the sampling location over the western Pacific region coincided with deep convection over East Asia in August 2022, facilitating the sampling of East Asian Summer Monsoon (EASM) transport in addition to SASM air. The chemical characteristics of the ASM anticyclone in the UTLS is expected to differ between both sub-systems due to different environmental forcings and emissions. Indeed, Smith, et al. [9] found a higher abundance of pollutants in the UTLS from EASM convection compared to convection from the SASM.

We now refer to this in the main text at line 49 as follows:

The observations show similar spatial extent and number concentrations in both ACCLIP and StratoClim despite substantial interannual variation in the ASM and sampling locations within the region (Supplementary Discussion section 2).

To make the subsequent text flow better we have then modified text at line 52 from "*This coincides with …*" to "*The peak in number concentration coincides with …*".

We analyse convective outflow from two different regions of the main anticyclone on ACCLIP: EASM and SASM as shown in ED Fig. 1a. Because of the location of the StratoClim mission it sampled predominantly SASM as noted in line 91 (see also Eppers et al. 2025 Fig. 1).

The comparison in fig. 1 is therefore made between StatoClim SASM outflow observations, and ACCLIP divided into SASM and EASM outflow. SASM outflow was mostly measured within a few days of convection of StratoClim, but after about 5 -10 days after convection on ACCLIP, whereas EASM outflow on ACCLIP was frequently measured in the first days after convection.

Regarding ATom, we removed the comparison of aerosol number concentrations and mixing ratios of CO and SO2 in the first round of revisions, leaving only the comparison of accumulation mode aerosol chemical composition in supplementary figure 8 to illustrate the abundance of nitrate and ammonium in accumulation mode aerosol in the ASM region.

3. The study defines NPF based on statistical significance of particle concentrations between 6–8 nm but fails to exclude interference from non-NPF processes (e.g., existing particle fragmentation, aerosol coagulation redistribution). While stratospheric air intrusions (SAI) are not the primary driver, this is only inferred indirectly via CO concentrations. Quantitative correlation analysis between SAI signature factors and NPF events is lacking, leaving residual SAI contributions unaddressed.

The ACCLIP observations excluded in-cloud observations to avoid the possibility of ice particles fragmenting on the inlet leading to artificially elevated number concentrations. The StratoClim observations also excluded this possibility as discussed in [10,11].

Regarding aerosol coagulation redistribution, the GMXe box modelling (ED Fig. 3) analyses the evolution of the size distribution in the first hours after convection. It shows the strong role of condensation and self-coagulation of newly formed particles in initial particle growth and decreasing number concentration, and very little influence on particles larger than 40 nm in the first 3 days after convection. This therefore supports our conclusion of aerosol nucleation in convective outflow.

We refer to this more clearly now at lines 155-157 as follows:

Box modelling indicates high supersaturation of condensable vapours driving efficient growth of newly formed particles, and high self-coagulation of the smallest particles (Extended Data Fig. 3).

We include more detailed analysis of the influence of SAI as follows:

We used threshold mixing ratios of water vapour, ozone and CO (400 ppmv, 70 ppbv, and 100 ppbv respectively) to classify airmasses by influence of SAI, COF and pollution, and analyse only high altitude airmasses (above 7 km). NPF is only analysed for airmass classifications where at least 30 minutes of sampling were made over the entire campaign. The majority of high-altitude flight time was spent in SAI influenced airmasses (ED Fig. 7 bf). Of these, around 50% of the polluted airmasses showed statistically significant NPF compared with around 10% of the clean airmasses. 30% of the polluted SAI airmasses showed statistically significant NPF with high number concentrations, compared with only 0.5% of the clean SAI airmasses. Less than 10 minutes of sampling time was spent in convective outflow (COF) airmasses without stratospheric influence (1249 s polluted, 1050 s clean), rendering the NPF statistics in this region insignificant. The high H2O high O3 region was almost all polluted (1176 s clean, 6758 s polluted) with 45% of the polluted airmasses showing NPF (nearly 20% NPF with very high number concentrations). The low H2O low O3 region was also mostly polluted (815 s clean, 5170 s polluted). Over 40% of the polluted airmasses showing NPF. This shows the heavy influence of stratospheric air intrusions in the sampled region, and that the influence of SAI on NPF cannot be ruled out. It also shows that pollution is highly associated with the occurrence of NPF, and more highly associated with the occurrence of NPF with high number concentrations.

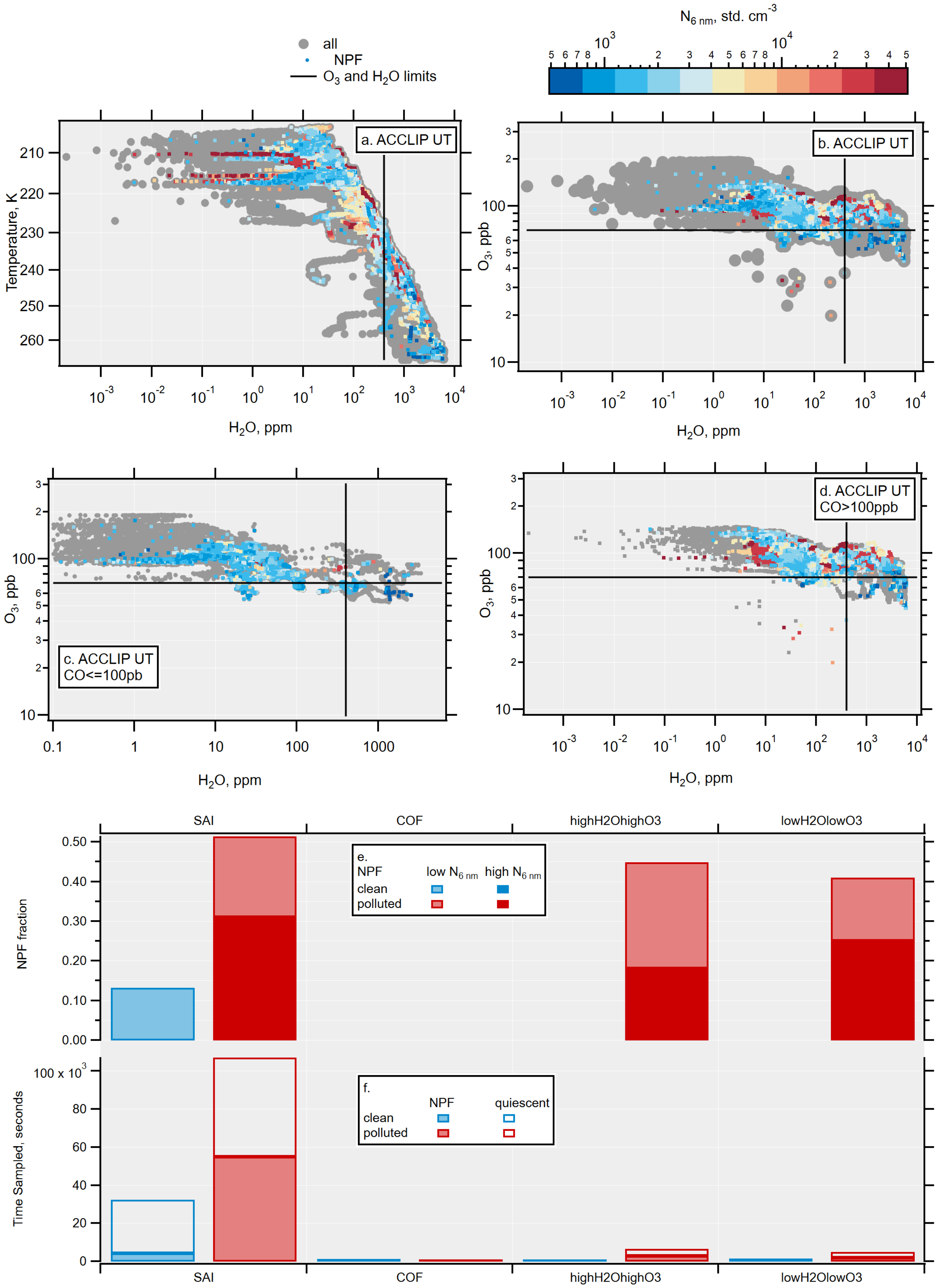

Extended Data Figure 7. Examining the influence of stratospheric air intrusions (SAI) and temperature on NPF in ASM with ACCLIP observations. 4 high altitude (above 7 km) regions defined by threshold

mixing ratios of water vapour (H2O) and ozone (O3), and quantified as polluted or clean by threshold CO mixing ratios. Threshold values are 100 ppbv CO, 400 ppmv H2O, and 70 ppbv O3. SAI is classified as high O3, low H2O, and COF as low O3, high H2O following Zhang et al[90] (see Methods). 1 Hz data indicating NPF (coloured dots, with colours indicating the number concentration of particles greater than 6 nm at standard temperature and pressure), and all 1 Hz data (grey dots) from ACCLIP are examined. The back lines on panels a-d indicate threshold O_3 and H_2O mixing ratios, or convective outflow (COF: low O_3, high H_2O). a) temperature and H_2O distribution. b) O_3 and H_2O distributed for all data, c) low CO data, and d) high CO data. e) the fraction of clean (blue, low CO) and polluted (red, high CO) airmasses within each sampling region that showed statistically significant makers of NPF. Darker shading indicates NPF events with high number concentration (N6>=5000 cm-3 STP) and lighter shading indicates NPF events with low number concentrations (N6<5000 cm-3 STP). f) the total number of 1Hz observations made in clean (blue, low CO) and polluted (red, high CO) airmasses in each sampling region, with shaded bars indicating observations with statistically significant NPF and unshaded bars indicating no statistically significant NPF.

We have clarified our statement about SAI influence at lines 144-150 as follows:

Most of the airmasses sampled in ASM outflow on ACCLIP were somewhat influenced by stratospheric air intrusions (SAI, see Methods, Extended Data Fig. 7), and the mixing between descending stratospheric air and lofted pollution may contribute to the high particle concentration observed in ASM outflow as has been observed in less polluted convective regions [12]. Within SAI influenced airmasses, more frequent NPF and stronger NPF (high number concentrations) are associated with higher CO concentrations, supporting the conclusion that lofted pollution is the main NPF driver in ASM outflow (ED Fig. 7 e, f).

4. The EMAC model's simulation results are only compared with observations for small particle sizes (<10 nm), with no validation for climate-relevant critical sizes (accumulation mode >60 nm). Additionally, the model does not separate the contributions of anthropogenic vs. natural pollutants, failing to support the core conclusion that "anthropogenic pollution enhances NPF."

We thank the reviewer for catching the lack of clarity in the main text and figure caption regarding the EMAC-ACCLIP comparison and the sensitivity studies.

The comparison shown previously in ED Fig. 5 was for all particles between 5 and 1500 nm, which includes the accumulation mode. However, since this is dominated by smaller particles aloft, we have added panels to this figure separating out the nucleation and Aitken modes and the accumulation mode. We have also improved the EMAC simulations by taking model outputs along the ACCLIP flight tracks to provide a more direct comparison. This is shown in the figure below.

We also include new comparisons of the EMAC and observed chemical composition, as discussed in answer to point 5 below.

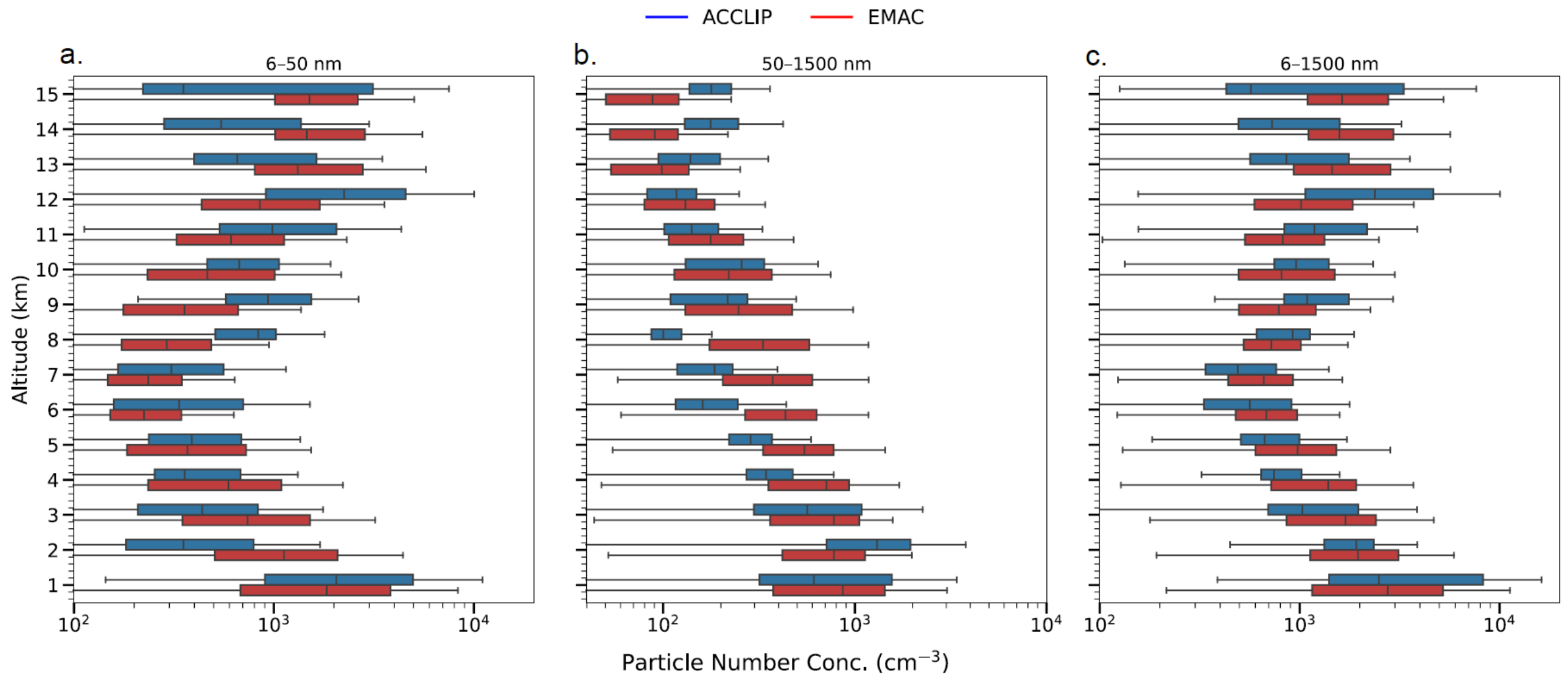

Extended Data Figure 5. Comparison of modeled particle number concentrations (EMAC; red) with aircraft observations from the ACCLIP campaign (blue) across 1–15 km altitude range. Boxplots compare the observed (ACCLIP) and simulated (EMAC) particle number concentrations in 1km vertical bins. The vertical line inside each box marks the median, the box spans the 25th–75th percentiles, and the whiskers indicate the full data range. Panels shows the total number concentrations between 6 and 50 nm (a), 50 and 1500 nm (b), 6 and 1500 nm (c)

We have modified the main text at lines 121-125 (as part of broader revision to this section) to now read:

Targeted simulations with the EMAC model (see Methods) predict substantial NPF activity in the upper troposphere over the ASM region consistent with the fast nucleation demonstrated above and vertical profiles of particle number concentrations consistent with those observed during ACCLIP (Fig. 3 and Extended Data Fig. 5 a). Sensitivity simulations further show that anthropogenic emissions of SO_2 and NH_3 strongly enhance NPF in the ASM UT (Fig. 3 a-c).

Regarding the separation of anthropogenic and natural emissions, we refer the reviewer to Fig. 3 where orange lines in panel a-c show simulation with anthropogenic emissions switched off and panel d shows the relative difference in AOD when anthropogenic emissions are switched off. This is described in the Fig. 3 caption at line 161 as *"a simulation without anthropogenic NO_x, SO_2, and NH_3 emissions (orange)"* and at line 165 as *"Relative difference in upper-tropospheric aerosol optical depth at 550 nm (AOD550) showing the change that occurs when anthropogenic NO_x, SO_2, and NH_3 emissions are included (relative to the simulation without anthropogenic emissions), averaged over 10–15 km"*. Panels a-c show the contribution of anthropogenic emissions to newly formed particles, larger particles, and nucleation rates respectively.

We have clarified the anthropogenic emissions in the main text at lines 124-125 to explicitly refer to SO2 and NH3 emissions as follows:

Sensitivity simulations further show that anthropogenic emissions of SO_2 and NH_3 strongly enhance NPF in the ASM UT (Fig. 3 a-c).

We also add the following text to the EMAC modelling section in the methods at lines 647-649:

Model data are sampled at the model grid boxes along the actual flight tracks, using the specific flight dates and times, to ensure the closest possible correspondence with the observed measurements. Model data are compared to all flights during ACCLIP.

5. The study proposes an NH3-HNO3-H2SO4 nucleation and growth mechanism but faces two critical inconsistencies: first, the correlation between SO2 and particle number concentrations is significantly weaker than that with CO, failing to confirm sulfuric acid as a key NPF precursor; second, high-resolution observations of NH3 in the upper troposphere over the ASM are lacking, with the mechanism relying solely on model inferences rather than direct observational support.

Regarding the hypothesised NH3-HNO3-H2SO4 nucleation and growth mechanism, we strength our evidence for this as follows:

- We failed previously to properly explain how the weak correlation of nucleation mode particles to SO2 compared to CO does not rule out this mechanism. The driver of the NH3-HNO3-H2SO4 nucleation in the UT is mostly NH3. SO2 and HNO3 are sufficiently available in the UT (both in tropospheric and stratospheric air), however, significant amounts of ammonia are needed to initiate the NPF (only available in tropospheric air). The parameterisation of the NH3-HNO3-H2SO4-nucleation rate is proportional to $[H2SO4]^3[HNO3]^2[NH3]^4$ [13], showing that ammonia is the most important precursor for this nucleation mechanism. The higher correlation with CO shows that the NPF driver originates at the surface.
- While we do not have direct measurements of NH3 mixing ratios, we present new outputs from the EMAC modelling demonstrating that with this mechanism the model represents well the observed aerosol composition and UT enhancement, including ammonium, nitrate and sulphate (new figure below). This agreement requires adequate representation of the precursor gases, and indicates that precursor gases for the nucleation mechanism are likely represented adequately, i. e. that ammonia strongly tends to the aerosol phase and ammonium reflects the encountered ammonia concentrations along the particles' trajectories.
- While an additional contribution of organic NPF is possible, the high concentrations of monoterpenes and isoprene needed for this are not expected in the ASM region and an organic contribution is not necessary to explain the enhanced particle number concentration as these are explained by NH3-HNO3-H2SO4 nucleation as shown in the EMAC modelling figures.

We have integrated the new modelling results and clearer explanation into the original text in the pollution section and restructured it to improve clarity. The revised text in lines 105-157 now reads:

Evidence of pollution lofted from the boundary layer (BL) enhancing NPF in ASM outflow is given using carbon monoxide (CO) as a tracer for pollution, (though it is not itself a condensable vapour for NPF[32,33]). ACCLIP and StratoClim observations show a strong correlation between observed particle number concentrations and CO mixing ratio (Extended Data Fig. 6a). Particle number concentrations are more weakly correlated with SO_2 (a precursor to H_2SO_4) than to CO (Extended Data Fig. 6b) indicating the influence of condensable vapours beyond H_2SO_4.

The most likely nucleation mechanism is synergistic NH_3-HNO_3-H_2SO_4 nucleation, a fast UTLS nucleation and growth mechanism proposed from laboratory experiments and modelling[13,14]. SO_2 and HNO_3 were both observed in ASM outflow on ACCLIP with sufficiently high mixing ratios for this mechanism (Extended Data Fig. 6). High mixing ratios of ammonia are present in ASM convective outflow[35,36]. The parameterisation of the NH_3-HNO_3-H_2SO_4-nucleation rate is proportional to $[H_2SO_4]^3[HNO_3]^2[NH_3]^4$ [13], showing that NH_3 is the main driver of the nucleation rate. The stronger correlation with CO than with SO_2 support a surface origin NPF driver and is consistent with the

assumption of the importance of NH_3. While SO_2 and HNO_3 are also present in the stratosphere (e.g. [15,16]), NH_3 is derived primarily from surface anthropogenic sources[17,18], and requires ASM convective lofting.

Targeted simulations with the EMAC model (see Methods) predict substantial NPF activity in the upper troposphere over the ASM region consistent with the fast nucleation demonstrated above and vertical profiles of particle number concentrations consistent with those observed during ACCLIP (Fig. 3 and Extended Data Fig. 5 a). Sensitivity simulations further show that anthropogenic emissions of SO_2 and NH_3 strongly enhance NPF in the ASM UT (Fig. 3 a-c).

NH_3-HNO_3-H_2SO_4 nucleation results in ammonium-nitrate particles[29,30,37-40]. This is consistent with the high concentrations of ammonium and nitrate observed in accumulation mode particles on ACCLIP (Extended Data Fig. 8, Extended Data Fig. 5d, e), and previously on StratoClim[28,29]. EMAC simulations with NH_3-HNO_3-H_2SO_4 nucleation and growth produce particles in ASM outflow that match the observed chemical composition well (Extended Data Fig. 5d-g). This agreement indicates adequate representation of the precursor gases in EMAC and therefore supports the modelled nucleation mechanism.

Prior modelling studies highlight the role of monsoon convection in lifting NH_3 into the UTLS. Xenofontos, et al.[30] used EMAC simulations over the South Asian monsoon to show that days with convection produce UT NPF rates that are one to two orders of magnitude higher than quiescent days, driven by NH_3 uplift. Xenofontos, et al. [31] extended this globally, finding that anthropogenic NH_3 emissions when combined with convective transport enhance UTLS NPF rates. Together these studies provide mechanistic context for the observation–model findings we present here.

Organics are also abundant in the observed larger particles in ASM outflow (Extended Data Fig. 8, Extended Data Fig. 5g) and may be involved in nucleation and/or growth, similarly to in remote tropical convection[17]. However, so far, no mechanisms have been proposed for this, and our model results show that nucleation involving organics is not required to explain the observed particle composition and concentrations.

Most of the airmasses sampled in ASM outflow on ACCLIP were somewhat influenced by stratospheric air intrusions (SAI, see Methods, Extended Data Fig. 7), and the mixing between descending stratospheric air and lofted pollution may contribute to the high particle concentration observed in ASM outflow as has been observed in less polluted convective regions [12]. Within SAI influenced airmasses, more frequent NPF and stronger NPF (high number concentrations) are associated with higher CO concentrations, supporting the conclusion that lofted pollution as the main NPF driver in ASM outflow (ED Fig. 7 e, f).

Alongside requiring sufficient supersaturation of condensable vapours, NPF is impeded by sinks for vapours and small particles as they condense onto or coagulate with other particles present (represented by the condensation sink). Both the ASM outflow condensation sink and SO_2 mixing ratio both show an initial maximum immediately after convection, coincident with the highest number concentrations of newly formed particles (Fig. 2). Box modelling indicates high supersaturation of condensable vapours driving efficient growth and high self-coagulation of the smallest newly formed particles (Extended Data Fig. 3). This suggests that newly formed particles themselves limit the rate of NPF within this system.

The EMAC-ERICA aerosol composition plots are included in Extended Data Fig. 5 are as shown below, with the included caption:

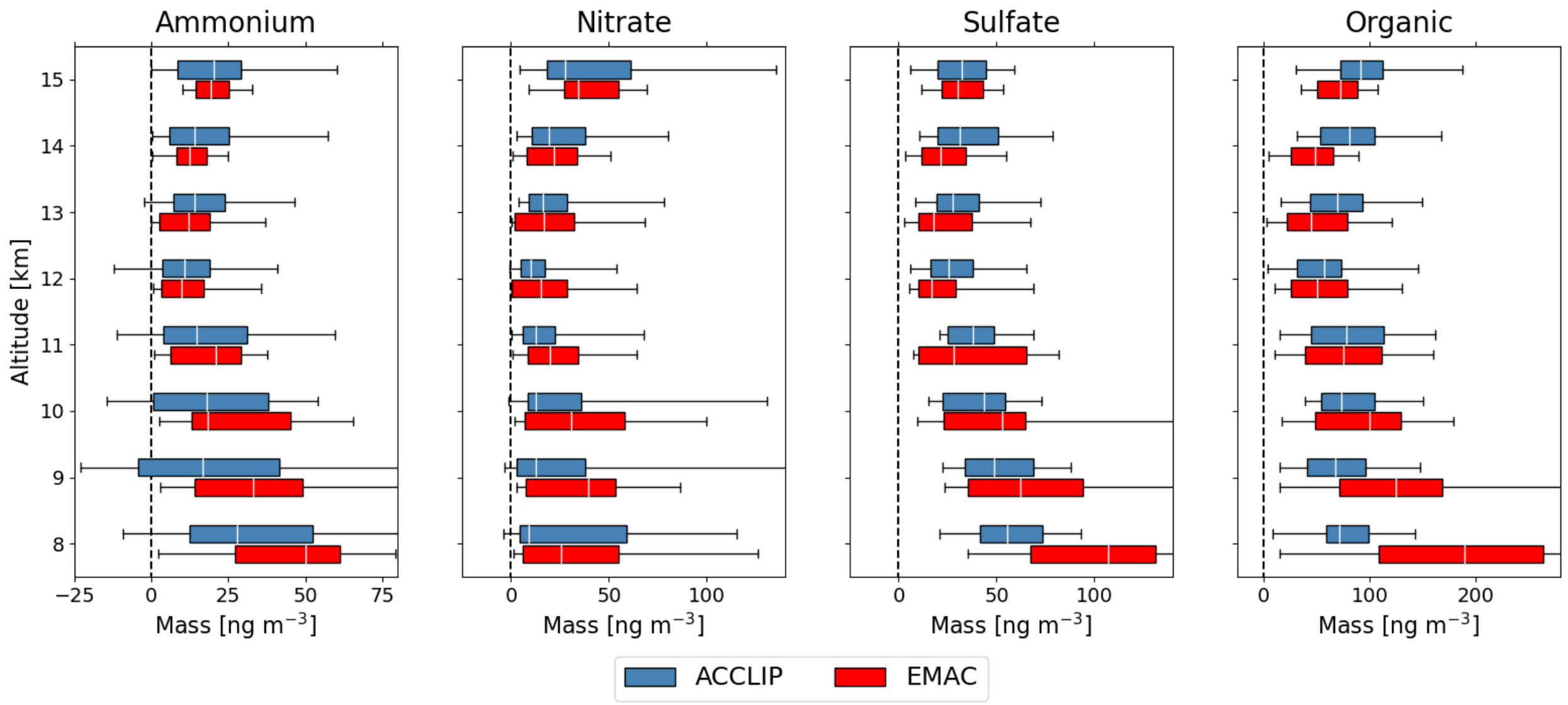

The mass concentration of ammonium (d), nitrate (e), sulphate (f) and organics (g) is similarly compared for particles between 120 and 3500 nm between EMAC simulations and ERICA observations.

The observed HNO3 mixing ratios are included in the new panel e of Extended Data Fig. 6 as shown below, with the included caption. The description of measurements from the CIMS instrument at lines 528-538 has been updated as follows to include details of the HNO3 measurements:

Sulphur Dioxide and Nitric Acid

Sulphur dioxide (SO_2) and nitric acid (HNO_3) weremeasured on the GV during ACCLIP by the Georgia Tech Chemical Ionization Mass Spectrometer (GT-CIMS) at high altitudes. SO_2 was calibrated frequently in flight by addition of isotopic $^{34}SO_2$ standard. The sensitivity of HNO_3 was examined relative to SO_2 in post-mission calibrations conducted under dew point conditions representative of those observed during ACCLIP. Background measurements were performed periodically by drawing ambient air through a scrubber containing glass wool impregnated with sodium carbonate.These measurements were made at 5s intervals with an estimated accuracy of 10% and 50% for SO_2 and HNO_3, respectively [19,20]. For this analysis they are expanded to have the same data value at 1Hz within each 5s measurement period, and no value outside those periods. GT-CIMS was not operational during ACCLIP 12th and 15th August.

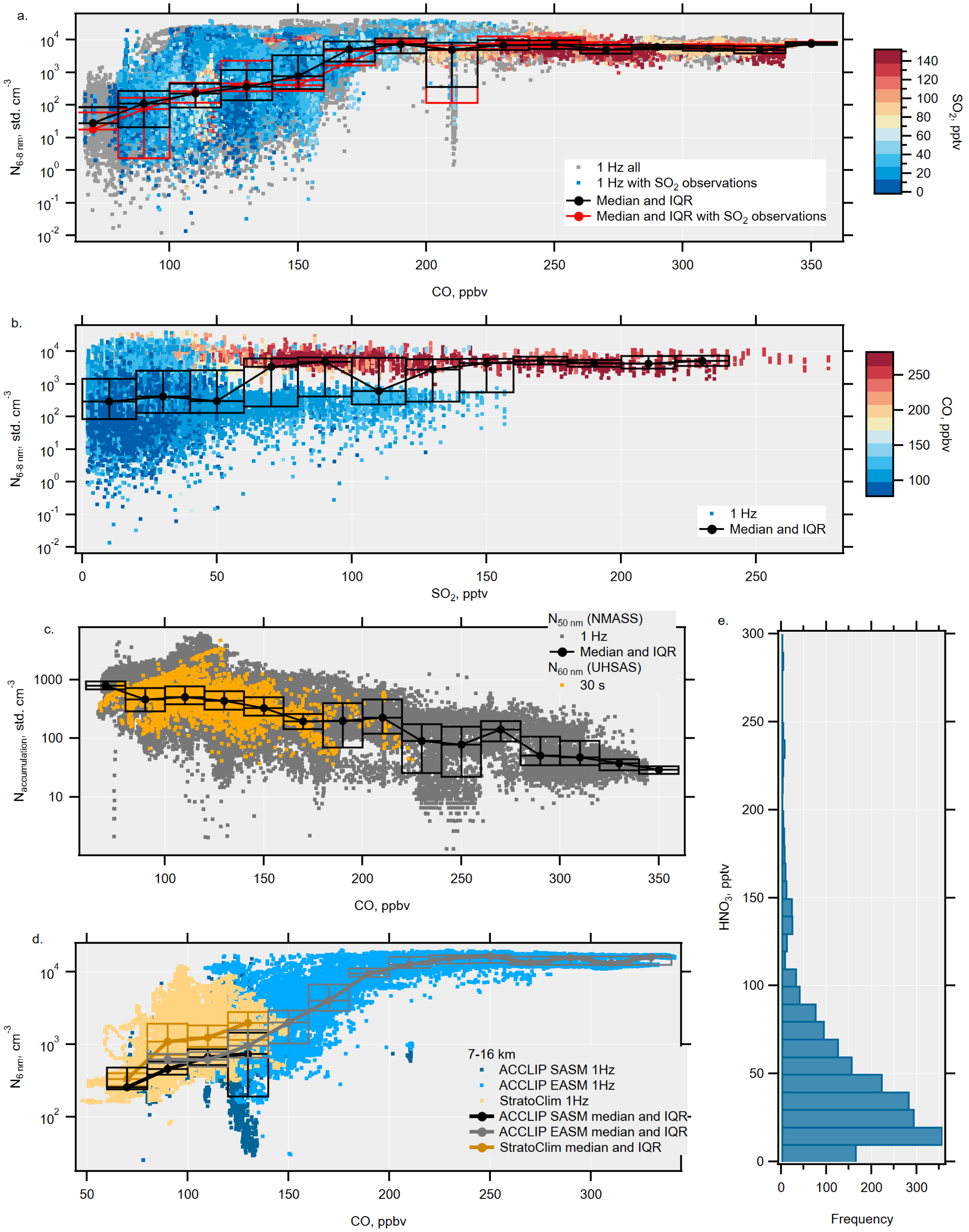

Extended Data Figure 6. Correlation of $N_{6\text{-}8\,nm}$ with CO (a) and SO_2 (b), with colours showing dependence on CO (a) and SO_2 (a) Coloured points are 1Hz observations, while black circles show the medians over a CO (a) or SO_2 (b) range given by the horizontal error bars. The IQR is given by vertical error bars. SO_2 observations are only at the highest altitudes, so (a) shows in grey points between 7 km

and the SO_2 observations. (b) shows in small grey dots data points identified as SAI. (c) shows the accumulation mode number concentration as a function of CO. Since the UHSAS was not functional on RF08, where the highest CO mixing ratios were observed, the inverted N60nm at 30s intervals including the UHSAS data and excluding RF08 is shown in yellow, and the N50nm from the 1Hz NMASS 50 nm channel is shown in grey. The median and IQR black boxes are calculated using N50nm. (d) shows the 1 hz observations together with the median and interquartile range (IQR) total number concentration above 6 nm between 7 and 16km as a function of CO from ACCLIP SASM and EASM (dark and light blue 1Hz black and grey statistics respectively) and StratoClim (light orange 1Hz, dark orange statistics). Statistics are only shown for CO bins with more than 5 minutes of data. (e) Histogram of HNO_3 mixing ratio measured on ACCLIP. All HNO_3 observations were made between 11.7 and 15.8 km altitude.

6. The conclusion claims particles influence climate via radiation scattering and cloud property modification but only provides relative changes in AOD (Fig. 3d) without quantitative radiative forcing calculations. Critical cloud microphysical parameters (e.g., CCN activity, cloud droplet number concentration) are neither observed nor simulated. Climate effects are merely qualitative inferences, lacking quantitative support.

We address this by including in ED Fig 5 CCN number concentration from the EMAC model, thereby showing model outputs directly related to aerosol radiation interactions (AOD in fig. 3 included in first revision of the manuscript) and aerosol cloud interactions (CCN). We look at CCN number concentration changes in the model between 10 and 14 km since both model and observations to reduce the influence particles formed in the boundary layer. Model results during and after the monsoon season are compared with and without anthropogenic gas-phase emissions to show the combined impact of anthropogenic emissions and convection.

This is referred to in the main text at lines 191-192 as:

At these sizes they are large enough to scatter radiation (Fig. 3g, Extended Data Fig. 10), impact cloud properties (Extended Data Fig. 5d-g), and provide surface area for heterogeneous chemistry[21].

The references that were previously used here as evidence for accumulation mode aerosol influencing cloud properties have been moved to line 200 as:

The trend indicated is that of particles forming very shortly after exiting the convective cloud and then growing over about a week to sizes where they have potential to act as cloud condensation nuclei (CCN) [13,14,21-24].

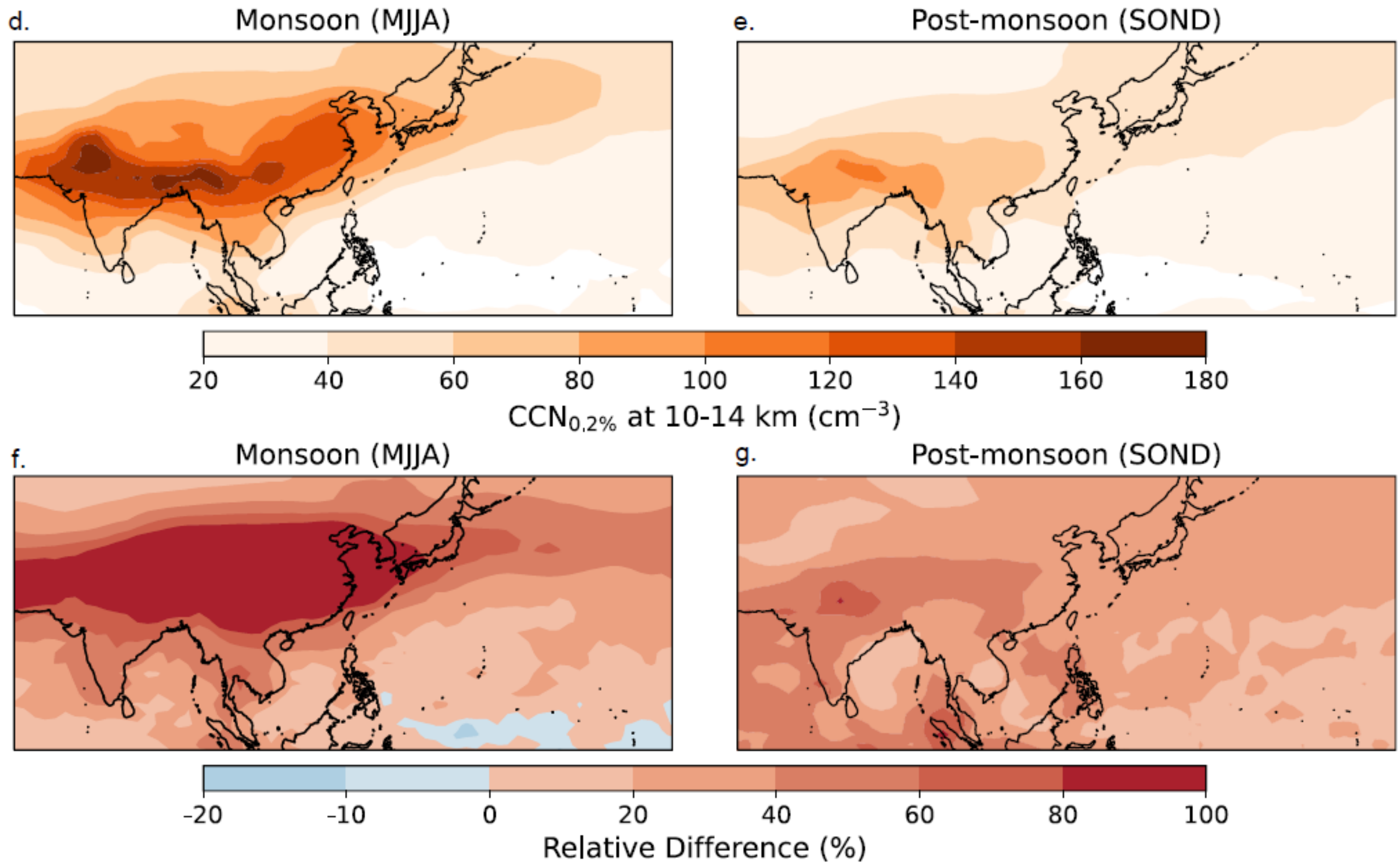

The new panels of ED Fig 5 are described in the figure caption as follows:

The simulated (EMAC) concentration of CCN between 10 and 14 nm altitude over the ASM region is shown during the ASM (d) and after the ASM (e), showing enhances CCN at cloud outflow altitudes during the monsoon. The relative difference in CCN number concentrations over the same region and altitude range between the default model and the model runs without anthropogenic SO_2 and NH_3 emissions and shown for the monsoon period (f) and post-monsoon period (g), showing the CCN enhancement due to lofted gas phase pollutants.

7. The study integrates observational data from ACCLIP (GV aircraft), StratoClim (M-55 aircraft), and ATom (DC-8 aircraft). Although it mentions "the same data processing methods," it does not explain the comparison results of calibration biases between instruments from different platforms. It is recommended to supplement consistency validation of cross-platform instruments to avoid instrument systematic errors affecting regional comparison conclusions.

The statement about "the same data processing methods" referred to only ATom and ACCLIP, since the same instrument was flown by the same operator and data were analysed using the same software. However, since ATom NPF observations were removed in the first round of revisions, this text was also removed.

Following discussion between ACCLIP and StratoClim instrument teams, we agree that an intercomparison of instrumentation across the two platforms would be an interesting addition to the literature, but that this a major work of its own. Instead, we note that both instruments have been calibrated and this is documented within the manuscript for ACCLIP (extended data figure 11) and in the cited literature for StratoClim. Since our comparison with StratoClim focuses on the total number concentration above 6 nm to show similar increases in particle number concentrations at cloud

outflow altitudes, we consider these separate calibrations sufficiently robust to uphold the conclusion.

8. ERICA observations show that accumulation mode particles are rich in ammonium and nitrate, but no comparison is made of particle chemical composition differences between the EASM and SASM regions. Given that the EASM is more polluted, supplementing this difference analysis can further support the "pollution-driven NPF" mechanism and explain NPF efficiency differences between different sub-monsoon systems.

We thank the reviewer for the suggestion to use the different levels of pollution sampled during ACCLIP and StratoClim to further investigate the impact of pollution on NPF. This prompted us to examine the dependence of particle number concentration from both campaigns, the results of which are shown in the graph below. This shows a strong correlation of number concentration with CO in both campaigns, with reasonable agreement in the region of overlapping CO concentrations. StratoClim (SASM) N6 concentrations are systematically higher than ACCLIP (both SASM and EASM) for a given CO range. While this could be composition driven, results from ACCLIP EASM and SASM show no systematic differences. This suggests that differences in sampling strategy and time since convection may be responsible for the higher StratoClim concentrations.

We include this new figure as an additional panel in ED Fig 6. With the given addition to the figure caption. This is referenced in the text at lines 107-108, where we had previously referred only to ACCLIP observations we now state:

ACCLIP and StratoClim observations show a strong correlation between observed particle number concentration and CO mixing ratio

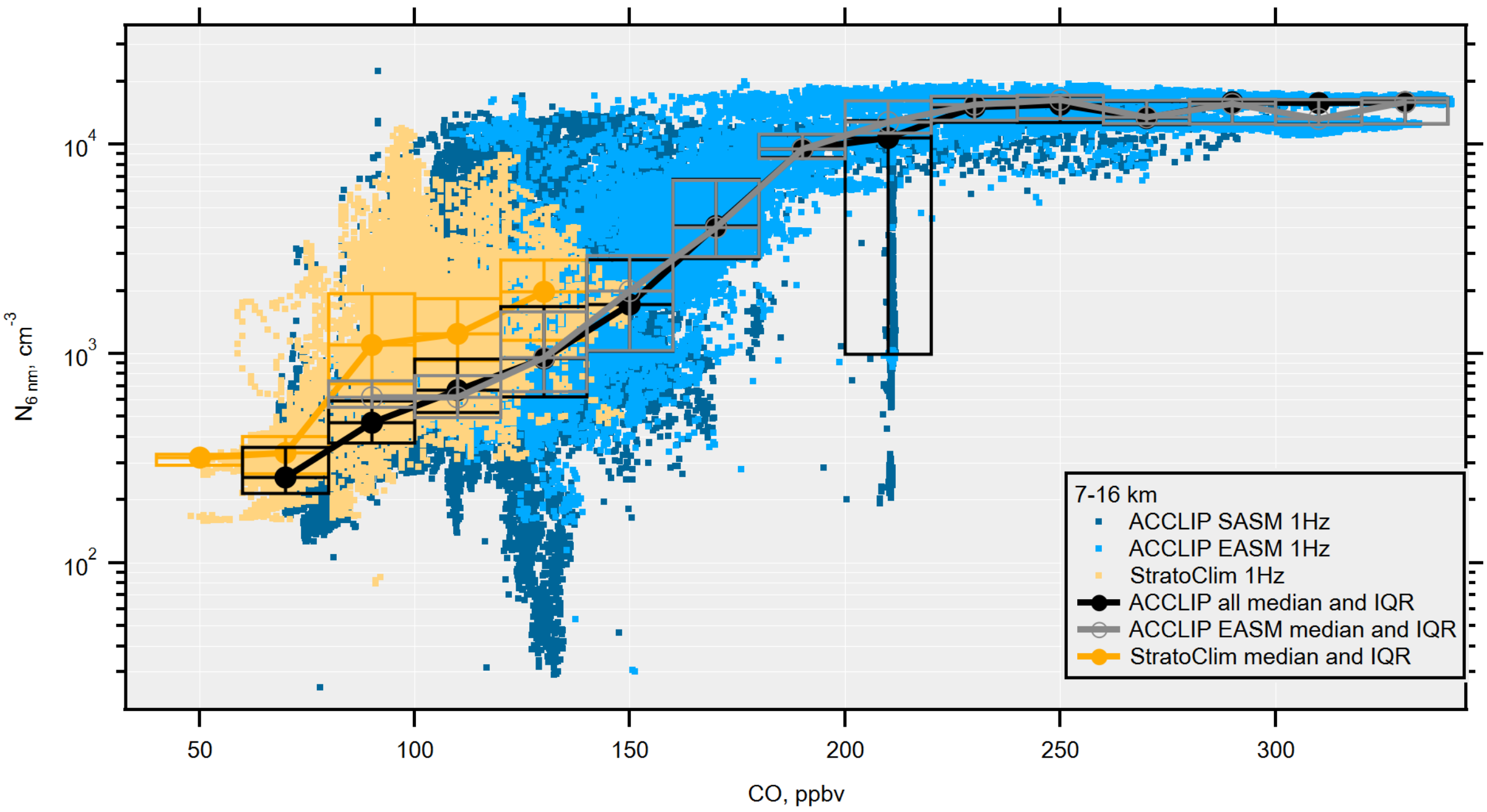

(d) shows the 1 hz observations together with the median and interquartile range (IQR) total number concentration above 6 nm between 7 and 16km as a function of CO from ACCLIP SASM and EASM (dark and light blue 1Hz black and grey statistics respectively) and StratoClim (light orange 1Hz, dark orange statistics). Statistics are only shown for CO bins with more than 5 minutes of data.

We have elaborated on the emissions differences between EASM and SASM in the supplementary discussion section 2 as follows:

A recent study by Eppers, et al. [25] *compared the chemical characteristics of aerosol particles from SASM and EASM during ACCLIP using the hybrid aerosol mass spectrometer ERICA. Their findings showed that the mass fractions of sulfate and organic compounds were higher in EASM air relative to SASM air, while ammonium nitrate was lower. The authors hypothesized that elevated SO_2 and volatile organic compound (VOC) emissions in eastern China could explain these compositional differences. This hypothesis is supported by SO_2 observations from ACCLIP, which indicated higher SO_2 levels in air masses influenced by EASM convection compared to those influenced by SASM*[9].

However, in absolute terms, the mass concentrations of aerosol particles measured by ERICA were lower in the UT for EASM compared to SASM[25]*. This discrepancy was attributed to the relatively fresh nature of the EASM convective outflow sampled during ACCLIP. Specifically, the newly formed particles in the UT may not have had sufficient time to grow into the detectable size range of ERICA in the accumulation mode.*

This is referred to in the main text at line 49 as follows:

The observations show similar spatial extent and number concentrations in both ACCLIP and StratoClim despite substantial interannual variation in the ASM and sampling locations within the region (Supplementary Discussion section 2).

9. The authors have supplemented the temporal evolution of SO2 in Fig. 2 but have not provided dynamic change data for organic components. As potential precursors for nucleation and growth, the post-convection concentration changes of organic components are crucial for understanding NPF mechanisms. Currently, only the statement that “organic components may contribute” is provided without supporting observational data.

We have revised our discussion of organic contribution to NPF as detailed in the response to reviewer 1 point 5.

10. The authors have provided data on changes in scattering coefficients and radiative forcing efficiency in the supplementary materials but have not clarified whether these changes reach a “significantly impactful” level for climate. As the reviewer noted in the early version of the paper that shows the radiative-related impacts in Extended Data Fig. 8 are not significant, yet the authors have not provided quantitative validation (e.g., comparison with contributions from other climate factors) to confirm the actual climatic significance of these impacts.

The radiative impacts as a function of particle size from ED Fig. 9g (original manuscript ED Fig 8) depend on the size distribution present (not shown). We see now that this panel is confusing in its presentation and therefore remove it and refer to the EMAC modelling and discussion to show the climate relevant impacts as discussed above. The new ED Fig. 9 and caption are as follows:

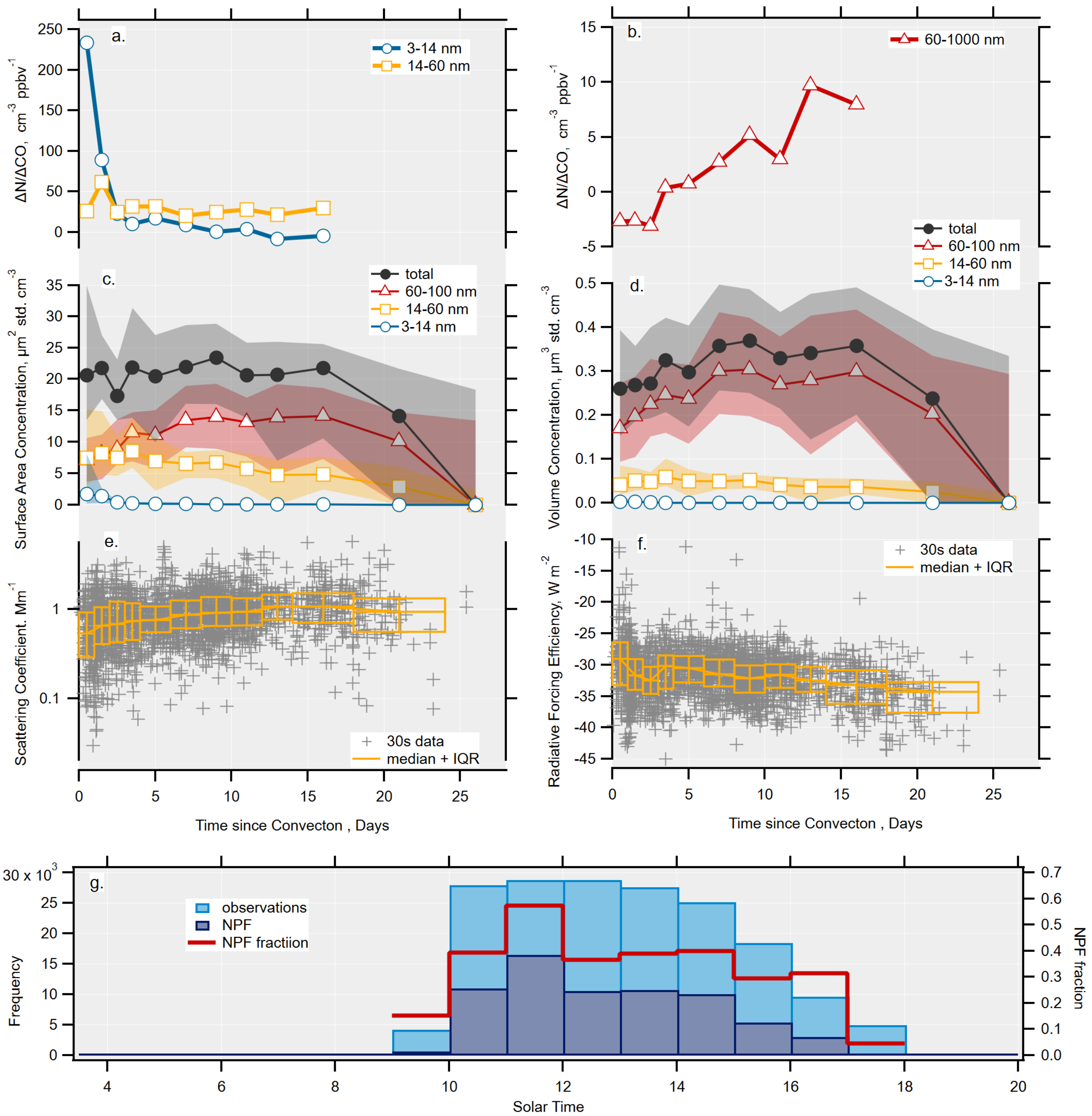

Extended Data Figure 10. *Additional ACCLIP aerosol properties. Normalised excess mixing ratio (NEMR) of number concentrations of particles with diameters (a) 3-14 nm (blue, circles) and 14-60 nm (orange, squares), and (b) 60-1000 nm (red, triangles). The axis for 60-1000 nm diameter particles is on a larger scale to make visible the increase in particle number concentration in this size range. Surface area (c) and volume (d) concentrations observed in ASM outflow on ACCLIP, total, and by aerosol mode as a function of time since convection. e) scattering coefficient and (f) radiative forcing efficiency as a function of time since convection, 30s data in grey, median and interquartile ranges in blue. g) Histogram of solar hour of day when ACCLIP observations were taken (light blue bars) and when NPF was indicated (dark blue bars). The fraction of observations in each hour that showed NPF is shown in the red line.*

We have also removed the reference to this in supplementary discussion section 4.

11. The authors have revised the section title and added CCN-related references but have not elaborated on key information such as the activity of NPF particles as CCN (e.g., critical

supersaturation) and their potential impacts on cloud droplet number concentration. Current discussions remain qualitative ("particles can act as CCN") without specific analyses linking to cloud properties.

We have added an additional panel to ED Fig. 5 showing the impact on CCN number concentration as discussed in the answer to point 6 above.

Reviewer #2 (Remarks to the Author):

I greatly appreciate that the authors have thoroughly addressed my comments. I recommend publication after clarification of their response to my original major comment #1.

Zhang et al. focused on air masses not influenced by pollution or continental emissions to isolate the impact of stratospheric air intrusion (SAI). As a result, the criteria used in Zhang et al. identify "clean" air masses influenced by SAI, but they do not necessarily identify polluted air masses influenced by SAI, which likely have higher CO.

Extended Data Figure 7 shows that, in relatively clean air, the highest N_6nm values were observed in SAI air masses rather than in COF. The results also convincingly show that SAI alone cannot explain the largest particle number concentrations and that lofted pollution is the main driver. That said, the results do not exclude the possibility that mixing between descending stratospheric air and lofted pollution could contribute to high particle concentrations. Similar mixing between stratospheric air and convectively lofted "clean" air was noted in Zhang et al. I recommend that the authors clarify these points in the paper.

The authors have addressed all my other comments thoroughly and satisfactorily.

We are glad our investigation of SAI address the reviewers original comment, and grateful for their input regarding the CO concentrations. On reflection we agree that the same CO limits should not be used for ASM and remote observations to determine SAI and therefore have redone our analysis to account for this. We modify ED Fig 7 (see below, further discussion is presented above the in response to reviewer 1 point 3), and adjust the text at lines 606-628 in the methods as follows:

Stratospheric Air Intrusions and Temperature Effects

The potential role of stratospheric air intrusions (SAI) to increase NPF is explored following Zhang et al[88], which defines SAI ***in less polluted conditions*** *as low water vapour (10-400 ppm), low CO (< 100 ppb) and high ozone (70-100 ppb), and convective outflow (COF) as high water vapour (300-4000 ppm) and low ozone (30-70 ppb), or high CO (> 100 ppb).* ***Since ASM observations are strongly influenced by anthropogenic emissions, we neglect any prior CO limits and examine ASM outflow (altitudes above 7 km) as a function of H_2O and O_3, with limits of 400 ppm and 70 ppb respectively (ED Fig 7). SAI influenced airmasses (high O_3 and low H_2O) dominate the ACCLIP observations, while pure convective outflow (COF) was rarely observed at altitudes above 7 km (ED Fig. 7f). Within SAI influenced airmasses, NPF occurred in over 50% of polluted airmasses (CO > 100 ppb) and 14% pf clean airmasses (CO < 100 ppb) (ED Fig. 7f). When we examine strong NPF events (N6 > 500 cm^{-3}) (ED Fig. 7e) this is even more pronounced. In non-SAI airmasses, non-polluted air was rarely observed, but NPF and strong-NPF frequently occurred in these polluted airmasses (ED Fig. 7 e, f). These observations show that SAI may influence ASM outflow NPF but cannot alone explain the observed frequency and strength of NPF. Pollution is the main driver.***

Temperature profiles of the ACCLIP UT observations (Extended Data Fig. 7a) show that NPF, and especially that resulting in the highest number concentrations, is not occurring at the lowest temperatures.

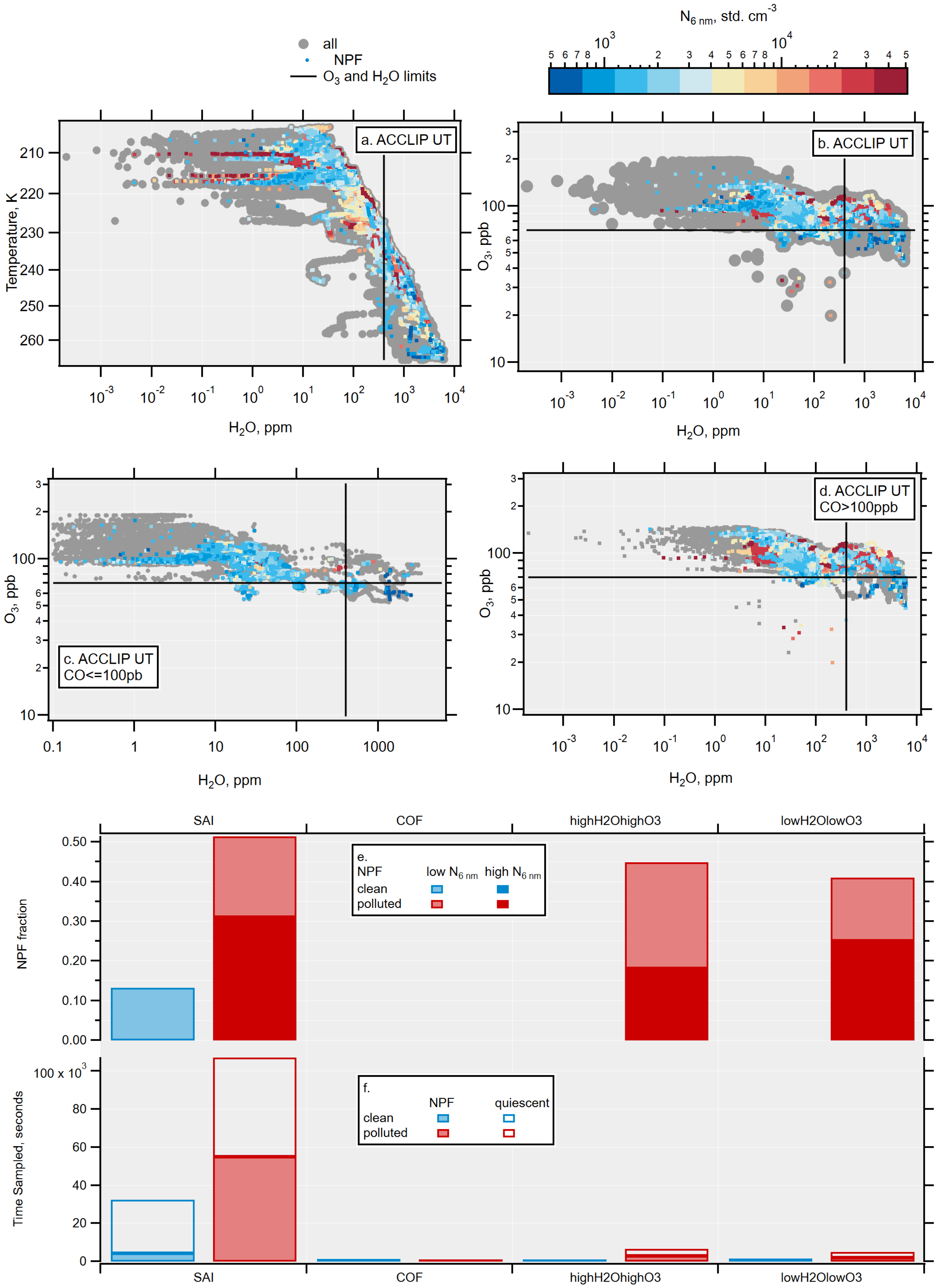

Extended Data Figure 7. Examining the influence of stratospheric air intrusions (SAI) and temperature on NPF in ASM with ACCLIP observations. 4 high altitude (above 7 km) regions defined by threshold

mixing ratios of water vapour (H2O) and ozone (O3), and quantified as polluted or clean by threshold CO mixing ratios. Threshold values are 100 ppbv CO, 400 ppmv H2O, and 70 ppbv O3. SAI is classified as high O3, low H2O, and COF as low O3, high H2O following Zhang et al[90] (see Methods). 1 Hz data indicating NPF (coloured dots, with colours indicating the number concentration of particles greater than 6 nm at standard temperature and pressure), and all 1 Hz data (grey dots) from ACCLIP are examined. The back lines on panels a-d indicate threshold O_3 and H_2O mixing ratios, or convective outflow (COF: low O_3, high H_2O). a) temperature and H_2O distribution. b) O_3 and H_2O distributed for all data, c) low CO data, and d) high CO data. e) the fraction of clean (blue, low CO) and polluted (red, high CO) airmasses within each sampling region that showed statistically significant makers of NPF. Darker shading indicates NPF events with high number concentration (N6>=5000 cm-3 STP) and lighter shading indicates NPF events with low number concentrations (N6<5000 cm-3 STP). f) the total number of 1Hz observations made in clean (blue, low CO) and polluted (red, high CO) airmasses in each sampling region, with shaded bars indicating observations with statistically significant NPF and unshaded bars indicating no statistically significant NPF.

We have removed our statement about SAI influence at lines 128-130 in the previous version and replaced it with the following at lines 144-150:

Most of the airmasses sampled in ASM outflow on ACCLIP were somewhat influenced by stratospheric air intrusions (SAI, see Methods, Extended Data Fig. 7), and the mixing between descending stratospheric air and lofted pollution may contribute to the high particle concentration observed in ASM outflow as has been observed in less polluted convective regions [12]. Within SAI influenced airmasses, more frequent NPF and stronger NPF (high number concentrations) are associated with higher CO concentrations, supporting the conclusion that lofted pollution as the main NPF driver in ASM outflow (ED Fig. 7 e, f).

Furthermore, given the complexity of defining SAI in this polluted region, we remove the SAI delineation in Fig 1 and remove the following from the caption:

~~*Panel a also highlights in red dots and dark red crosses ACCLIP observations identified as influenced by stratospheric air intrusions (SAI, see Methods) and therein those indicating NPF respectively.*~~

Reviewer #3 (Remarks to the Author):

My review of the authors' response indicates that while they have addressed several methodological details by clarifying statistical definitions, temporal analysis, and removing problematic comparisons. Regarding the nucleation pathways, the authors employ modeling work to compensate for the lack of observations of key species. However, they have not addressed the fundamental question of scientific novelty and significance. This should be decided by the editor.

We are grateful for your review.

Reviewer #4 (Remarks to the Author):

I co-reviewed this manuscript with one of the reviewers who provided the listed reports. This is part of the Nature Communications initiative to facilitate training in peer review and to provide appropriate recognition for Early Career Researchers who co-review manuscripts.

We are grateful for your review.

Corrections to language and clarity

Having addressed the individual points raised by the reviewers above, we have found it helpful to adjust the emphasis in the introductory paragraph, abstract and non-technical summary to better reflect the evolved manuscript. The introductory paragraph at lines 39-44 now reads:

Combined evidence from observations and targeted Earth-system model sensitivity studies indicates that pollution lofted by the Asian Summer Monsoon enhances new particle formation in convective outflow, and that the resulting particles grow to sizes large enough to influence climate.

The abstract at lines 8-14 now reads:

In-situ observations of aerosol size distributions and trace gases combined with targeted modelling show that new particle formation occurs in outflow from ASM convection across the entire ASM region, and indicate that pollutants transported from the surface increase the concentrations of particles formed aloft. These newly formed particles grow to sizes at which they have potential to enhance scattering of radiation, influence cirrus cloud properties and act as cloud condensation nuclei after descending to lower levels.

The non-technical summary at lines 16-23 now reads:

Deep storms during the Asian Summer Monsoon loft polluted air from the surface to 12-18 km altitudes. Observations from aircraft combined with modelling indicate that the pollutants increase the number of aerosol particles that form at high altitude, and that these particles grow over a period of weeks to sizes where they have potential to impact climate. Many of these particles stay below the stratosphere and descend over time. Studies concerning the impacts of Asian summer monsoon on climate often focus on the stratosphere, but this study shows it is important also to consider aerosol impacts below the stratosphere.

References

1 Bergman, J. W., Fierli, F., Jensen, E. J., Honomichl, S. & Pan, L. L. Boundary layer sources for the Asian anticyclone: Regional contributions to a vertical conduit. *Journal of Geophysical Research: Atmospheres* **118**, 2560-2575, doi:https://doi.org/10.1002/jgrd.50142 (2013).

2 Clemens, J. *et al.* A multi-scenario Lagrangian trajectory analysis to identify source regions of the Asian tropopause aerosol layer on the Indian subcontinent in August 2016. *Atmos. Chem. Phys.* **24**, 763-787, doi:10.5194/acp-24-763-2024 (2024).

3 Vogel, B., Günther, G., Müller, R., Grooß, J. U. & Riese, M. Impact of different Asian source regions on the composition of the Asian monsoon anticyclone and of the extratropical lowermost stratosphere. *Atmos. Chem. Phys.* **15**, 13699-13716, doi:10.5194/acp-15-13699-2015 (2015).

4 Bucci, S. *et al.* Deep-convective influence on the upper troposphere–lower stratosphere composition in the Asian monsoon anticyclone region: 2017 StratoClim campaign results. *Atmos. Chem. Phys.* **20**, 12193-12210, doi:10.5194/acp-20-12193-2020 (2020).

5 Oh, H., Ha, K.-J. & Jeong, J.-Y. Identifying Dynamic and Thermodynamic Contributions to the Record-Breaking 2022 Summer Extreme Rainfall Events in Korea. *Asia-Pacific Journal of Atmospheric Sciences* **60**, 387-399, doi:10.1007/s13143-023-00334-4 (2024).

6 Pan, L. L. *et al.* The Asian Summer Monsoon Chemical and Climate Impact Project (ACCLIP): An Overview. *Journal of Geophysical Research: Atmospheres* **130**, e2025JD044417, doi:https://doi.org/10.1029/2025JD044417 (2025).

7 Pan, L. L. *et al.* East Asian summer monsoon delivers large abundances of very short-lived organic chlorine substances to the lower stratosphere. *Proceedings of the National Academy of Sciences* **121**, e2318716121, doi:doi:10.1073/pnas.2318716121 (2024).
8 Zhang, P., Wang, B., Wu, Z., Jin, R. & Cao, C. Intensified gradient La Niña and extra-tropical thermal patterns drive the 2022 East and South Asian “Seesaw” extremes. *npj Climate and Atmospheric Science* **7**, 47, doi:10.1038/s41612-024-00597-2 (2024).
9 Smith, W. P. *et al.* Transport by Asian Summer Monsoon Convection to the Upper Troposphere and Lower Stratosphere During ACCLIP (2022). *Journal of Geophysical Research: Atmospheres* **130**, e2024JD042732, doi:https://doi.org/10.1029/2024JD042732 (2025).
10 Weigel, R. *et al.* In situ observation of new particle formation (NPF) in the tropical tropopause layer of the 2017 Asian monsoon anticyclone – Part 1: Summary of StratoClim results. *Atmos. Chem. Phys.* **21**, 11689-11722, doi:10.5194/acp-21-11689-2021 (2021).
11 Weigel, R. *et al.* In situ observation of new particle formation (NPF) in the tropical tropopause layer of the 2017 Asian monsoon anticyclone – Part 2: NPF inside ice clouds. *Atmos. Chem. Phys.* **21**, 13455-13481, doi:10.5194/acp-21-13455-2021 (2021).
12 Zhang, J. *et al.* Stratospheric air intrusions promote global-scale new particle formation. *Science* **385**, 210-216, doi:doi:10.1126/science.adn2961 (2024).
13 Wang, M. *et al.* Synergistic HNO3–H2SO4–NH3 upper tropospheric particle formation. *Nature* **605**, 483-489, doi:10.1038/s41586-022-04605-4 (2022).
14 Xenofontos, C. *et al.* The impact of ammonia on particle formation in the Asian Tropopause Aerosol Layer. *NPJ Clim Atmos Sci* **7**, 215, doi:10.1038/s41612-024-00758-3 (2024).
15 Santee, M. L. *et al.* A comprehensive overview of the climatological composition of the Asian summer monsoon anticyclone based on 10 years of Aura Microwave Limb Sounder measurements. *Journal of Geophysical Research: Atmospheres* **122**, 5491-5514, doi:https://doi.org/10.1002/2016JD026408 (2017).
16 Rollins, A. W. *et al.* The role of sulfur dioxide in stratospheric aerosol formation evaluated by using in situ measurements in the tropical lower stratosphere. *Geophysical Research Letters* **44**, 4280-4286, doi:https://doi.org/10.1002/2017GL072754 (2017).
17 Aneja, V. P., Schlesinger, W. H., Li, Q., Nahas, A. & Battye, W. H. Characterization of the Global Sources of Atmospheric Ammonia from Agricultural Soils. *Journal of Geophysical Research: Atmospheres* **125**, e2019JD031684, doi:https://doi.org/10.1029/2019JD031684 (2020).
18 Behera, S. N., Sharma, M., Aneja, V. P. & Balasubramanian, R. Ammonia in the atmosphere: a review on emission sources, atmospheric chemistry and deposition on terrestrial bodies. *Environ Sci Pollut Res Int* **20**, 8092-8131, doi:10.1007/s11356-013-2051-9 (2013).
19 Chen, D. *et al.* Airborne measurements of BrO and the sum of HOBr and Br2 over the Tropical West Pacific from 1 to 15 km during the CONvective TRansport of Active Species in the Tropics (CONTRAST) experiment. *Journal of Geophysical Research: Atmospheres* **121**, 12,560-512,578, doi:https://doi.org/10.1002/2016JD025561 (2016).
20 Ji, Y. *et al.* A vacuum ultraviolet ion source (VUV-IS) for iodide–chemical ionization mass spectrometry: a substitute for radioactive ion sources. *Atmos. Meas. Tech.* **13**, 3683-3696, doi:10.5194/amt-13-3683-2020 (2020).
21 Zhu, Y. *et al.* Evaluating the Importance of Nitrate-Containing Aerosols for the Asian Tropopause Aerosol Layer. *Journal of Geophysical Research: Atmospheres* **129**, e2024JD041283, doi:https://doi.org/10.1029/2024JD041283 (2024).
22 Yu, P. *et al.* Abundant Nitrate and Nitric Acid Aerosol in the Upper Troposphere and Lower Stratosphere. *Geophysical Research Letters* **49**, e2022GL100258, doi:https://doi.org/10.1029/2022GL100258 (2022).
23 Cziczo, D. J. & Abbatt, J. P. D. Ice nucleation in NH4HSO4, NH4NO3, and H2SO4 aqueous particles: Implications for cirrus cloud formation. *Geophysical Research Letters* **28**, 963-966, doi:https://doi.org/10.1029/2000GL012568 (2001).

24 Wagner, R. *et al.* Solid Ammonium Nitrate Aerosols as Efficient Ice Nucleating Particles at Cirrus Temperatures. *Journal of Geophysical Research: Atmospheres* **125**, e2019JD032248, doi:https://doi.org/10.1029/2019JD032248 (2020).
25 Eppers, O. *et al.* Regional contributions to the chemical composition of the Asian tropopause aerosol layer (ATAL). doi:10.22541/essoar.175130383.35260852/v1 (2025).

Reviewer Comments 27.04.2026

Reviewer #1 (Remarks to the Author):

While the authors have addressed and responded to most of my previous comments and concerns, the proposed nucleation mechanism—NH_3–HNO_3–H_2SO_4 nucleation—is still largely inferred from model simulations, and in my view remains debatable; further observational constraints in future will likely be needed to verify it.
As the authors themselves stated in their reply: "...Organics are also abundant in the observed larger particles in ASM outflow (Extended Data Fig. 8, Extended Data Fig. 5g) and may be involved in nucleation and/or growth, similarly to in remote tropical convection 17...."
Accordingly, it cannot be reasonably concluded that organic nucleation is unnecessary to explain the observed particle composition and number concentrations solely based on modeling results.

We have removed the statement that organic nucleation is unnecessary to explain the observations. Lines 162-164 now read: "*Organics are also abundant in the observed larger particles in ASM outflow (Fig. 7, Fig. 6g) and may be involved in nucleation and/or growth, similarly to in remote tropical convection*[17]*. However, so far, no mechanisms have been proposed for this.*"

To better clarify the limitations of this work we include the sentence "*Future observations of the chemical composition of clusters and smallest particles would be necessary to verify all nucleation mechanisms involved.*" at lines 166-167.

In addition, one of my main concerns is that the authors emphasize their study focuses on how anthropogenic pollutants influence NPF events in the upper troposphere and the corresponding climatic effects. I do recognize that they have examined CCN number concentration changes from model simulations. However, the main text of the manuscript has not been updated or revised accordingly; the current version remains largely identical to the original submission, with no apparent improvement in scientific content or presentation. As Reviewer 3 pointed out, the authors have not adequately addressed the fundamental question of scientific novelty and significance.

As mentioned in the authors' response, previous modeling studies have already highlighted the role of monsoon convection in lifting NH_3 into the UTLS. Xenofontos et al. (30) used EMAC simulations over the South Asian monsoon and found that UT NPF rates during convective days were 1–2 orders of magnitude higher than during quiescent days, driven by NH_3 uplift. Xenofontos et al. (31) extended this globally and showed that anthropogenic NH_3 emissions coupled with convective transport enhance UTLS NPF rates. This study therefore needs to clearly articulate what new insights and findings have been achieved beyond those previous studies.

We have now made major revisions to the main text. Much of the discussion of context from previous work has been moved to the new introduction section, leaving the new results from this study, complete with caveats on what is seen from observations and what comes from modelling in the Results and Discussion Section.

Details of impact on climate beyond the direct results from EMAC modelling in this study are addressed in the introduction at lines 48-50 thus:

"These aerosols impact climate via direct radiative effects[9,13]*, providing increased surface area for heterogeneous chemistry*[14]*, affecting the properties of UT cirrus clouds, either through homogeneous ice nucleation, or heterogenous ice-nucleation of ammonium nitrate aerosol*[14-18]*, and affecting the properties of warm and mixed-phase clouds as CCN after descent*[16]*."*

Similarly, introduction of the synergistic nucleation mechanism is removed from the main text (lines 156-161) and instead presented in the introduction at lines 61-27 as:

"Synergistic NH_3-HNO_3-H_2SO_4 nucleation, a fast UTLS nucleation and growth mechanism has been proposed from laboratory experiments and modelling[15,16]. Prior observations of high mixing ratios of ammonia in ASM convective outflow[35,36] support the idea that this mechanism is of relevance to ASM convective outflow, and but this has not yet been verified by observations. Modelling studies predict that during the ASM days with convection produce UT NPF rates that are one to two orders of magnitude higher than quiescent days, driven by NH_3 uplift[30], and that globally, anthropogenic NH_3 emissions when combined with convective transport enhance UTLS NPF rates[31]."

The abstract now clearly delineates between prior knowledge (lines 28-26):

"The Asian Summer Monsoon (ASM) lofts air from polluted boundary layer regions to the upper-troposphere and lower-stratosphere. High concentrations of aerosols have been observed in ASM outflow, which influence climate by interacting with radiation and clouds and affecting atmospheric composition. Prior observations indicate that small particles form in ASM convective outflow. Laboratory and modelling studies hypothesise the importance of lofted ammonia in this process and growth of the particles to sizes where they can interact with radiation and clouds. The role of anthropogenic emissions in ASM new particle formation and their growth have not previously been demonstrated from observations."

And new findings (lines 36-41):

"Here we use in-situ observations, trajectory and Earth-system modelling to show the likely role of pollution in enhancing ASM outflow new particle formation. We show that the particles grow to sizes where they can interact with clouds and radiation, and are transported over large areas within the troposphere and stratosphere."

The introduction expands on what is new from this study in lines 73-84:

"*We assess the impact of pollution on particle formation in the upper troposphere by analysing in-situ measurements from two aircraft campaigns, the Asian Summer Monsoon Chemical and Climate Impact Project (ACCLIP)[41,42] and Stratospheric and upper tropospheric processes for better climate predictions (StratoClim)[34,43,44], combined with integrated trajectory modelling and satellite observations to diagnose the history and fate of the observed airmasses, and targeted Earth-system model sensitivity studies. We show that NPF occurs with high frequency and intensity in ASM convective outflow within the first two days following convection, and that this phenomenon occurs over the entire ASM region. We provide evidence that pollution lofted by the ASM likely increases the number of particles formed in the upper troposphere (UT), and that newly formed particles growth within the ASM region to sizes where they can influence climate in about a week. We demonstrate that this phenomenon is regional in scale and is associated with increased aerosol optical depth (AOD) and cloud condensation nuclei (CCN) concentrations.*"

The introduction has new text explaining why evidence for and quantification of growth is import and new in lines 68-72 as follows:

"Particles form from the gas phase at only a few nm in diameter[35] and must grow to diameters above around 60 or 100 nm to significantly influence cloud properties or interact with radiation respectively e.g. [36,37,38]. Therefore, the growth rate of these particles up is of critical importance in determining their survival probability and fate[39,40]. Direct evidence of this growth, and quantification of the growth rate have not previously been shown."

Lines 181-186 of the main text have been changed to remove discussion of climate impacts (as mentioned above this is now done in the introduction) and focus on the novel findings regarding growth as follows:

"ACCLIP observation provide evidence that particles formed in ASM convective outflow grow to diameters larger than 60 nm (Fig. 9), making interactions with clouds and radiation possible, and providing significance surface area to impact heterogeneous chemistry (Supplementary Fig. 2 c, e,f)."

Statements above potential impact of accumulation mode aerosol have also been removed from lines 213-219, and summaries instead as mentioned above in the introduction.

The conclusions section has been removed in line with the journal format, and the relevant points included into the introduction and results/discussion. This has all been quoted above apart from statements relating to the fate of the particles, which are now added to the end of the paragraph presenting our forward trajectory modeling results as lines 224-226 as

"Therefore, while previous literature has focused heavily on the ASM as a source of particulate matter maintaining the ATAL, the effects of these particles on clouds and radiation in the troposphere must also be considered when evaluating the overall climate impacts of the ASM."

We have also added context to the results of the forward trajectory modelling in lines 213-214 as

"This local cross-tropopause transport is a significant source of ammonium nitrate and organic aerosol abundance in the stratosphere[44,51,52]".

Beyond the changes detailed above to direct address the reviewer comments, the following changes have been made following directions from the journal:

- Extended Data have been removed. Most extended data figures have been made into main display items (1-10) instead of the original 4, with some moved to the supplementary along with the relevant methods for those figure.
- Additional methods details are included for the inversion procedure, AMS instrumentation, and meteorological observations.
- Subheadings have been removed
- Figure captions have been clarified.
- Figure colours have been changed to be colourblind friendly in places where they were not previously
- References have been combined

Upper Troposphere Aerosol Formation Enhanced by Monsoon Lofted Pollution

This study evaluates the impact of pollution on the formation of new particles in upper convection based on the particle size distribution observed in the convective outflow in the "Asian Summer Monsoon Chemistry and Climate Impact Project" (ACCLIP) and "StratoClim for Improving Climate Prediction". The measurement results presented here do reveal some interesting features that may provide new insights of the impact of air pollutants from Asia on the formation of new particles in upper troposphere. While it has been known that NPF can take place at very UTLS, little has been known on the anthropogenic contribution to such events especially over the monsoon region in Asia. If this were demonstrated, it would have significant implications and impact on both atmospheric environment and climate especially over the Asian monsoon region where climate have been intertwined with air pollution, as demonstrated in many studies (but few were referred to in this manuscript). As pointed below, however, I have serious concerns with the major findings of the study which aren't rigorous enough to recommend it for publication in the current form.

Major comments: circumstantial evidence

However, the major findings of the study are (1) the NPF present at UTLS, 2) they are formed from gas pollutants brought up by monsoon circulation, 3) the NPF have significant impact on climate. While all these arguments are sound to some degree, but they are also handwaving with little rigorous analyses and modeling to really prove, especially for 2) and 3). To support 2), CO was used as a tracer to differentiate polluted and cleaner air masses. To substantiate 2), one would need to propose a specific NPF mechanism and apply their observation data to simulate and compare against observations. One cannot readily jump to a conclusion that the NPF was caused by the anthropogenetic precursors over that region. It'd need to demonstrate the pathway of the transport of the gases all the way up to those altitudes where environmental conditions are just adequate to form new particles, or it could NPFs that already exist

in the lower atmosphere and brought up to the higher altitudes.

Likewise, the manuscript lacks any quantitative analysis of any specific events to support the argument of significant impact of the formation of new particles on climate. I don't see any convincing evidences to support their argument that NPF (1) affects the characteristics of cumulus clouds, (2) affects heterogeneous chemistry in the troposphere and stratosphere, in any figures, data in their main text nor SI, although they are generally sound in principle. To convince the impact, it would be necessary to conduct a modeling study based on the observations.

In addition, regarding to the impact on radiation forcing, the authors also stated that the NPF noticeably enhances the scattering of shortwave radiation. Note that scattering is proportional to the collective sections of all aerosol particles. In general, for such tiny particles especially newly formed ones, their scattering ability is negligible According to the Fig 8 in the extended data, it seems that the impact is not that significant. I also suggest move some of the panels in extended data Figure 8 (like Fig. 8e and f) to the main text.

Specific comments:

Line 47: Some explanation needs to be given as to why the number concentration reaches its peak near the potential temperature of 360 K?

Line 62: As mentioned in the text, the definition of NPF events is a significant increase in the observed number concentration between 6-8 nm. However, figure 2 did not clearly shows the characteristics of this particle size range. Conversely, particles between 3-14 nm exhibit a maximum at the initial moment, which seems misleading.

Line 64: The manuscript mentions that significant NPF events can be observed within 2-3 days since the onset of convection, and not particularly afterwards. The particle characteristic information within this interval (time period) should be supplemented. The current version coordinates show that the characteristics of the 2-3 days among 0-28 days are not obvious.

Line 71: Figure 2c shows that condensation sinks have valley values 3 days since convection, while maintaining high values within 2 days since convection. Here the study explains in Line 119, "indicating that the condensation sink is not the controlling factor for NPF, and highlighting the importance of the availability of condensable

vapours in the first days after convection." It is suggested to supplement the evolution of SO_2 and organic components since convection similar to the evolution of particle concentration mentioned in the text. The current result only shows the campaign mean of chemical composition and the mixing ratios with the convective outflow seems insufficient.

Line 135: Regarding climate related impacts, the manuscript focuses on discussing the evolution of particle number size distribution and number concentration since convection, without delving into the discussion of CCN and cloud related factors, which requires further elaboration.

Line 333: Suggestions for the Section of Method, 1) Specific Introduction to the Frequency Distribution of NPF in Observations, 2) adding the description of condensation sink calculation.
